# Straightforward Access to Multifunctional π‐Conjugated P‐Heterocycles Featuring an Internal Ylidic Bond**

**DOI:** 10.1002/anie.202205548

**Published:** 2022-06-23

**Authors:** Thomas Delouche, Elsa Caytan, Marie Cordier, Thierry Roisnel, Grégory Taupier, Yann Molard, Nicolas Vanthuyne, Boris Le Guennic, Muriel Hissler, Denis Jacquemin, Pierre‐Antoine Bouit

**Affiliations:** ^1^ Univ Rennes, CNRS, ISCR—UMR 6226, ScanMAT—UMS 2001 35000 Rennes France; ^2^ Aix Marseille Univ, CNRS, Centrale Marseille, iSm2 Marseille France; ^3^ CEISAM Lab-UMR 6230, CNRS, Nantes University Nantes France

## Abstract

We report the straightforward one‐pot synthesis of novel 5‐ or 6‐membered P‐heterocycles featuring an internal ylidic bond: P‐containing acenaphthylenes and phenanthrenes. The stability of the compounds tolerates post‐functionalization through direct arylation to introduce electron‐rich/poor substituents and the synthetic strategy is also compatible with the preparation of more elaborate polyaromatic scaffolds such as acenes and helicenes. Using a joint experimental (X‐ray analysis, optical and redox properties) and theoretical approach, we perform a full structure–property relationships study on these new platforms. In particular, we show that molecular engineering allows not only tuning their absorption/emission across the entire visible range but also endowing them with chiroptical or non‐linear optical properties, making them valuable dyes for a large panel of photonic or opto‐electronic applications.

## Graphical abstract

A simple synthesis of P‐heterocycles featuring an internal ylidic bond is reported. The stability of the compounds tolerates post‐functionalization (direct arylation) and the strategy is also compatible with the preparation of polyaromatic scaffolds (acenes, helicenes). Molecular engineering allows tuning the optical and redox properties, making them valuable dyes for a large panel of photonic or opto‐electronic applications.

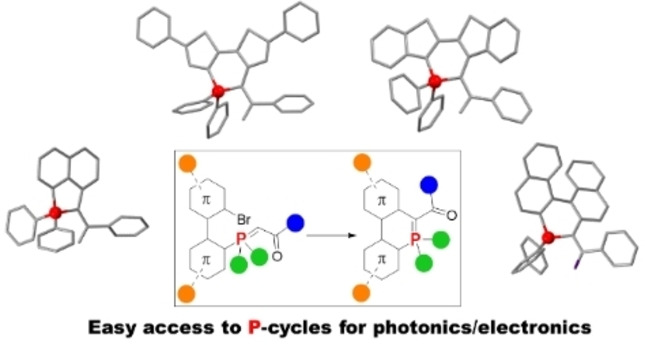

## Introduction

**Figure 1:**
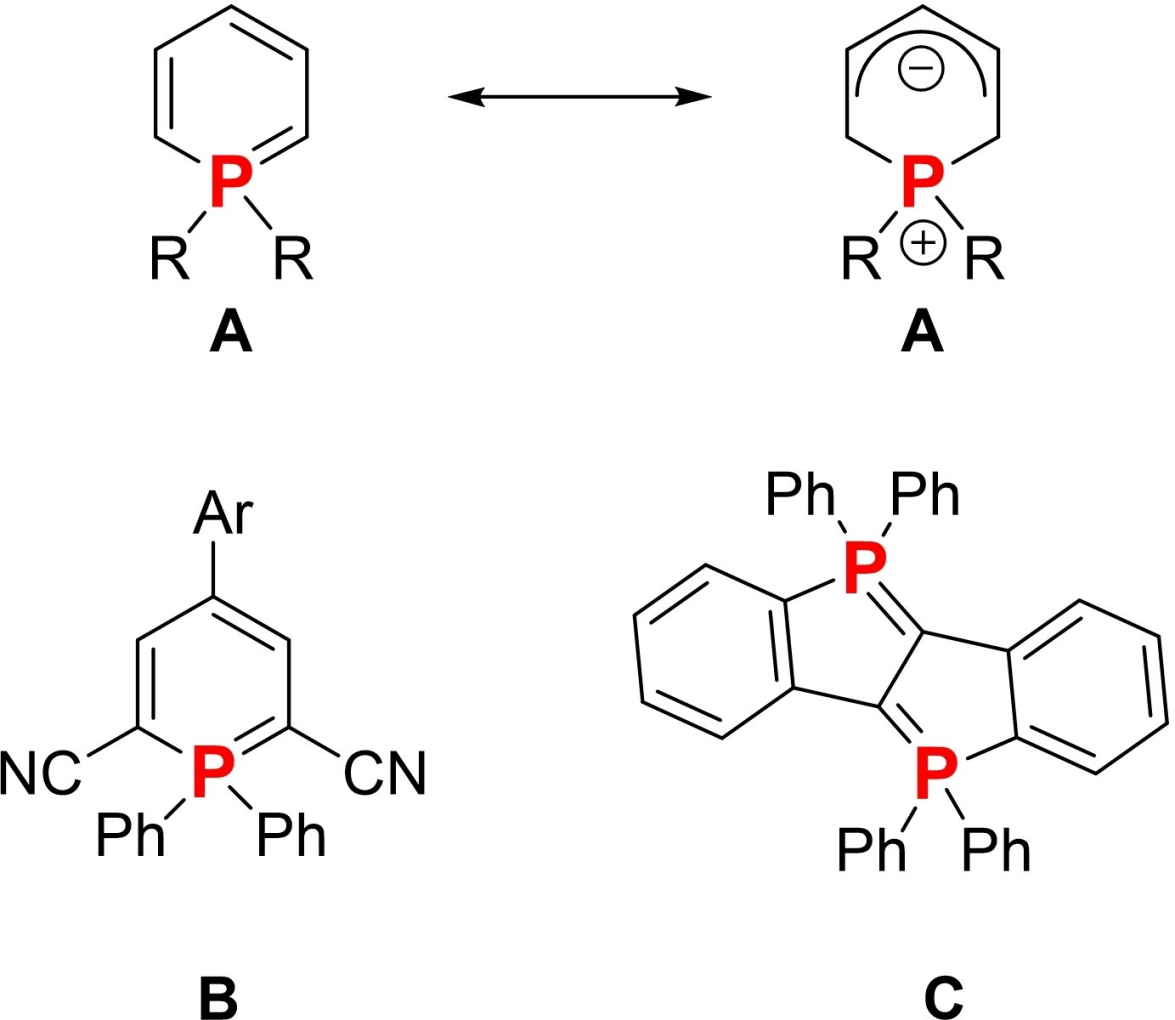
Resonant form of λ^5^‐phosphinine **A**, representative examples of π‐extended λ^5^‐phosphinines **B**, and examples of 5‐membered P‐ring with an internal λ^5^‐P−C bond (**C**).

In the blooming field of functional materials based on organophosphorus π‐conjugated heterocycles,[^1^, ^2^, ^3^, ^4^, ^5^, ^6^, ^7^] little attention has been devoted to phosphacycles featuring an internal P‐ylide. Such a bond was supposed to be too reactive, yet its ability to efficiently delocalize charges was discussed.^[8]^ However, various recent reports on the properties of π‐extended λ^5^‐phosphinines (also called phosphabenzene) have shed a new light on this “old” heterocycle.[^9^, ^10^] In these systems, the bonding topology can be described as a combination between two limit forms: i) a Hückel‐type aromatic system and ii) a cyclic phosphonium ylide with a negatively charged carbon backbone (**A**, Figure 1). The fluorescence of its π‐extended form, associated with its remarkable chemical and thermal stability, allowed it to be used as an emitter in OLEDs or in organic lasers (**B**, Figure 1).[^11^, ^12^, ^13^, ^14^] Regarding the five‐membered ring, limited reports have described the properties of λ^5^‐phospholes or diphospha‐acenes such as **C** (Figure 1).[^15^, ^16^] In these systems the bonding topology results from a combination of resonant forms. Although these recent results highlight the promising optical and redox properties of these “ylidic” P‐heterocycles in the context of photonics, efficient synthetic methods to prepare them remain limited.[^17^, ^18^, ^19^, ^20^, ^21^, ^22^, ^23^]

**Figure 2:**
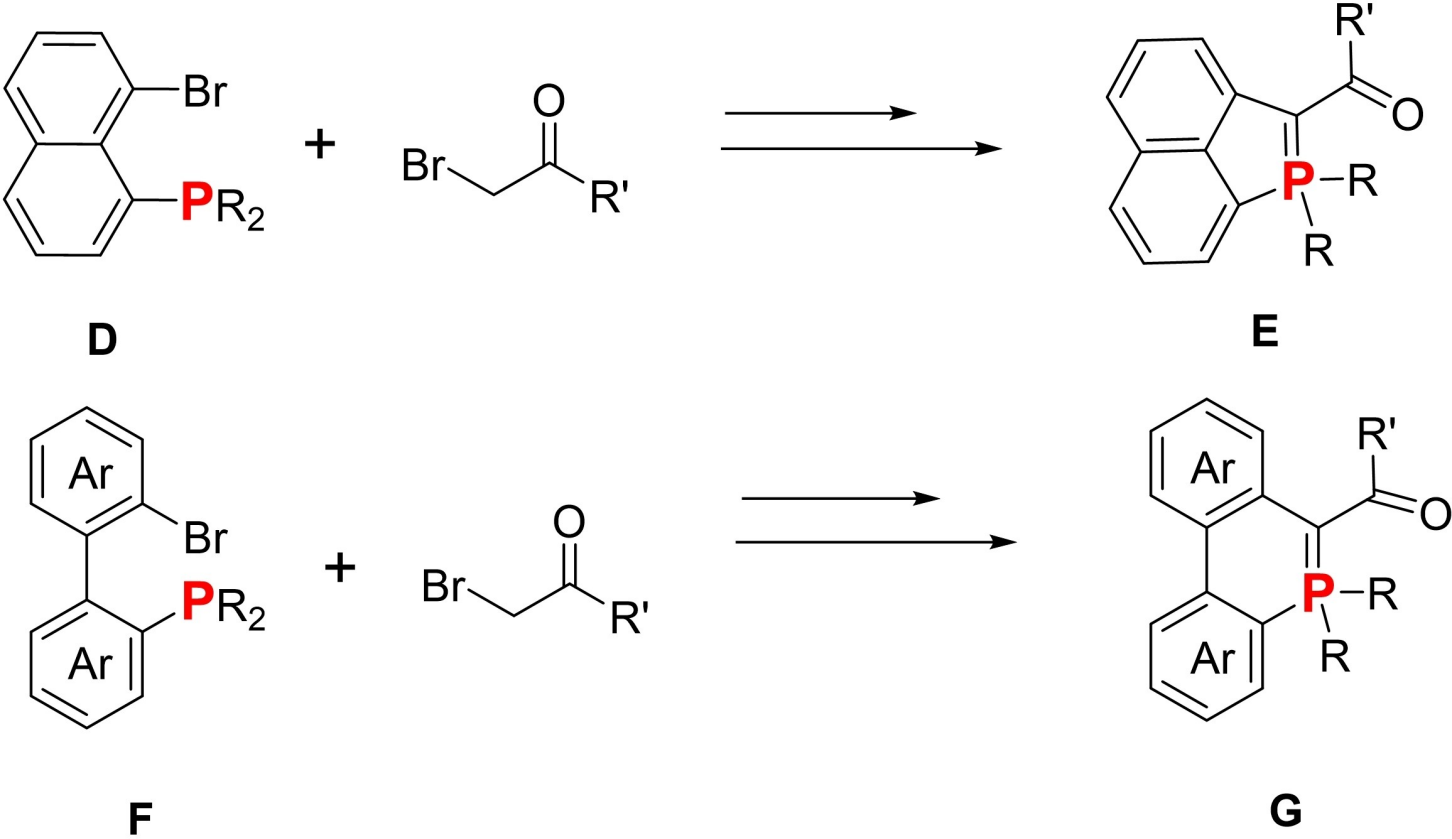
Synthesis of 5‐ and 6‐membered P‐heterocycles featuring an internal λ^5^‐P−C bond **E** and **G**.

Herein, we report a straightforward one‐pot synthesis that allows P‐containing acenaphthylenes or phenanthrenes featuring an internal ylidic bond (**E** and **G**, Figure 2) to be obtained. These compounds are easily obtained through intramolecular cyclization of ylides generated from readily available phosphine **D** and **F** (Figure 2). Such simple heteroaromatic synthesis can be extended to more elaborate P‐containing acenes and helicenes. The molecular engineering of this heterocycle allows tuning their photophysical properties across the entire visible range as well as tuning their redox properties. These modifications were rationalized using DFT methods (see Supporting Information). Finally, we could also endow them with chiroptical or non‐linear optical properties.

## Results and Discussion

**Scheme 1:**
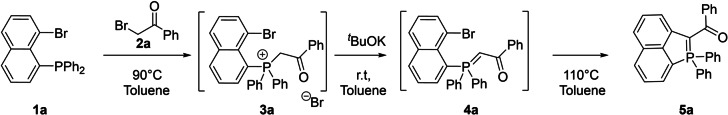
Synthesis of phosphaacenaphthylene **5** **a**.

During our study of the reactivity of bromo‐naphthyl phosphines such as **1** **a** (Scheme 1), we observed that refluxing in situ generated carbonyl‐stabilized phosphonium ylide **4** **a** (^31^P‐NMR=+23.8 ppm) in toluene leads to intramolecular cyclization with the formation of the λ^5^‐phosphaacenaphthylene **5** **a** in good yields (52 %, ^31^P‐NMR=+22.8 ppm).^[24]^ **5** **a** was fully characterized by multinuclear NMR spectroscopy and MS analysis. In particular, the typical ^13^C shift of carbonyl (δ=185.7 ppm) and ylidic C−P (δ=77.8 ppm) are observed. **5** **a** is fully air and moisture stable and can be purified through chromatography on silica. In addition, **5** **a** was characterized by single‐crystal X‐ray diffraction (see below). A related sequence of intramolecular cyclization of an ylide followed by a rearomatization under basic conditions was previously observed by Hayashi et al. in their synthesis of 3‐oxo‐λ^5^‐phosphole.^[25]^ Interestingly, Xu et al. reported that the Pd‐catalysed intramolecular cyclization of related carbonyl‐stabilized phosphonium ylides proceed through Ullman‐type O‐arylation.^[26]^ With our phosphonium, the use of various Pd catalysts (Pd(PPh_3_)_4_, PdCl_2_(PPh_3_)_2_, Pd(OAc)_2_ etc.) did not change the rate of the reaction and only the λ^5^‐phosphaacenaphthylene **5** **a** was formed in similar yields.

**Scheme 2:**
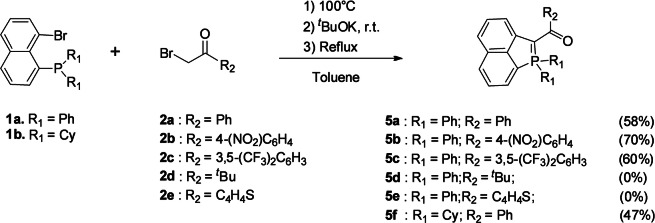
Synthesis of phosphaacenaphthylenes **5** **a**–**f**.

In order to study the scope of the reaction, various arylcarbonyl phosphonium ylides were tested (Scheme 2). Unsurprisingly, the reaction efficiently proceeds with electron‐poor aryls such as **2** **b** and **2 c**. However, no final product was observed in the case of electron‐rich ^t^Bu (**2** **d**) or thienyl (**2** **e**), illustrating the necessity to proceed with a stabilized ylide. This was confirmed as no reaction occured in the absence of ketone on the phosphonium **3** **a**. The use of ester instead of ketone on the ylide led to a mixture of products that we could not separate properly. Finally, the reaction is tolerant to modification of the phosphine as exemplified by the reaction with cyclohexylphosphine (**1** **b**). To summarize, such a methodology allows easy access to a family of phosphaacenaphthylenes featuring an internal ylidic bond. Modifications of the carbonyl substituent as well as the exocyclic P‐substituent can be performed.

**Scheme 3:**
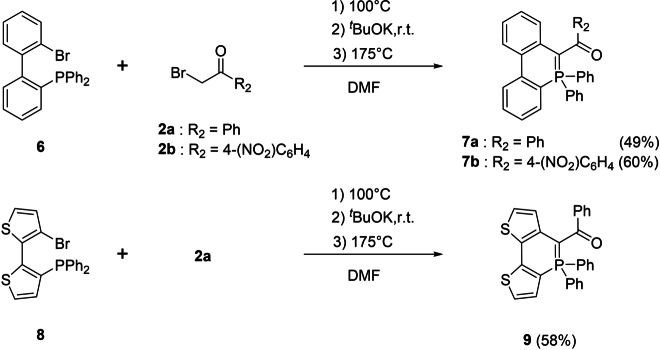
Synthesis of 6‐membered P‐heterocycles **7** and **9**.

We were then interested in extending this strategy toward the synthesis of 6‐membered P‐heterocycles such phosphaphenanthrenes.[^27^, ^28^, ^29^, ^30^, ^31^] The methodology was tested with 2‐(diphenylphosphino)‐2′‐dibromo‐1,1′‐biphenyl **6** (Scheme 3). The thermal reaction only worked when the in situ generated ylide is heated at 175 °C in DMF. In this case, the λ^5^‐phosphaphenanthrenes **7** **a**, **b** are synthesized in moderated yields. When the reaction is performed under catalytic conditions (Pd(PPh_3_)_4,_ 0,05 equiv), the reaction proceeds at 115 °C. If the biphenyl moiety of **6** is replaced by a dithienyl scaffold (**8**), the reaction efficiently proceeds toward λ^5^‐dithienophosphinine **9** (58 %, Scheme 3). It is worth mentioning that similar phosphaalkene based P‐frameworks were already described,[^29^, ^30^] but our new synthetic strategy allows efficiently preparing these 6‐membered P‐heterocycles featuring ylidic bond.

**Figure 3:**
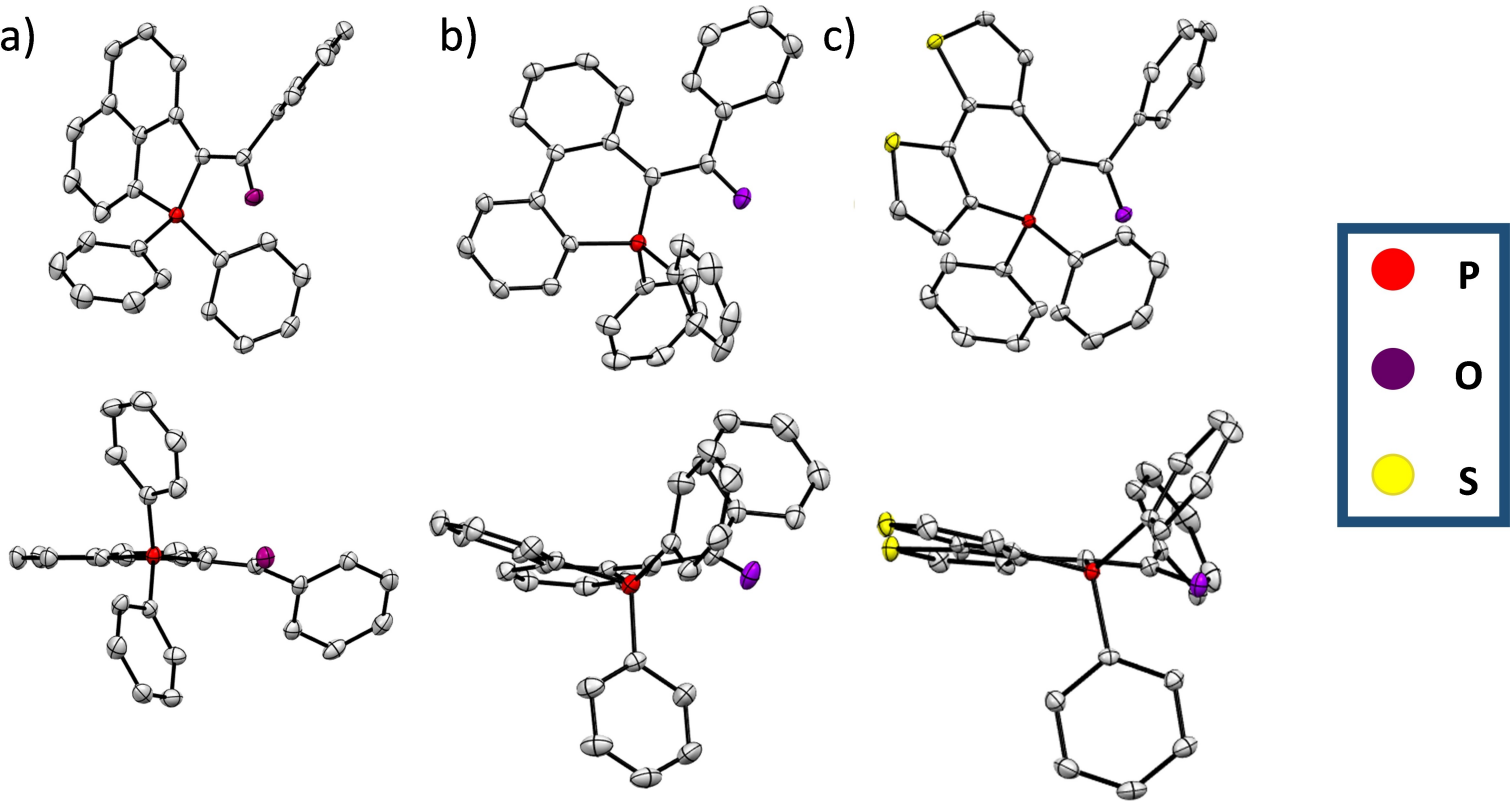
X‐ray structures (top and side view) of a) **5** **a**, b) **7** **a** and c) **9**. Hydrogen atoms are omitted for clarity and thermal ellipsoids are set at 50 % probability.

Additionally, **5** **a**, **5** **b**, **5** **f**, **7** **a**, and **9** were also characterized by X‐ray diffraction (Figure 3).^[32]^ A detailed inspection is performed below for **5** **a** that is representative of the entire family (see Table S1 and S2 for details). The polyaromatic platform is fully planar (maximal deviation from mean plane: 0.09 Å) and the P‐atom adopts a classical tetrahedral shape. The two intramolecular C−P bonds distances are *d*_P‐C4_=1.75 Å and *d*_P‐C1_=1.79 Å, confirming the ylidic nature of P‐C_4_ (Table S1 and S2). Similar P−C distances were reported in **C** (Figure 1) or in related structures.[^15^, ^16^] Interestingly, the C−C between the λ^5^‐phosphole and the carbonyl has a distance of 1.42 Å, intermediate between a C−C and C=C, thus illustrating a conjugation between the polyaromatic P‐ring and the carbonyl. Finally, a short intramolecular O−P distance is observed (*d*=2.90 Å, *d*_VdW_(O−P)=3.32 Å). All these characteristics are constant within the λ^5^‐phosphaacenaphthene family (Table S2). Regarding the 6‐membered P‐rings, the general trends are globally the same (Table S2). The main difference is that non‐trifling distortion from planarity is observed in the polycyclic framework of **7** **a** and **9** (maximal deviation from mean plane: 0.17 Å for **9** and 0.40 Å for **7** **a**). This difference of planarity between **5** **a** and **7** **a** is also obvious from the PCM‐M06‐2X/6‐31G(d) calculations (Figure S39), indicating that this is not an effect induced by crystal packing. Finally, this structural analysis confirmed that all compounds display a ylidic bond and that the carbonyl group is involved in the conjugated framework. This statement is confirmed by the frontier molecular orbitals of Figure 5 in which the carbonyl obviously contributes (in the HOMO for **5** **a**, in both HOMO and LUMO for **7** **a**).

**Figure 4:**
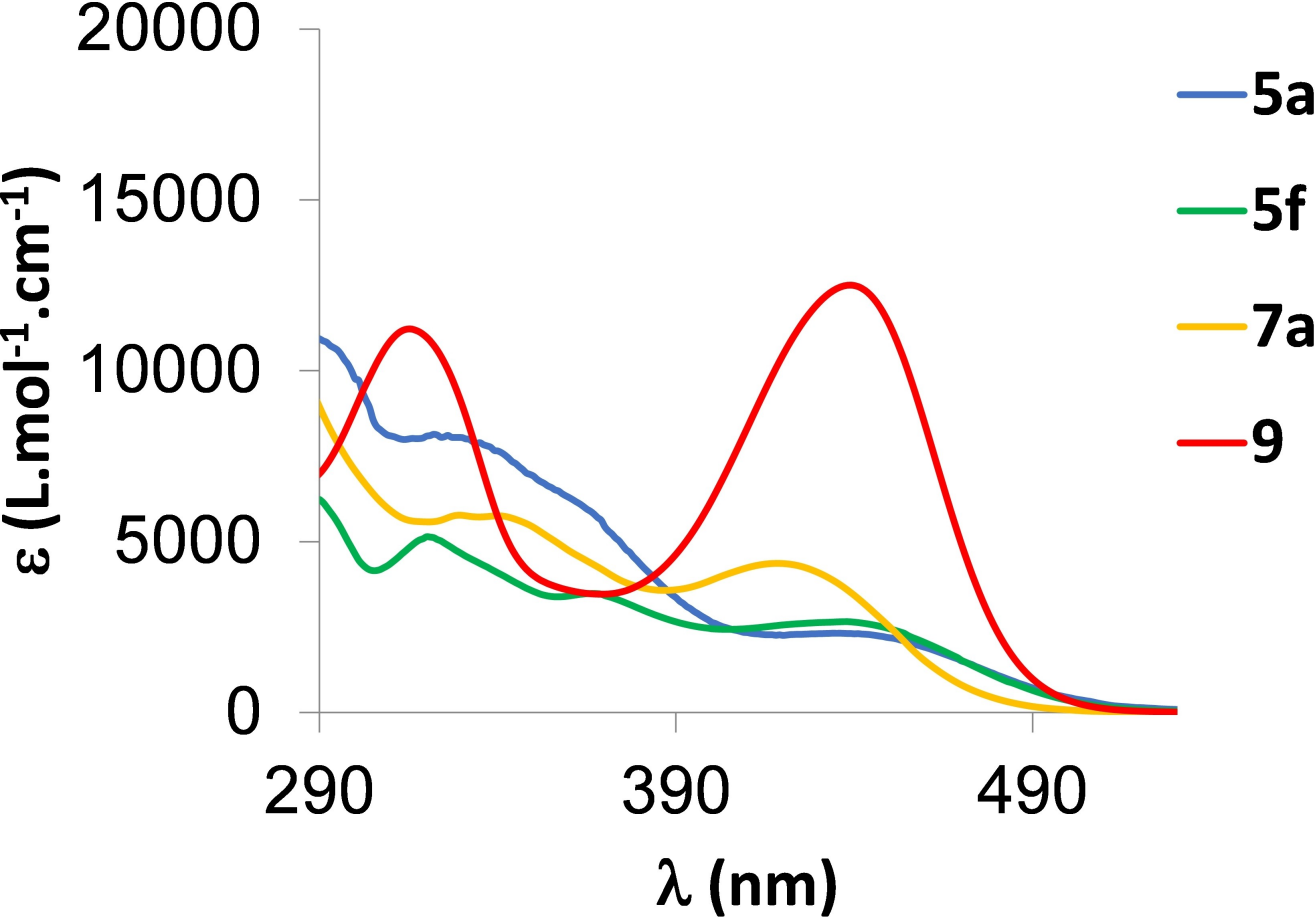
UV/Vis absorption of **5** **a**, **5** **f**, **7** **a** and **9** in dilute DCM solution (10^−5^ M).

The spectroscopic properties of **5**–**9** were investigated in dilute CH_2_Cl_2_ solutions (*c*=5.10^−6^ mol L^−1^, Figure 4 and Table 1). **5** **a** displays absorption bands with moderate extinction coefficients in the UV/Vis range (*λ*_max_=434 nm, *ϵ*=2300 L mol^−1^ cm^−1^). This absorption can be attributed to a π–π* transition with a small charge transfer (CT) character from the carbonyl to the conjugated cycles according to PCM‐TD‐M06‐2X/6‐311+G(2d,p) calculations (see electron density difference plot in Figure S40). Note that this level of theory predicts a vertical absorption at 407 nm (*f*=0.15), slightly blue‐shifted as compared to the measured *λ*_max_, a typical consequence of the neglect of vibronic couplings in the calculations. Interestingly, the most red‐shifted absorption band displays negative solvatochromism (Figure S28). This is consistent with the computed dipole moments of 5.61 and 2.64 D in the ground and excited states of **5** **a**, respectively. The effect of the R‐group attached to the ketone is negligible (Figure S27). Changing from exocyclic Ph to Cy also leads to minor modifications (Figure 4). Changing to 6‐membered P‐rings leads to a gradual hyperchromic shift (**5** **a**–**7** **a**–**9**) of the most red‐shifted transition. This hyperchromic effect is also found in theory that returns a vertical absorption at 372 (*f*=0.24) and 401 nm (*f*=0.30) for **7** **a** and **9**, respectively. Obviously, the trends in both position and intensities closely follow the experimental ones.

**Table 1:** Photophysical and redox data.

	*λ*_abs_^[a]^ [nm]	ϵ^[a]^ [M^−1^ cm^−1^]	*λ*_em(DCM)_^[a]^(ϕ)^[b]^ [nm]	*λ*_em(C5)_^[c]^(ϕ)^[b]^ [nm]	*λ*_emsolid_^[d]^(ϕ)^[b,d]^ [nm]	*E*_ox_^[e]^ [V]	*E*_red_^[e]^ [V]
**5** **a**	434	2300	613 (1 %)	602 (4 %)	549 (25 %)	0.16	–
**5** **b**	435	4700	–	–	–	0.25	−1.52^[f]^
**5** **c**	430	3700	–	–	574 (1 %)	0.29	–
**5** **f**	436	2700	–	–	–	0.01	–
**7** **a**	419	4400	–	–	587 (2 %)	0.23	–
**7** **b**	447	2000	–	–	–	0.39	−1.47^[f]^
**9**	435	12 500	–	–	584 (18 %)	0.21^[f]^	–
**10**	549	27 000	–	616 (51 %)	–	0.28^[f]^	−1.46^[f]^
**11**	485	27 000	578 (<1 %)	529 (32 %)	632 (5 %)	0.21^[f]^	–
**12**	500	27 000	608 (2 %)	554 (48 %)	649 (3 %)	0.04^[f]^	–
**14**	503	15 000	629 (<1 %)	621 (13 %)	643 (12 %)	0.19^[f]^	–
**16** **a**	460	3300	–	591 (8 %)	608 (6 %)	0.19^[f]^	–
**16** **b**	462	5800	–	–	–	0.32^[f]^	−1.48^[f]^

[a] In CH_2_Cl_2_ (10^<M‐>5^M). [b] Measured in calibrated integrated sphere. [c] In pentane (10^−5^ M). [d] Measured in powder [e] In DCM with Bu_4_N^+^PF_6_^−^ (0.2 M) at a scan rate of 200 mV s^−1^. Potentials vs. Fc^+^/Fc. [f] Quasi‐reversible process.

All compounds do not show fluorescence in dilute DCM solution (or only display very weak fluorescence). However, all derivatives featuring R_2_=Ph display solid‐state luminescence in the 550–600 nm area (Figure S29) with moderate QY reaching ≈20 % for **5** **a** and **9** (Table 1).

**Figure 5:**
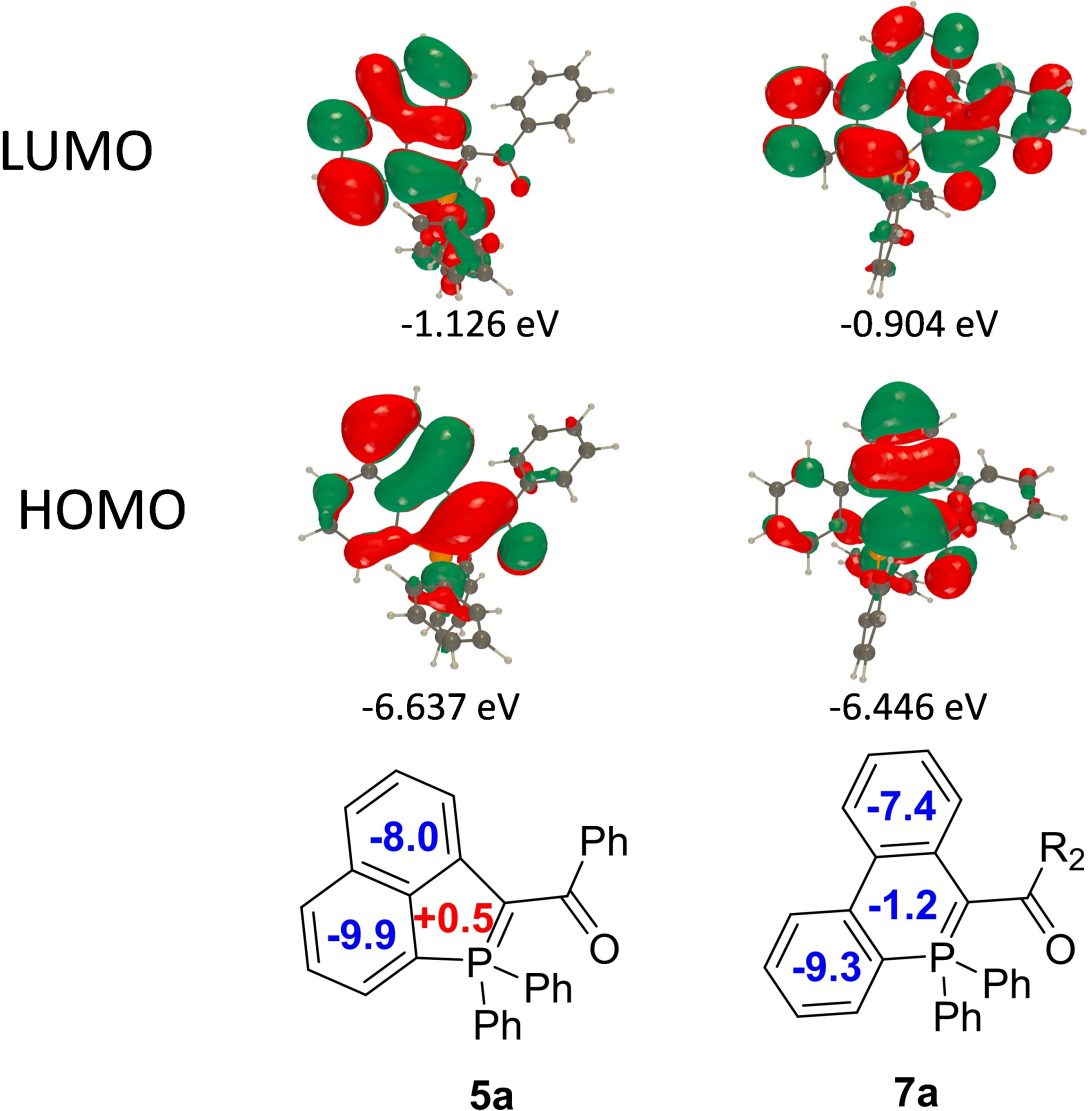
Representation of the frontier MOs as obtained by PCM‐M06‐2X/6‐311+G(2d,p) on PCM‐M06‐2X/6‐31G(d) geometries and NICS(1) aromaticity at the B3LYP/6‐311+G(d,p) level on the same geometries for **5** **a** (left) and **7** **a** (right).

The electrochemical behaviour of **5**–**9** was investigated by cyclic voltammetry (CV) in dichloromethane solution (Figure S34 and S35 and Table 1). **5** **a**–**c** and **5** **f** display irreversible oxidations at low potential (for example *E*_ox_(**5** **a**)=+0.16 V vs. Fc^+^/Fc), in agreement with the strong π‐donating ability of the ylidic fragment, which is confirmed by the HOMO plots showing electronic density on the ylidic fragment (Figure 5 and S39).^[33]^ As expected, insertion of electron‐rich Cy exocyclic groups allows a further decrease of the oxidation potential *E*_ox_(**5** **f**)=+0.01 V vs. Fc^+^/Fc). While no reduction process is observed in **5** **a**, **5** **c** and **5** **f**, the insertion of electron‐withdrawing groups in **5** **b** allows the observation of a quasi‐reversible reduction wave (*E*_red_=−1.52 V vs. Fc^+^/Fc). Increasing the size of the P‐ring from five to six members leads to an increase of the oxidation potential (*E*_ox_(**7** **a**)=+0.23 V vs. Fc^+^/Fc, *E*_ox_(**9**)=+0.21 V vs. Fc^+^/Fc). However, these low values of oxidation potential confirm that all these derivatives remain highly electron‐rich systems.

In addition, the aromaticity of these polycycles has been evaluated using nuclear independent chemical shift (NICS(0)) calculations at the B3LYP/6‐311+G(d,p) level on the PCM‐M06‐2X/6‐31G(d) geometries. (Figures 5 and S47). The P‐cycle is either slightly antiaromatic (e.g., **5** **a**) or slightly aromatic (e.g. **7** **a** and **9**). These values are in the same order of magnitude as those reported for **B** and **C** (Figure 1),[^12^, ^16^] and these P‐rings are therefore better described as non‐aromatic as the absolute NICS(1) (and NICS(0), Figure S47) are very small.[^34^, ^35^, ^36^, ^37^]

All these characterizations confirm that those P‐containing acenaphthylenes and phenanthrenes display optical and redox properties that can be of interest in the context of plastic electronics or photonics. To go further with these systems, it is highly desirable to perform molecular engineering in order to optimize these properties or obtain new ones.

**Scheme 4:**
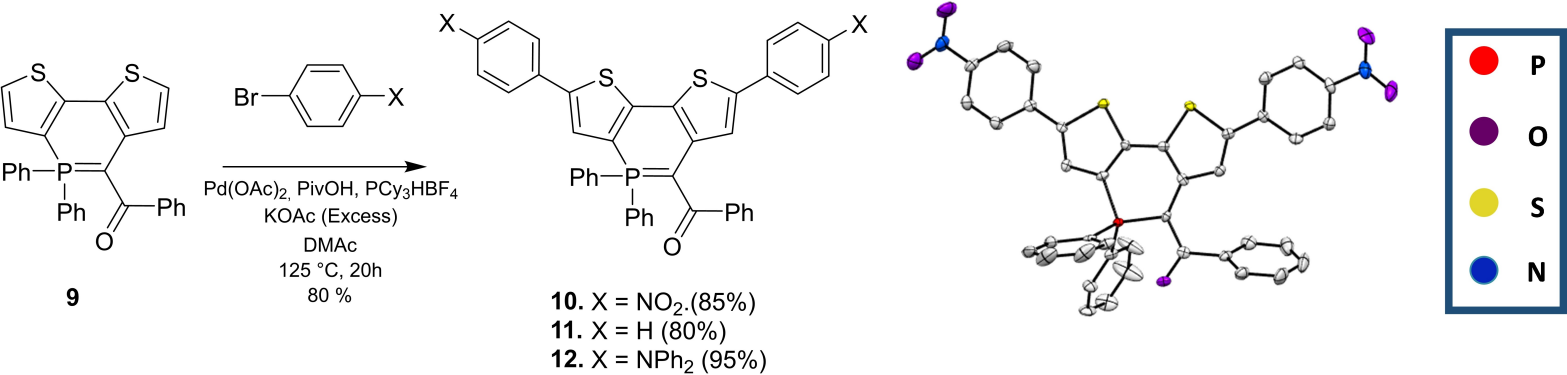
Synthesis of **10**–**12** and X‐ray structure of **10**. Hydrogen atoms are omitted for clarity and thermal ellipsoids are set at 50 % probability.

Despite the presence of the ylidic bond, **7**–**9** show sufficient thermal stability to be engaged in post‐functionalization reactions. We thus decided to test the direct arylation, which offers a straightforward diversification method directly on the C−H bonds.[^38^, ^39^] In this context, thiophene‐containing **9** appeared as an ideal platform. Using 5 % Pd(OAc)_2_ as catalyst, KOAc as base, in DMAc at 125 °C, allowed derivatives **10**–**12** featuring electron‐withdrawing or electron donating groups to be prepared in excellent yields (Scheme 4). In addition, during the reaction with bromonitrobenzene, we could isolate the monoarylation product revealing that the first arylation occurs on the thiophene fused to the 2–3 position of the P‐ring (compound **10′**, see Figure S25). **10** was additionally characterized by X‐ray diffraction (Scheme 4) and the main structural characteristics described earlier for **9** remain valid.

**Figure 6:**
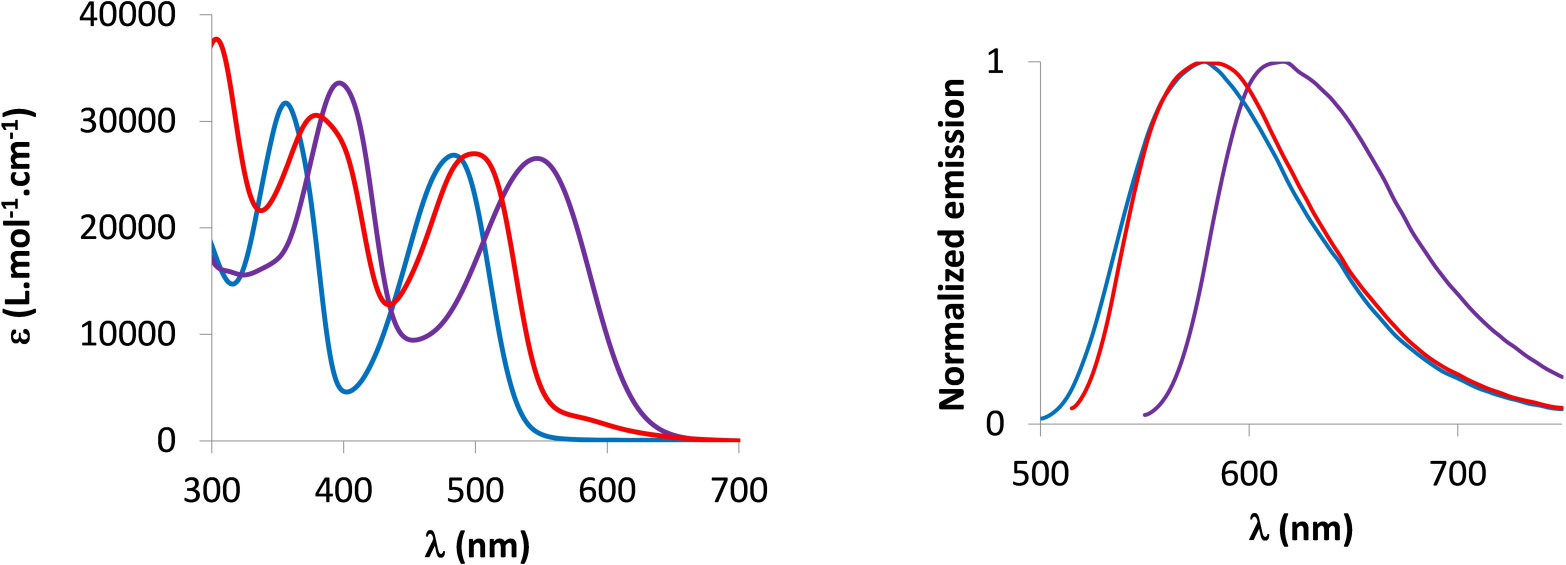
UV/Vis absorption in DCM (5×10^−5^ M) (left) and emission in pentane (5×10^−5^ M) (right) of **10** (purple), **11** (blue), and **12** (red).

**Figure 7:**
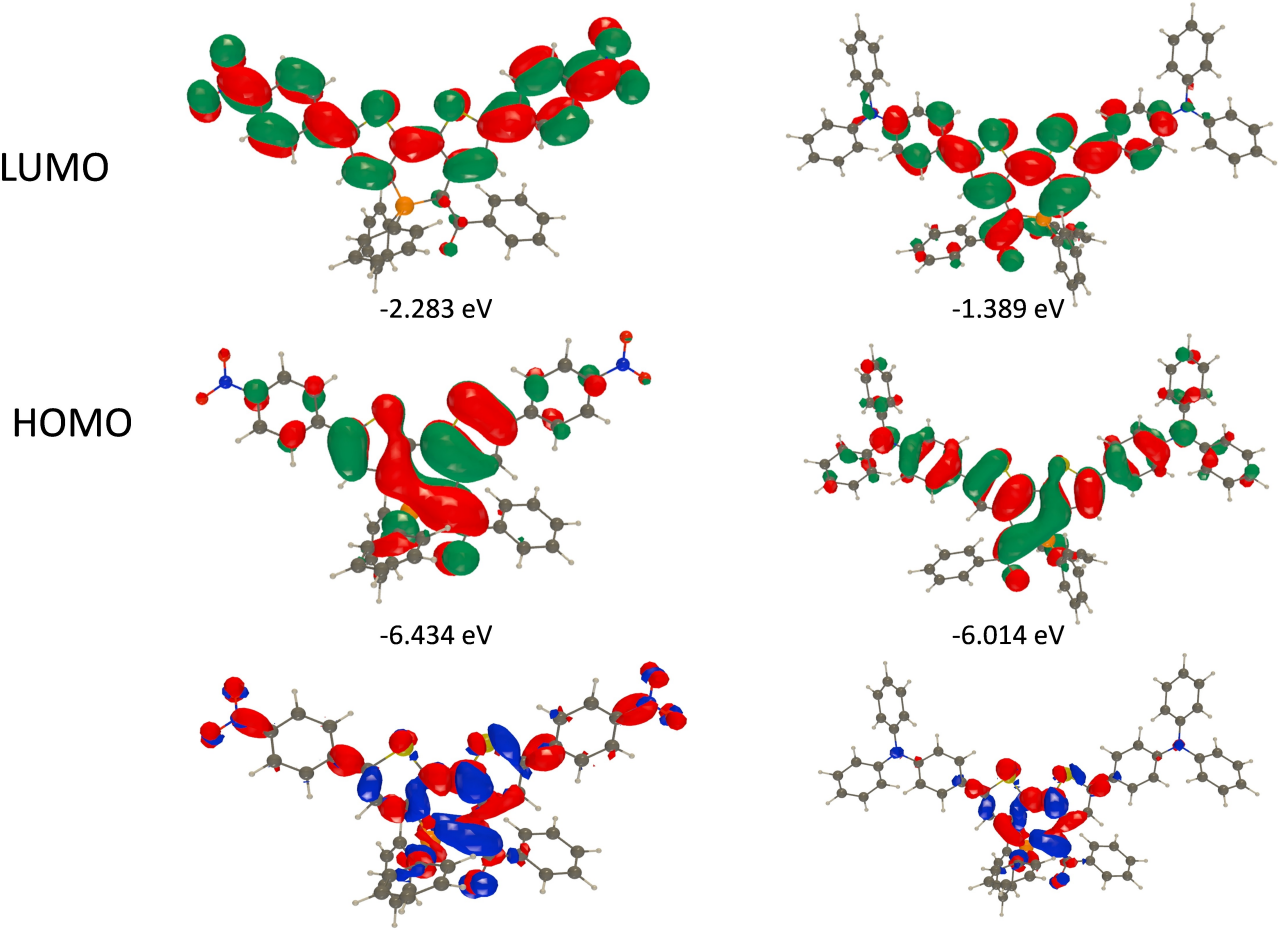
Representation of the frontier MOs (top, middle) and density difference plots (bottom) of **10** (left) and **12** (right). The blue and red lobes correspond to regions of decrease and increase of electron density upon excitation (absorption), respectively (contour threshold: 1×10^−3^). All data at the PCM‐M06‐2X/6‐311+G(2d,p)//PCM‐M06‐2X/6‐31G(d) level.

The extension of the π‐system and the insertion of an electron‐rich/‐poor substituent leads to bathochromic and hyperchromic shifts compared to the unsubstituted λ^5^‐dithienophosphinine **9** (Table 1). **10**–**12** display absorptions covering the visible range from 400 to 550 nm (*ϵ*_max_≈27000 L mol^−1^ cm^−1^). As anticipated by the electro‐donating character of the ylidyl ring, the insertion of electron‐poor nitroaryl groups in **10** leads to a red‐shifted absorption (*λ*_abs_=549 nm), whereas donor groups induce milder displacements. A similar trend is observed with the luminescence of the compounds (Table 1 and Figure 6). While the absorption wavelengths are only weakly affected by the polarity of the solvent (Figure S30), all compounds display higher luminescence quantum yields in apolar solvent (pentane) with ϕ up to 50 % for **10** and **12**. Interestingly, **11** and **12** also display solid‐state emission in the red part of the visible range. TD‐DFT reproduces these trends with bright vertical absorptions at 491, 439, and 451 nm for **10**, **11**, and **12**, respectively. Fluorescence wavelengths follow the same ordering (Table S3). We provide HOMO–LUMO plots and electron density difference plots for these three compounds in Figure 7 and Figure S42. For **10**, there is an obvious CT from the core of the dye to the nitro group, confirming the electro‐donating character of the ylidyl ring. In contrast, in both **11** and **12**, the side groups do not play any major role in the transition, explaining why these two compounds present similar spectral signatures.

**Figure 8:**
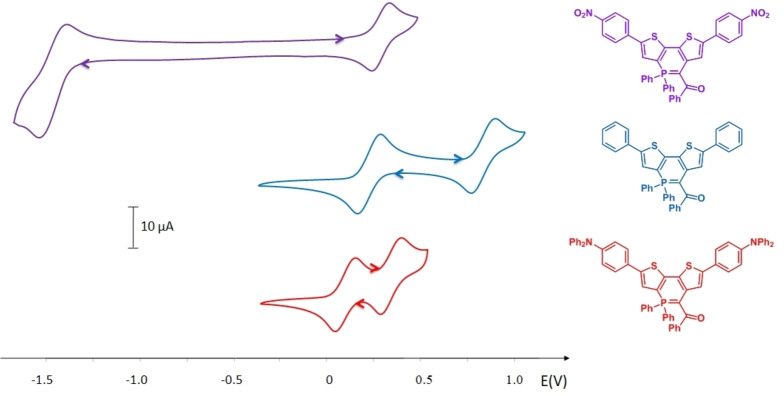
Cyclic voltammograms of **10**–**12** (*c*=10^−3^ M) recorded in DCM (Bu_4_NPF_6_ (0.2 M), 200 mV s^−1^, potentials vs. Fc^+^/Fc).

The CV reveal that insertion of nitroaryl allows the observation of a quasi‐reversible reduction (*E*_red_(**10**)=−1.46 V vs. Fc^+^/Fc, Figure 8 and Table 1) in addition to the oxidation at low potential. Indeed, density is observed on the nitrophenyl part in the LUMO of **10** (Figure 7). Thus, **10** appears to be an interesting derivative with amphoteric redox character and a low band gap. The presence of electron‐rich substituents in addition to the ylidic ring in **11** and **12** (*E* ${}_{\mathrm{ox}{}_{1}}$ ) leads to the presence of multiple oxidation processes (*E* ${}_{\mathrm{ox}{}_{2}}$ (**11**)=+0.81 V vs. Fc^+^/Fc; *E* ${}_{\mathrm{ox}{}_{2}}$ (**12**)=+0.30 V vs. Fc^+^/Fc, see Figure 8). However, the most electron‐rich part of the compound remains the cyclic ylide. Hence, electronic density in the HOMO of **11** and **12** is mainly on the central ylidic ring (Figure 7). Finally, it is worth mentioning that the post‐functionalization does not modify the aromaticity of the P‐cycles, which remain non‐aromatic in the whole series (Figure S48, S49). These results illustrate the easy tuning of the HOMO–LUMO energies and thus the optical and redox properties, making these derivatives ideal synthons to build a new generation of π‐conjugated organophosphorus materials.

In order to further explore the photonic properties of these new dyes, the third‐order non‐linear optical (NLO) properties of **12** were evaluated by the two‐photon‐absorption‐induced fluorescence method in hexane (see Supporting Information). We show that **12** displays two‐photon absorption (2PA) in the near infrared with *σ*=80 GM±15 % at 810 nm (see Figure S32 and S33). The main 2PA band is thus blue‐shifted compared to 2.*λ*_max_ (*λ*_max_=500 nm, Table 1). Theoretical calculations confirm that the S_0_–S_1_ transition has a very low *σ* of 6 GM, whereas the S_0_–S_2_ transition delivers a much larger two‐photon response (96 GM according to QR‐TD‐CAM‐B3LYP/6‐31G, Table S4). Hence, this band indeed shows a very strong CT nature, in contrast to the lowest one (Figure S46). Such results illustrate that an optimized version of these dyes can be attractive for various photonic applications either in the field of materials science (optical power limiting) or medicine (bio‐imaging, phototherapy).^[40]^

**Scheme 5:**
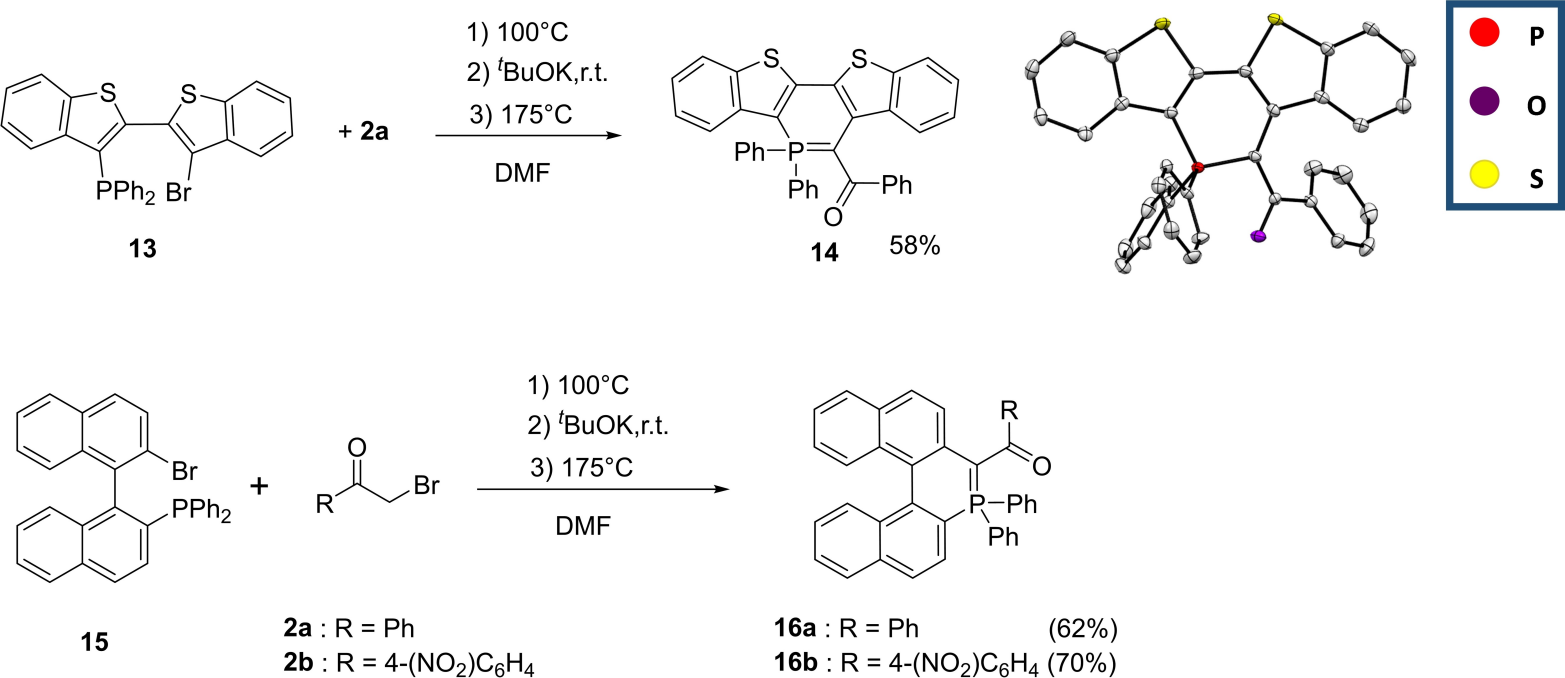
Synthesis of phosphaacene **14** and phosphahelicene **16** **a**, **b** and X‐ray structure of **14**. Hydrogen atoms are omitted for clarity and thermal ellipsoids are set at 50 % probability.

Polyaromatic systems, either linearly or helically arranged, nowadays play a key role in optoelectronic applications. It is thus of great interest to check if our synthetic approach allows the introduction of such scaffolds. The method appeared fully compatible for the efficient preparation of linear heteroacene **14** and of hetero[5]helicenes **16** **a**, **b** (Scheme 5).

**Figure 9:**
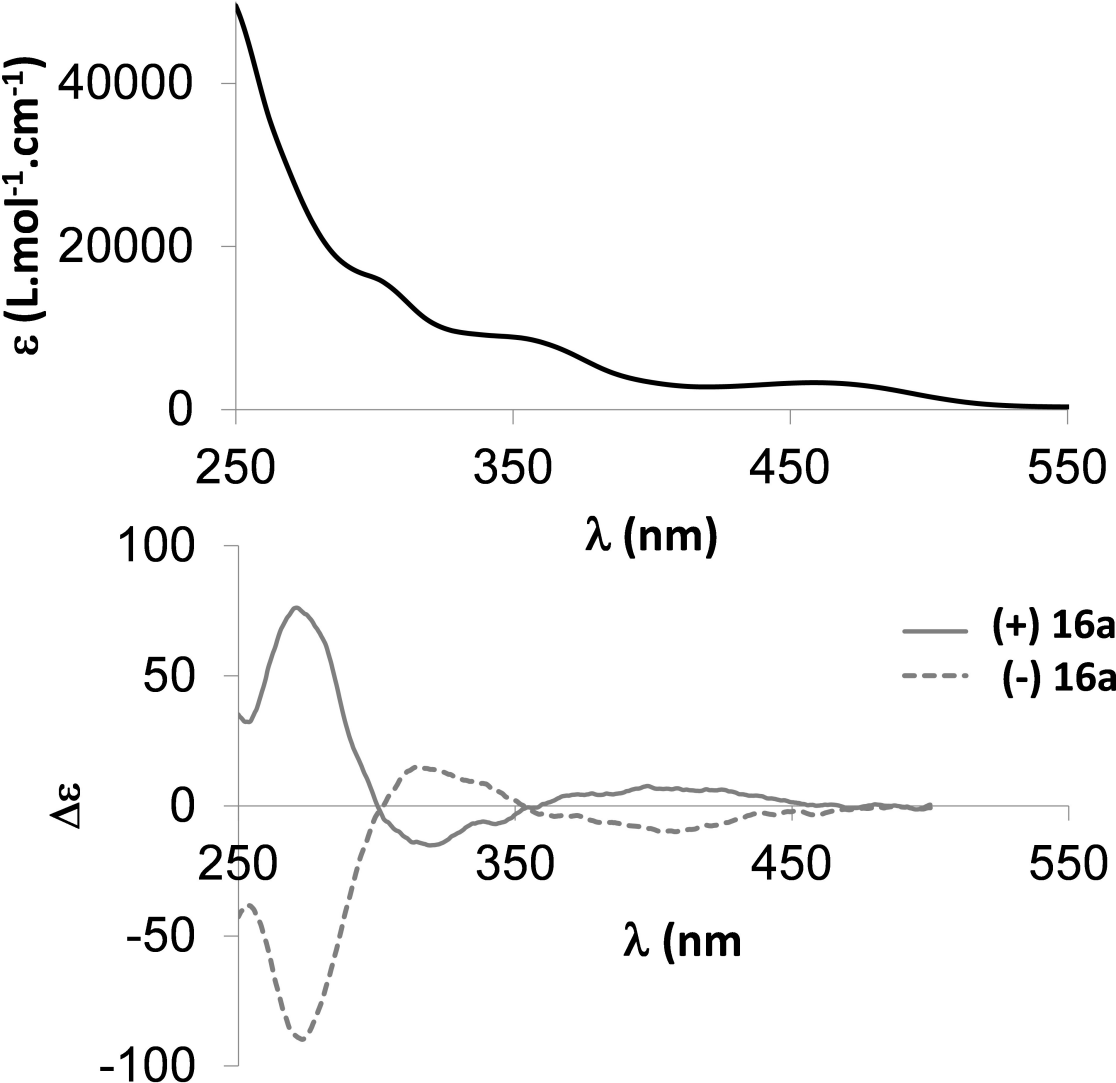
UV/Vis absorption of **16** **a** and ECD spectra of (+)‐**16** **a** (plain), (−)‐**16** **a** (dotted) in DCM (10^−5^ M).

As expected, the π‐extension of **14** compared to **9** leads to a red‐shift of the absorption/emission without significant modification of the oxidation potential (Table 1 and Figure S31, S37). In these polyaromatic systems, the 6‐membered P‐ring remains mostly non‐aromatic (Figure S50, S51). More interestingly, **16** **a**, **b** are the first examples of phosphahelicenes featuring an ylidic bond.^[41]^ Enantiopure samples (with ee higher than 99 %) of **16** **a** were obtained via HPLC over a chiral stationary phase (see Supporting Information). The enantiomerization barrier was determined to be Δ*G* ^≠^=132.2 kJ mol^−1^ at 132 °C in chlorobenzene, (Figure S38), 30 kJ mol^−1^ higher than for [5]‐carbohelicene,^[42]^ showing its high enantiomeric stability. Such behaviour was also previously observed with the 7‐membered P‐ring based [5]helicenoïds, highlighting the pertinence of tuning helicenoïds with P‐rings.^[43]^ Such configurational stability allows the study of the chiroptical properties of the λ^5^‐phosphahelicene. **16** **a** displays high specific optical rotations [(+)**16** **a**: α^23^_D_=+346±5%; *c*=10^−2^ M, DCM]. Electronic circular dichroism (ECD) was recorded in dilute DCM solutions. (+)**16** **a** and (−)**16** **a** displayed ECD with the expected mirror‐image relationship (Figure 9). (+)**16** **a** displays a strong positive ECD band (Δ*ϵ*=75 at 272 nm), a negative band (Δ*ϵ*=−15 at 320 nm) and positive band (Δ*ϵ*=8 at 398 nm). The spectra were nicely fit by PCM‐TD‐B3LYP/6‐311+G(2d,p) calculations (Figure S45), which provides peaks with Δ*ϵ* of +104 at 291 nm, −43 at 345 nm, and +8 at 380 nm. This match enables the absolute configuration to be assigned as *P*‐(+)/*M*‐(−). In conclusion, these data confirm that λ^5^‐phosphahelicene **16** **a** exhibits the important chiroptical properties that make helicene unique and attractive for optoelectronic applications.

## Conclusion

In conclusion, a straightforward one‐pot synthesis of 5‐ or 6‐membered P‐heterocycles featuring an internal ylidic bond (P‐containing acenaphthylenes and phenanthrenes) is presented (**5**–**9**). These π‐conjugated systems are easily obtained through intramolecular cyclization of ylides, generated from readily available phosphines. Thanks to their chemical stability, these derivatives are easily post‐functionalized through Pd‐catalysed direct‐arylation allowing the tuning of their absorption/emission across the entire visible range as well as their redox properties. Functional dyes with nonlinear optical properties can thus be prepared (**10**–**12**). In addition, the synthetic approach is also compatible with the preparation of polyaromatic derivatives, either linear (**14**) or helical (**16** **a**–**b**). The latter approach allowed the preparation of configurationally stable phospha[5]helicenes with chiroptical properties. In the future, this novel synthetic approach might be attractive for preparing more complex π‐systems (higher phosphahelicenes, phosphananographenes etc) or functional dyes with applications in the field of organic electronics or photonics.

## Conflict of interest

The authors declare no conflict of interest.

1

## Supporting information

As a service to our authors and readers, this journal provides supporting information supplied by the authors. Such materials are peer reviewed and may be re‐organized for online delivery, but are not copy‐edited or typeset. Technical support issues arising from supporting information (other than missing files) should be addressed to the authors.

Supporting Information

Supporting Information

Supporting Information

Supporting Information

Supporting Information

Supporting Information

Supporting Information

Supporting Information

Supporting Information

## Data Availability

The data that support the findings of this study are available in the Supporting Information of this article.
