# Supplementary material for: Straightforward Access to Multifunctional π‐Conjugated P‐Heterocycles Featuring an Internal Ylidic Bond
Source: Angew Chem Int Ed Engl. 2022 Jun 23;61(31):e202205548. doi: 10.1002/anie.202205548 (PMC9400969; doi:10.1002/anie.202205548)
Supplement: Supplementary file 9 — Supporting Information [file ANIE-61-0-s008.pdf]

Angewandte Chemie
Eine Zeitschrift der Gesellschaft Deutscher Chemiker

Supporting Information

Straightforward Access to Multifunctional π-Conjugated P-Heterocycles Featuring an Internal Ylidic Bond

T. Delouche, E. Caytan, M. Cordier, T. Roisnel, G. Taupier, Y. Molard, N. Vanthuyne, B. Le Guennic, M. Hissler, D. Jacquemin, P.-A. Bouit**

Supporting information

Table of Content

**Experimental section**	**page S2**
**NMR spectra**	**page S10**
**Crystallographic data and structure refinement parameters**	**Page S37**
**Optical properties**	**page S46**
**Redox Properties**	**page S50**
**Chiral HPLC**	**page S52**
**Theoretical chemistry**	**page S56**

Experimental section.

All experiments were performed under argon atmosphere using standard Schlenk techniques. Commercially available reagents were used as received without further purification. Solvents were freshly purified using MBRAUN SPS-800 drying columns. Separations were performed by gravity column chromatography on basic alumina (Aldrich, Type 5016A, 150 mesh, 58 Å) or silica gel (Merck Geduran 60, 0.063-0.200 mm). ^{1}H, ^{13} C, and ^{31}P NMR spectra were recorded on Bruker AV III 300 and 400 MHz NMR spectrometers equipped with BBO or BBFO probe heads. Assignment of proton and carbon atoms is based on COSY, NOESY, edited-HSQC and HMBC experiments. Special ^{31}P decoupled experiments ($\{^{31}P\}^{1}H$, $\{^{31}P-^{1}H\}^{13}C$, $\{^{31}P\}$-HSQC, HMBC, COSY, NOESY) were performed on a Bruker Av III HD 500 MHz fitted with a triple inverse probehead ($^{1}H-^{31}P-X$).These 31P decoupled experiments have been performed using the PRISM core facility (Biogenouest©, UMS Biosit, Université de Rennes 1- Campus de Villejean- 35043 RENNES Cedex, FRANCE). ^{1}H and ^{13}C NMR chemical shifts were reported in parts per million (ppm) using residual solvent signal as reference. High-resolution mass spectra were obtained on a Varian MAT 311 or ZabSpec TOF Micromass instrument at CRMPO (Scanmat, UMS 2001). UV-Visible spectra were recorded rt on a VARIAN Cary 5000 spectrophotometer. The UV-Vis emission and excitation spectra measurements were recorded on a FL 920 Edimburgh Instrument equipped with a Hamamatsu R5509-73 photomultiplier for the NIR domain (300-1700 nm) and corrected for the response of the photomultiplier. The absolute quantum yields were measured with a C9920-03 Hamamatsu. The electrochemical studies were carried out under argon using an Eco Chemie Autolab PGSTAT 30 potentiostat for cyclic voltammetry with the three-electrode configuration: the working electrode was a platinum disk, the reference electrode was a saturated calomel electrode and the counter-electrode a platinum wire. All potential were internally referenced to the ferrocene/ferrocenium couple. For the measurements, concentrations of 10^{-3} M of the electroactive species were used in freshly distilled and degassed DCM and 0.2 M tetrabutylammoniumhexafluorophosphate. **1a**, **6a** and **15** were prepared according to the literature.[1]

[1] a) J. Beckmann, T. G. Do, S. Grabowsky, E. Hupf, E. Lork and S. Mebs, *Z. anorg. allg. Chem.*, 2013, **639**, 2233–2249; b) H. Brunner and M. Janura, *Synthesis*, 1998, **1998**, 45–55; c) K. J. Brown, M. S. Berry, K. C. Waterman, D. Lingenfelter and J. R. Murdoch, *J. Am. Chem. Soc.*, 1984, **106**, 4717–4723

1b: ***(General method A)*** 1,8-dibromonaphtalene (1 g, 3,5 mmol, 1 eq) is dissolved in 50 mL of dry Et_2O. The solution is cooled down to -80°C and *t*-BuLi (1 eq) is added dropwise and the solution is stirred for 30 mn at -80°C and 30 mn at rt. The solution is again cooled down to -80°C and Cy_2PCl (0,7 mL, 3,5 mmol, 1 eq) is added dropwise and the mixture is stirred at RT overnight. Then the solution is quenched with water, the solution is extracted with water and evaporated. The crude mixture was purified by silica gel chromatography (C_7/DCM: 9/1) to afford **1b** as a white powder (720 mg, η = 57 %). ^1H NMR (300 MHz, CD_2Cl_2) δ 7.87 (dd, J = 7.4, 1.3 Hz, 1H, H_2), 7.85 – 7.79 (m, 3H, $H_{4,6,8}$), 7.47 (t, J = 7.6 Hz, 1H, H_7), 7.25 (t, J = 7.7 Hz, 1H, H_3), 2.10 – 1.94 (m, 4H, H_{Cy}), 1.84 – 1.73 (m, 2H, H_{Cy}), 1.68 – 1.54 (m, 6H, H_{Cy}), 1.38 – 1.09 (m, 10H, H_{Cy}). ^{13}C NMR (75 MHz, CD_2Cl_2) δ 137.0 (d, J_{C-P} = 3.8 Hz, C_5), 136.2 (d, J_{C-P} = 18.7 Hz, C_{10}), 135.4 (d, J_{C-P} = 38.4 Hz, C_1), 135.0 (d, J_{C-P} = 1.8 Hz, C_2), 134.7 (C_8), 130.5 (C_6), 130.0 (d, J_{C-P} = 1.6 Hz, C_4), 126.0 (C_3), 125.5 (C_7), 120.9 (d, J_{C-P} = 3.1 Hz, C_9), 37.4 (d, J_{C-P} = 20.0 Hz, C_{Cy}), 31.0 (C_{Cy}), 30.8 (C_{Cy}), 30.4 (C_{Cy}), 30.2 (C_{Cy}), 27.9 (d, J = 10.5 Hz, C_{Cy}), 27.7 (d, J_{C-P} = 11.0 Hz, C_{Cy}), 26.9 (d, J = 1.3 Hz, C_{Cy}). ^{31}P NMR (121 MHz, CD_2Cl_2) δ -8.4. HRMS (ESI, CH_3OH/CH_2Cl_2 : 9/1) $[M+H]^+$ ($C_{22}H_{29}BrP$) : m/z Theoretical : 403.1185, m/z Found : 403.1185.

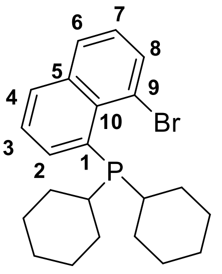

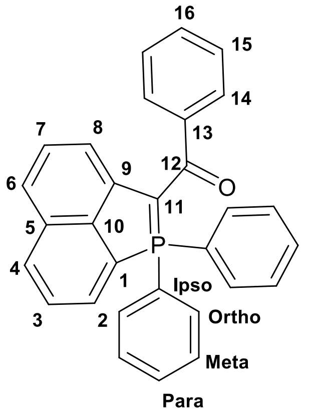

5a: ***(General method B)*** **1a** (0.391 mg, 1.00 mmol, 1 eq) and 2-Bromoacetophenone (219 mg, 1.10 mmol, 1.1 eq) were dissolved in 5 mL of toluene. The mixture was heated at 90 °C for 3 h. Then, ^{t}BuOK (168 mg, 1.50 mmol, 1.5 eq) was added at room temperature (r.t.) and the mixture stirred for 30 min allowing the obtention of the phosphonium ylides. The reaction was then heated to reflux for 4 h. After evaporation of the solvent, the crude product was purified through silica gel column chromatography (DCM/Acetone: 9/1). A yellow-orange powder was obtained. (244 mg, η = 57 %). ^1H NMR (400 MHz, CD_2Cl_2) δ 8.03 (d, J = 8.1 Hz, 1H, H_4), 7.95 (dd, J = 13.7, 7.2 Hz, 4H, H_{ortho}), 7.81 (dd, J = 8.8, 7.1 Hz, 1H, H_2), 7.72 – 7.62 (m, 5H, $H_{3,15,para}$), 7.60 – 7.53 (m, 4H, H_{meta}), 7.51 – 7.46 (m, 3H, $H_{14,16}$), 7.28 – 7.20 (m, 2H, $H_{6,7}$), 6.68 (d, J = 6.9, 1.3 Hz, 1H, H_8). ^{13}C NMR (101 MHz, CD_2Cl_2) δ 185.7 (d, J_{C-P} = 8.1 Hz, C_{12}), 143.4 (d, J_{C-P} = 12.9 Hz, Cq), 140.1 (d, J_{C-P} = 25.7 Hz, Cq), 135.8 (d, J_{C-P} = 23.7 Hz, Cq), 133.6 (d, J_{C-P} = 11.4 Hz, C_{ortho}), 132.9 (d, J_{C-P} = 3.1 Hz, C_{para}), 132.8 (d, J_{C-P} = 9.7 Hz, Cq), 132.1 (d, J_{C-P} = 2.7 Hz, C_4), 129.6 (d, J_{C-P} = 2.2 Hz, C_7), 129.3 (d, J_{C-P} = 13.2 Hz, C_{meta}), 129.3 (C_{16}), 128.6 (C_{14}), 128.4 (d, J_{C-P} = 6.4 Hz, C_2), 127.7 (s, C_{15}), 127.0 (d, J_{C-P} = 11.2 Hz, C_3), 125.9 (d, J_{C-P} = 91.3 Hz, C_{ipso}), 124.1 (d, J_{C-P} = 87.5 Hz, C_1), 117.6 (s, C_6), 115.5 (d, J_{C-P} = 14.1 Hz, C_8), 77.8 (d, J_{C-P} = 107.2 Hz, C_{11}). ^{31}P NMR (162 MHz, CD_2Cl_2) δ +22.8. HRMS (ESI, CH_3OH/CH_2Cl_2 : 9/1) $[M+Na]^+$ ($C_{30}H_{21}ONaP$) : m/z Theoretical : 451.1222, m/z Found : 451.1222.

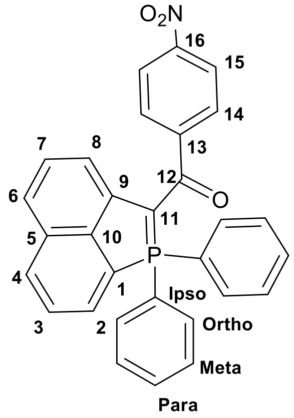

5b: **General method B** was used with **1a** (391 mg, 1.00 mmol, 1 eq), 4-Nitrophenacyl bromide (268 mg, 1.10 mmol, 1.1 eq), ^tBuOK (168 mg, 1.50 mmol, 1.5 eq) in 5 mL of toluene. The product was purified through silica gel column chromatography (DCM/Acetone: 9/1). A dark red powder was obtained (290 mg, η = 70%). ^1H NMR (400 MHz, CD_2Cl_2) δ 8.30 (d, J = 8.6 Hz, 2H, H_{15}), 8.03 (d, J = 8.2 Hz, 1H, H_2), 7.96 – 7.87 (m, 4H, H_{ortho}), 7.85 – 7.78 (m, 3H, $H_{4,14}$), 7.70 – 7.60 (m, 3H, $H_{3,para}$), 7.58 – 7.51 (m, 4H, H_{meta}), 7.28 (d, J = 8.2 Hz, 1H, H_6), 7.23 – 7.17 (m, 1H, H_7), 6.54 (d, J = 7.3 Hz, 1H, H_8). ^{13}C NMR (101 MHz, CD_2Cl_2) δ 182.6 (d, J_{C-P} = 8.6 Hz, C_{12}), 149.8 (d, J_{C-P} = 13.4 Hz, Cq), 148.4 (s, Cq), 139.4 (d, J_{C-P} = 25.0 Hz, Cq), 135.8 (d, J = 23.4 Hz, Cq), 133.6 (d, J_{C-P} = 11.5 Hz, C_{ortho}), 133.1 (d, J_{C-P} = 3.2 Hz, C_{para}), 132.8 (d, J_{C-P} = 9.7 Hz, C_2), 132.4 (d, J_{C-P} = 2.8 Hz, Cq), 129.5 (s, C_7), 129.4 (d, J_{C-P} = 13.3 Hz, C_{meta}), 128.8 (s, C_{14}), 128.7 (d, J_{C-P} = 6.5 Hz, C_4), 127.3 (d, J_{C-P} = 11.4 Hz, C_3), 125.2 (d, J_{C-P} = 91.2 Hz, C_{ipso}), 124.2 (s, C_{15}), 123.6 (d, J_{C-P} = 88.3 Hz, C_1), 118.4 (s, C_6), 115.5 (d, J_{C-P} = 13.7 Hz, C_8), 78.9 (d, J_{C-P} = 107.8 Hz, C_{11}). ^{31}P NMR (162 MHz, CD_2Cl_2) δ +23.6. HRMS (ESI, CH_3OH/CH_2Cl_2 : 9/1) $[M+Na]^+$ ($C_{30}H_{20}NO_3NaP$) : m/z Theoretical : 496.1073, m/z Found : 496.1069.

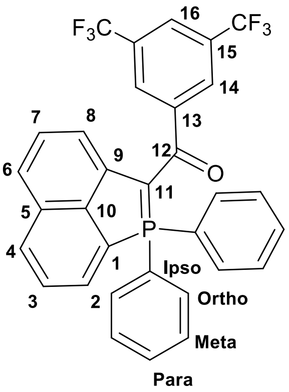

5c: **General method B** was used with **1a** (391 mg, 1.00 mmol, 1 eq), 3’,5’-Bis(trifluoromethyl)-2-bromoacetophenone (369 mg, 1.10 mmol, 1.1 eq), ^tBuOK (168 mg, 1.50 mmol, 1.5 eq) in 5 mL of toluene. The product was purified through silica gel column chromatography (DCM/Acetone: 98/2). A yellow powder was obtained (337 mg, η = 60 %). ^1H NMR (400 MHz, CD_2Cl_2) δ 8.22 (d, J = 1.7 Hz, 2H, H_{14}), 8.07 – 8.02 (m, 1H, H_{16}), 8.01 – 7.98 (m, 1H, H_2), 7.92 (ddd, J = 13.8, 8.4, 1.4 Hz, 4H, H_{ortho}), 7.85 – 7.80 (m, 1H, , H_4), 7.70 – 7.60 (m, 3H, $H_{3,para}$), 7.58 – 7.52 (m, 4H, H_{meta}), 7.33 – 7.29 (m, 1H, H_6), 7.27 – 7.20 (m, 1H, H_7), 6.61 (d, J = 7.3 Hz, 1H, H_8). ^{13}C NMR (101 MHz, CD_2Cl_2) δ 181.1 (d, J_{C-P} = 9.0 Hz, C_{12}), 145.3 (d, J_{C-P} = 13.8 Hz, Cq), 139.3 (d, J_{C-P} = 24.8 Hz, Cq), 136.0 (d, J_{C-P} = 23.4 Hz, Cq), 133.7 (d, J_{C-P} = 11.5 Hz, C_{ortho}), 133.2 (d, J_{C-P} = 3.1 Hz, C_{para}), 133.0 (d, J_{C-P} = 9.7 Hz, Cq), 132.5 (d, J_{C-P} = 2.7 Hz, C_{16}), 131.8 (d, J_{C-P} = 33.3 Hz, Cq), 129.5 (d, J_{C-P} = 13.3 Hz, C_{meta}), 129.5 (d, J_{C-P} = 2.3 Hz, C_7), 128.8 (d, J_{C-P} = 3.6 Hz, C_{14}), 128.7 (d, J_{C-P} = 6.6 Hz, C_4), 127.4 (d, J_{C-P} = 11.3 Hz, C_3), 125.3 (d, J_{C-P} = 91.2 Hz, C_1), 124.8 (d, J_{C-P} = 125.9 Hz, C_{ipso}), 123.0 (d, J_{C-P} = 58.3 Hz, C_2), 123.1 – 122.9 (m, C_{-CF3}), 118.7 (s, C_6), 115.4 (d, J_{C-P} = 13.8 Hz, C_8), 78.9 (d, J_{C-P} = 108.4 Hz, C_{11}).^{19}F NMR (376 MHz, CD_2Cl_2) δ -63.1. ^{31}P NMR (162 MHz, CD_2Cl_2) δ +24.2. HRMS (ESI, CH_3OH/CH_2Cl_2 : 9/1) $[M+H]^+$ ($C_{32}H_{20}F_6OP$) : m/z Theoretical : 565.1151, m/z Found : 565.1153.

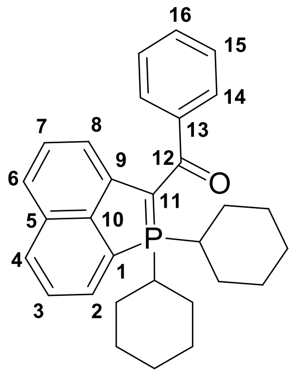

__5f__: **General method B** was used with **1b** (0.350 mg, 0.87 mmol, 1 eq), 2-Bromoacetophenone (191 mg, 0.96 mmol, 1.1 eq), ^{t}BuOK (147 mg, 1.50 mmol, 1.5 eq) in 5 mL of toluene. After evaporation of the solvent, the crude product was purified through silica gel column chromatography (DCM/AcOEt : 95/5). A yellow-orange powder was obtained (180 mg, η = 47 %).^{1}H NMR (400 MHz, CD_2Cl_2) δ 7.93 (d, J = 8.3 Hz, 1H, H_4), 7.72 (t, J = 7.2 Hz, 1H, H_3), 7.63 – 7.53 (m, 3H, $H_{2,15}$), 7.50 – 7.41 (m, 3H, $H_{14,16}$), 7.16 – 7.03 (m, 2H, $H_{6,7}$), 6.38 (d, 1H, H_8), 2.94 – 2.80 (m, 2H, H_{Cy}), 2.17 – 2.07 (m, 2H, H_{Cy}), 1.88 – 1.76 (m, 6H, H_{Cy}), 1.74 – 1.66 (m, 2H, H_{Cy}), 1.63 – 1.50 (m, 2H, H_{Cy}), 1.45 – 1.30 (m, 6H, H_{Cy}), 1.25 – 1.09 (m, 2H, H_{Cy}). ^{13}C NMR (101 MHz, CD_2Cl_2) δ 185.5 (d, J_{C-P} = 12.8 Hz, C_{12}), 141.9 (d, J_{C-P} = 20.0 Hz, Cq), 137.2 (d, J_{C-P} = 19.0 Hz, Cq), 134.0 (s, Cq), 132.5 (d, J_{C-P} = 8.6 Hz, Cq), 131.6 (d, J_{C-P} = 2.6 Hz, C_4), 129.2 (d, J_{C-P} = 2.1 Hz, C_6), 128.9 (s, C_{16}), 128.7 (s, C_{14}), 128.0 (d, J_{C-P} = 5.8 Hz, C_3), 127.5 (s, C_{15}), 126.4 (d, J_{C-P} = 10.4 Hz, C_2), 121.2 (d, J_{C-P} = 76.0 Hz, C_1), 116.7 (s, C_7), 114.7 (d, J_{C-P} = 11.7 Hz, C_8), 74.8 (d, J_{C-P} = 97.5 Hz, C_{11}), 33.7 (d, J_{C-P} = 48.2 Hz, C_{Cy}), 27.3 (d, J_{C-P} = 2.5 Hz, C_{Cy}), 27.0 (d, J_{C-P} = 13.8 Hz, C_{Cy}), 26.9 (d, J_{C-P} = 13.1 Hz, C_{Cy}), 26.4 (s, C_{Cy}), 26.4 (s, C_{Cy}), 26.2 (d, J_{C-P} = 1.8 Hz, C_{Cy}). ^{31}P NMR (162 MHz, CD_2Cl_2) δ + 41.6. HRMS (ESI, CH_2Cl_2/CH_3OH: 9/1) $[M+H]^+$ ($C_{30}H_{34}OP$) : m/z Theoretical : 441.2342, m/z Found : 441.2340.

__8__: **General method A** was used with 3,3'-Dibromo-2,2'-bithiophène (3.00 g, 9.27 mmol, 1 eq), *n*-BuLi (4.65 mL, 10.20 mmol, 1.1 eq) and Ph_2PCl (2.24 g, 10.20 mmol, 1 eq) in 150 mL of dry Et_2O. The crude mixture was purified by silica gel chromatography using C7/DCM (8/2) to afford a yellowish oil (1.73 g, η = 44 %). The compound was used without further purification. ^{1}H NMR (300 MHz, CD_2Cl_2) δ 7.44 (d, J = 5.2 Hz, 1H, H_6), 7.39 – 7.31 (m, 11H, $H_{Ph,2}$), 7.06 (d, J = 5.3 Hz, 1H, H_3), 6.79 (d, J = 5.3 Hz, 1H, H_7). ^{13}C NMR (75 MHz, CD_2Cl_2) δ 139.7 (d, J_{C-P} = 37.2 Hz, Cq), 138.5 (d, J_{C-P} = 22.2 Hz, Cq), 138.3 (d, J_{C-P} = 11.1 Hz, Cq), 133.6 (d, J_{C-P} = 19.5 Hz, C_2), 133.5 (d, J_{C-P} = 19.6 Hz, C_{ortho}), 132.7 (d, J_{C-P} = 1.7 Hz, C_7), 130.9 (s, C_3), 128.9 (d, J_{C-P} = 5.1 Hz, C_{meta}), 128.8 (s, C_{para}), 128.0 (d, J_{C-P} = 1.3 Hz, Cq), 127.8 (d, J_{C-P} = 2.2 Hz, C_6), 112.9 (s, Cq). ^{31}P NMR (121 MHz, CD_2Cl_2) δ -25.3.

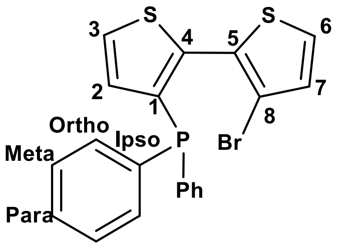

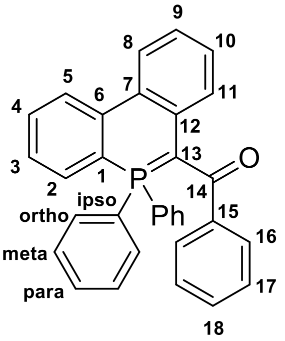

__7a__: ***(General method C)*** **6** (200 mg, 0.51 mmol, 1 eq), 2-bromoacetophenone (111 mg, 1.10 mmol, 1.1 eq) were dissolved in 2 mL of dry DMF and heated at 100°C during 4h. Then the mixture was cooled down to 0°C and ^{t}BuOK (86 mg, 0.56 mmol, 1.5 eq) was added. After stirring for 30 min, the reaction was heated at 175°C during 1 night. After evaporation of the solvent, the crude product was purified through silica gel column chromatography (DCM/Acetone: 95/5). An orange-brown powder was obtained (114 mg, η = 49%). ^{1}H NMR (400 MHz, CD_2Cl_2) δ 8.21 (dd, J = 8.3, 4.9 Hz, 1H, H_2), 7.90 – 7.85 (m, 1H, H_{11}), 7.81 – 7.73 (m, 4H, H_{ortho}), 7.72 – 7.67 (m, 3H, $H_{3,17}$), 7.58 – 7.50 (m, 2H, H_{para}), 7.50 – 7.43 (m, 4H, H_{meta}), 7.39 7.23 (m, 5H, $H_{4,5,16,18}$), 7.04 – 6.99 (m, 1H, H_8), 6.90 – 6.84 (m, 2H, $H_{9,10}$). ^{13}C NMR (101 MHz, CD_2Cl_2) δ 186.9 (d, J_{C-P} = 4.9 Hz, C_{14}), 142.2 (d, J_{C-P} = 10.4 Hz, Cq), 141.2 (d, J_{C-P} = 5.1 Hz, Cq), 136.8 (d, J_{C-P} = 8.1 Hz, Cq), 133.0 (s, C_{18}), 132.9 (d, J_{C-P} = 10.8 Hz, C_{ortho}), 132.1 (d, J_{C-P} = 2.3 Hz, C_3), 131.8 (d, J_{C-P} = 3.1 Hz, C_{para}), 130.1 (d, J_{C-P} = 10.6 Hz, C_8), 129.6 (s, C_{4or5}), 129.3 (s, C_{17}), 128.7 (d, J_{C-P} = 12.8 Hz, C_{meta}), 128.0 (s, C_{16}), 127.6 (s, C_{9or10}), 127.4 (d, J_{C-P} = 91.9 Hz, C_{ipso}), 126.4 (d, J_{C-P} = 11.4 Hz, C_{4or5}), 126.0 (d, J_{C-P} = 2.3

Hz, C_{11}), 125.6 (d, $J_{C\text{-}P}$ = 8.5 Hz, C_2), 125.3 (s, Cq), 120.7 (s, C_{9or10}), 118.3 (d, $J_{C\text{-}P}$ = 87.5 Hz, C_1), 67.5 (d, $J_{C\text{-}P}$ = 106.4 Hz, C_{13}). ^{31}P NMR (121 MHz, CD_2Cl_2) δ +2.2. HRMS (ESI, CH_3OH/CH_2Cl_2 : 9/1) - $[M+Na]^+$ ($C_{32}H_{23}ONaP$) : m/z Theoretical : 477.1379, m/z Found : 477.1374.

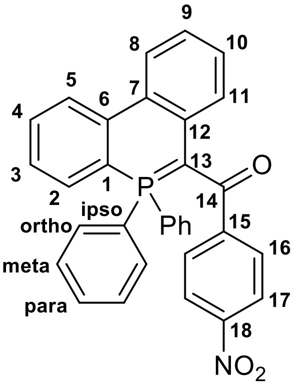

7b: **General method C** was used with **6** (80 mg, 0.20 mmol, 1 eq), 4-Nitrophenacyl bromide (55 mg, 0.22 mmol, 1.1 eq), ^{t}BuOK (34 mg, 0.31 mmol, 1.5 eq) in 1.5 mL of DMF. The product was purified through silica gel column chromatography (DCM/Acetone: 95/5). A yellow powder was obtained. (60 mg, η = 60 %). ^{1}H NMR (300 MHz, CD_2Cl_2) δ 8.21 (dd, J = 8.3, 4.9 Hz, 1H, H_2), 8.15 – 8.05 (m, 2H, H_{17}), 7.92 – 7.80 (m, 3H, $H_{11,16}$), 7.79 – 7.70 (m, 5H, $H_{ortho,\,3}$), 7.60 – 7.51 (m, 2H, H_{para}), 7.51 – 7.41 (m, 4H, H_{meta}), 7.41 – 7.31 (m, 1H, H_4), 7.31 – 7.20 (m, 1H, H_5), 6.98 – 6.79 (m, 3H, $H_{8,9,10}$). ^{13}C NMR (101 MHz, CD_2Cl_2) δ 184.2 (d, $J_{C\text{-}P}$ = 5.5 Hz, C_{14}), 148.8 (d, $J_{C\text{-}P}$ = 10.3 Hz, Cq), 141.6 (d, $J_{C\text{-}P}$ = 5.1 Hz, Cq), 136.2 (d, $J_{C\text{-}P}$ = 7.6 Hz, Cq), 133.5 (d, $J_{C\text{-}P}$ = 7.5 Hz, C_5), 133.4 (d, $J_{C\text{-}P}$ = 10.7 Hz, C_{ortho}), 132.9 (d, $J_{C\text{-}P}$ = 2.1 Hz, C_3), 132.6 (d, $J_{C\text{-}P}$ = 3.1 Hz, C_{para}), 130.9 (d, $J_{C\text{-}P}$ = 10.3 Hz, C_8), 130.8 (s, C_{16}), 129.3 (d, $J_{C\text{-}P}$ = 12.9 Hz, C_{meta}), 128.4 (s, C_{10}), 127.5 (s, Cq), 127.08 (d, $J_{C\text{-}P}$ = 11.5 Hz, Cq), 127.04 (d, $J_{C\text{-}P}$ = 92.0 Hz, C_{ipso}), 126.6 (d, $J_{C\text{-}P}$ = 2.3 Hz, C_{11}), 126.5 (d, $J_{C\text{-}P}$ = 8.6 Hz, Cq), 126.2 (d, $J_{C\text{-}P}$ = 8.8 Hz, C_2), 123.7 (s, C_{17}), 122.1 (s, C_9), 118.6 (d, $J_{C\text{-}P}$ = 88.3 Hz, C_1), 69.5 (d, $J_{C\text{-}P}$ = 106.0 Hz, C_{13}). ^{31}P NMR (121 MHz, CD_2Cl_2) δ +2.6. HRMS (ESI, CH_2Cl_2/CH_3OH: 9/1) $[M+H]^+$ ($C_{32}H_{23}NO_3P$) : m/z Theoretical : 500.1410, m/z Found : 500.1405.

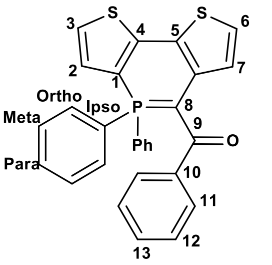

9: General **method C** was used with **8** (1.73 g, 4.05 mmol, 1 eq), 2-bromoacetophenone (0.89 g, 4.45 mmol, 1.1 eq) and ^{t}BuOK (0.60 g, 5.38 mmol, 1.5 eq) in 17 mL of dry DMF. After evaporation of the solvent, the crude product was purified through silica gel column chromatography (DCM/Acetone: 95/5). An orange-brown powder was obtained (1.44 g, η = 76 %). ^{1}H NMR (400 MHz, CD_2Cl_2) δ 7.84 (ddd, J = 13.8, 6.7, 1.6 Hz, 4H, H_{Ortho}), 7.60 – 7.46 (m, 8H, $H_{11,meta,para}$), 7.46 – 7.32 (m, 3H, $H_{12,13}$), 7.13 (dd, J = 5.3, 2.7 Hz, 1H, H_2), 6.93 – 6.88 (m, 2H, $H_{3,6}$), 6.36 (dd, J = 5.4, 1.1 Hz, 1H, H_7). ^{13}C NMR (101 MHz, CD_2Cl_2) δ 188.9 (d, $J_{C\text{-}P}$ = 4.3 Hz, C_9), 147.6 (d, $J_{C\text{-}P}$ = 8.8 Hz, Cq), 142.8 (d, $J_{C\text{-}P}$ = 10.4 Hz, Cq), 138.1 (d, $J_{C\text{-}P}$ = 10.1 Hz, Cq), 133.2 (d, $J_{C\text{-}P}$ = 11.5 Hz, C_{ortho}), 131.9 (d, $J_{C\text{-}P}$ = 3.2 Hz, C_{para}), 129.9 (s, C_{13}), 129.1 (d, $J_{C\text{-}P}$ = 10.5 Hz, C_3), 128.9 (s, C_{12}), 128.8 (s, C_{11}), 128.6 (d, $J_{C\text{-}P}$ = 6.9 Hz, C_{meta}), 128.4 (d, $J_{C\text{-}P}$ = 97.7 Hz, C_{ipso}), 127.8 (d, $J_{C\text{-}P}$ = 12.4 Hz, C_7), 123.76 (s, C_6), 122.38 (d, $J_{C\text{-}P}$ = 15.1 Hz, C_2), 115.0 (d, $J_{C\text{-}P}$ = 7.3 Hz, Cq), 110.5 (d, $J_{C\text{-}P}$ = 89.8 Hz, C_1), 72.0 (d, $J_{C\text{-}P}$ = 110.5 Hz, C_8). ^{31}P NMR (162 MHz, CD_2Cl_2) δ +1.6. HRMS (ESI, CH_3OH/CH_2Cl_2 : 90/10) M^+ ($C_{28}H_{19}O_2PS_2$) : m/z Calculated : 466.0610, m/z Found : 466.0615.

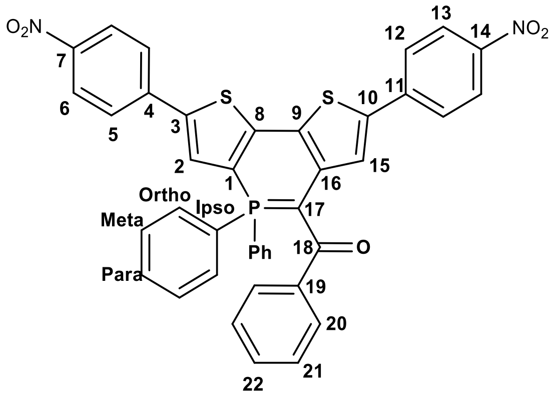

10: (***General method D***) **9** (200 mg, 0.43 mmol, 1 eq), K_2CO_3 (236 mg, 1.74 mmol, 4 eq), $Pd(OAc)_2$ (10 mg, 0.04 mmol, 0.1 eq), 4-iodonitrobenzene (500 mg, 1.74 mmol, 4 eq), PCy_3HBF_4 (32 mg, 0.09 mmol, 0.2 eq), PivOH (14 mg, 0.13 mmol, 0.3 eq) were dissolved in 2 mL of DMAc under argon. The mixture was heated at 125°C for 20h. DMAc was then evaporated and the product was purified through silica gel column (CH_2Cl_2/AcOEt : 95/5) to afford a dark purple solid (m = 158 mg, η = 52 %). ^{1}H NMR (400 MHz, CD_2Cl_2) δ 8.24 – 8.18 (m, 2H, H_6), 8.17 – 8.09 (m, 2H, H_{13}), 7.90 (ddd, J = 14.0, 6.8, 1.6 Hz, 4H, H_{ortho}), 7.69 – 7.64 (m, 2H, H_5), 7.64 – 7.50 (m, 9H, $H_{meta,para,,20,22}$), 7.46 (dd, J = 8.1, 6.7 Hz, 2H, H_{21}), 7.40 – 7.33 (m, 2H, H_{12}), 7.29 (d, J = 3.5 Hz, 1H, H_2), 6.69 (d, J = 1.1 Hz, 1H, H_{15}). ^{13}C NMR (126 MHz, CD_2Cl_2) δ 189.6 (d, J_{C-P} = 88.5 Hz, C_{18}), 147.3 (d, J_{C-P} = 7.6 Hz, C_8), 147.2 (s, C_7), 147.2 (s, C_{14}), 142.4 (d, J_{C-P} = 10.2 Hz, C_{19}), 140.7 (d, J_{C-P} = 10.4 Hz, C_9), 140.2 (s, C_{11}), 139.8 (s, C_{10}), 139.4 (s, C_4), 138.7 (d, J_{C-P} = 14.7 Hz, C_3), 133.3 (d, J_{C-P} = 11.6 Hz, C_{ortho}), 132.4 (d, J = 3.3 Hz, C_{para}), 130.4 (s, C_{22}), 129.1 (d, J_{C-P} = 13.4 Hz, C_{meta}), 128.9 (s, C_{21}), 128.43 (s, C_{20}), 127.7 (d, J_{C-P} = 97.9 Hz, C_{ipso}), 127.6 (d, J_{C-P} = 10.3 Hz, C_2), 126.3 (s, C_5), 126.3 (d, J_{C-P} = 12.5 Hz, C_{15}), 126.2 (s, C_{12}), 124.8 (s, C_6), 124.7 (s, C_{13}), 116.3 (s, C_{16}), 113.2 (d, J_{C-P} = 88.5 Hz, C_1), 72.5 (d, J_{C-P} = 111.0 Hz, C_{17}). ^{31}P NMR (162 MHz, CD_2Cl_2) δ +0.9. HRMS (ESI, CH_3OH/CH_2Cl_2 : 90/10) $[M+H]^+$ ($C_{40}H_{26}N_2O_5PS_2$) : m/z Calculated : 709.1015, m/z Found : 709.1018.

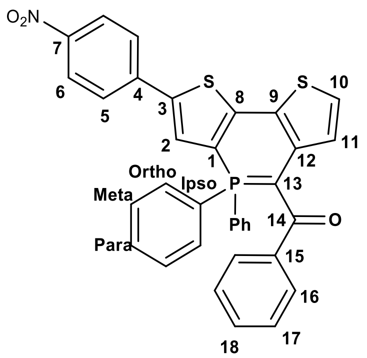

10': During the synthesis of **10**, a second product was purified through silica gel column (CH_2Cl_2/AcOEt : 95/5) to afford a dark purple solid (m = 57 mg, η = 23 %). ^{1}H NMR (400 MHz, CD_2Cl_2) δ 8.22 – 8.15 (m, 2H, H_6), 7.86 (ddd, J = 14.0, 6.8, 1.6 Hz, 4H, H_{ortho}), 7.67 – 7.61 (m, 2H, H_5), 7.58 – 7.48 (m, 8H, $H_{meta,para,16}$), 7.43 – 7.35 (m, 3H, $H_{17,18}$), 7.25 (d, J = 3.5 Hz, 1H, H_2), 6.96 (dd, J = 5.4, 2.4 Hz, 1H, H_{11}), 6.35 (dd, J = 5.4, 1.1 Hz, 1H, H_{10}). ^{13}C NMR (126 MHz, CD_2Cl_2) δ 189.4 (d, J_{C-P} = 4.3 Hz, C_{14}), 148.4 (d, J_{C-P} = 8.0 Hz, C_8), 147.1 (s, C_7), 142.5 (d, J_{C-P} = 10.3 Hz, C_{15}), 139.7 (d, J_{C-P} = 9.9 Hz, C_9), 139.6 (s, C_4), 137.7 (d, J_{C-P} = 14.7 Hz, C_3), 133.2 (d, J_{C-P} = 11.4 Hz, C_{ortho}), 132.2 (d, J_{C-P} = 3.0 Hz, C_{para}), 130.1 (s, C_{18}), 129.0 (d, J_{C-P} = 13.3 Hz, C_{meta}), 128.7 (s, C_{17}), 128.50 (s, C_{16}), 128.0 (d, J_{C-P} = 97.8 Hz, C_{ipso}), 127.8 (d, J_{C-P} = 12.5 Hz, C_{10}), 127.5 (d, J_{C-P} = 10.4 Hz, C_2), 126.1 (s, C_5), 125.2 (s, C_{11}), 124.8 (s, C_6), 114.5 (d, J_{C-P} = 7.5 Hz, C_{12}), 112.1 (d, J_{C-P} = 89.0 Hz, C_1), 72.8 (d, J_{C-P} = 109.6 Hz, C_{13}).^{31}P NMR (162 MHz, CD_2Cl_2) δ +1.1. HRMS (ESI, CH_3OH/CH_2Cl_2 : 90/10) $[M+H]^+$ ($C_{34}H_{23}NO_3PS_2$) : m/z Calculated : 588.0852, m/z Found : 588.0851.

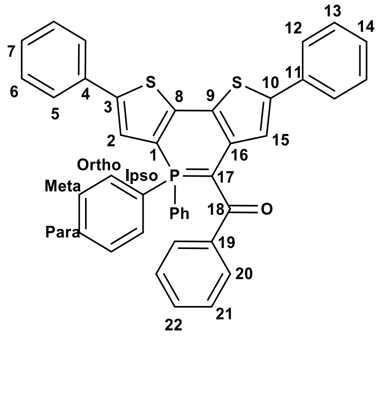

11 : General method D was used with **9** (200 mg, 0.43 mmol, 1 eq), K_2CO_3 (236 mg, 1.74 mmol, 4 eq), $Pd(OAc)_2$ (10 mg, 0.04 mmol, 0.1 eq), 4-bromobenzene (400 mg, 2.54 mmol, 4 eq), PCy_3HBF_4 (32 mg, 0.09 mmol, 0.2 eq), PivOH (14 mg, 0.13 mmol, 0.3 eq) as starting material to afford an orange solid (m = 225 mg, η = 85 %) after purification on silica gel column (CH_2Cl_2/AcOEt : 99/1). ^{1}H NMR (400 MHz, CD_2Cl_2) δ 7.93 (dd, J = 13.9 Hz, J = 6.5 Hz, 4H, H_{ortho}), 7.67 – 7.60 (m, 2H, H_{20}), 7.58 – 7.50 (m, 8H, $H_{12,13,meta}$), 7.50 – 7.43 (m, 3H, $H_{21,22}$), 7.38 (t, J = 8.3 Hz, 2H, H_{para}), 7.33 – 7.21 (m, 6H, $H_{5,6,7,14}$), 7.14 (d, J = 3.4 Hz, 1H, H_2),

6.57 (d, J = 1.1 Hz, 1H, H_{15}). ^{13}C NMR (101 MHz, CD_2Cl_2) δ 189.3 (d, J_{C-P} = 4.5 Hz, C_{18}), 146.3 (d, J_{C-P} = 7.8 Hz, Cq), 142.8 (d, J_{C-P} = 10.3 Hz, Cq), 141.9 (s, Cq), 141.0 (d, J_{C-P} = 14.7 Hz, Cq), 139.3 (d, J_{C-P} = 10.1 Hz, Cq), 133.8 (d, J_{C-P} = 83.0 Hz, C_{ipso}), 133.3 (d, J_{C-P} = 11.4 Hz, C_{ortho}), 132.0 (d, J_{C-P} = 3.1 Hz, C_{12}), 130.0 (s, C_{22}), 129.4 (d, J_{C-P} = 2.8 Hz, C_{para}), 129.2 (s, C_5), 128.9 (d, J_{C-P} = 13.2 Hz, C_{meta}), 128.7 (s, C_{21}), 128.6 (s, Cq), 128.5 (s, C_{20}), 128,4 (s, C_{14}), 128.2 (s, C_7), 126.7 (s, Cq), 126.0 (s, C_{13}), 125.8 (s, C_6), 124.5 (d, J_{C-P} = 10.5 Hz, C_2), 123.8 (d, J_{C-P} = 12.7 Hz, C_{15}), 114.6 (d, J_{C-P} = 7.9 Hz, Cq), 111.3 (d, J_{C-P} = 89.6 Hz, C_1), 72.4 (d, J_{C-P} = 110.6 Hz, C_{17}). ^{31}P NMR (162 MHz, CD_2Cl_2) δ +0.9. HRMS (ESI, CH_3OH/CH_2Cl_2: 90/10) $[M+H]^+$ ($C_{40}H_{28}OPS_2$) : m/z Calculated : 619.1314, m/z Found : 619.1315.

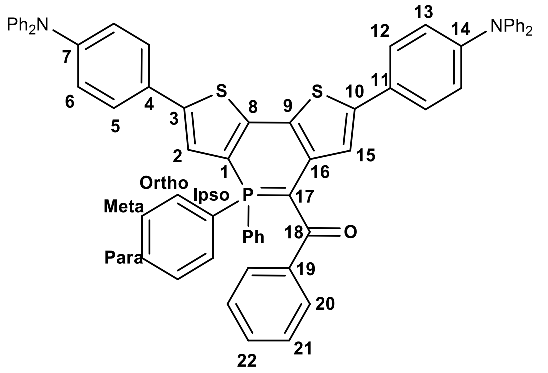

<u>12 :</u> General method D was used with **9** (200 mg, 0.43 mmol, 1 eq), K_2CO_3 (236 mg, 1.74 mmol, 4 eq), $Pd(OAc)_2$ (10 mg, 0.04 mmol, 0.1 eq), 4-bromotriphenylamine (565 mg, 1.74 mmol, 4 eq), PCy_3HBF_4 (32 mg, 0.09 mmol, 0.2 eq), PivOH (14 mg, 0.13 mmol, 0.3 eq) as starting material to afford an orange solid (m = 405 mg, η = 99 %) after purification on silica gel column (CH_2Cl_2/Acetone: 95/5). ^1H NMR (400 MHz, CD_2Cl_2) δ 7.92 (ddd, J = 13.7 Hz, J = 7.6 Hz, J = 1.6 Hz, 4H, H_{Ortho}), 7.65 – 7.58 (m, 2H, H_{20}), 7.58 – 7.47 (m, 6H, $H_{meta, para}$), 7.45 – 7.39 (m, 3H, $H_{21,22}$), 7.38 – 7.34 (m, 2H, H_6), 7.27 (t, J = 7,6 Hz, 8H, H_{NPh2}), 7.12 – 7.04 (m, 15H, $H_{2,13,NPh2}$), 7.00 (dd, J = 6.3 Hz, J = 2.7 Hz, 2H, H_5), 6.95 – 6.90 (m, 2H, H_{12}), 6.46 (d, J = 1.0 Hz, 1H, H_{15}). ^{13}C NMR (101 MHz, CD_2Cl_2) δ 189.2 (d, J_{C-P} = 4.4 Hz, C_{18}), 148.0 (d, J_{C-P} = 12.7 Hz, Cq), 147.7 (d, J_{C-P} = 6.0 Hz, Cq), 145.6 (d, J_{C-P} = 7.9 Hz, Cq), 142.9 (d, J_{C-P} = 10.2 Hz, Cq), 141.7 (s, Cq), 140.8 (d, J_{C-P} = 15.0 Hz, Cq), 139.1 (d, J_{C-P} = 10.2 Hz, Cq), 133.3 (d, J_{C-P} = 11.4 Hz, C_{ortho}), 132.0 (d, J_{C-P} = 3.1 Hz, C_{para}), 130.0 (s, C_{22}), 129.7 (d, J_{C-P} = 2.3 Hz, C_{NPh2}), 128.9 (d, J_{C-P} = 13.2 Hz, C_{meta}), 128.7 (s, C_{21}), 128.5 (s, C_{20}), 128.2 (d, J_{C-P} = 97.2 Hz, C_{ipso}), 128.0 (s, Cq), 127.13 (s, Cq), 126.73 (s, C_6), 126.6 (s, C_{13}), 125.1 (d, J_{C-P} = 3.8 Hz, C_{NPh2}), 123.8 (d, J_{C-P} = 7.2 Hz, C_{12}), 123.5 (d, J_{C-P} = 12.8 Hz, C_5), 123.3 (d, J_{C-P} = 10.5 Hz, C_{15}), 122.7 (d, J_{C-P} = 12.6 Hz, C_2), 114.1 (d, J_{C-P} = 7.9 Hz, Cq), 110.9 (d, J_{C-P} = 89.5 Hz, C_{ipso}), 72.4 (d, J_{C-P} = 110.2 Hz, C_{17}). ^{31}P NMR (162 MHz, CD_2Cl_2) δ + 0.8. HRMS (ESI, CH_3OH/CH_2Cl_2: 90/10) $[M+H]^+$ ($C_{64}H_{46}OPS_2$) : m/z Calculated : 953.2784, m/z Found : 953.2783.

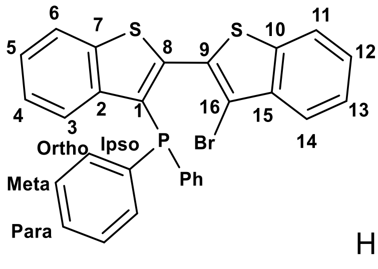

H

<u>13</u>: **General method A** was used with 3,3'-dibromo-2,2'-bibenzo[*b*]thiophene (600 mg, 1.41 mmol, 1 eq), 30 mL of dry Et_2O, BuLi (0.65 mL, 1.56 mmol, 1.1 eq) and Ph_2PCl (326 g, 1.56 mmol, 1.1 eq). The crude mixture was purified by silica gel chromatography (DCM/AcOEt: 1/1) to afford a white powder (320 mg, η = 43 %). ^1H NMR (400 MHz, CD_2Cl_2) δ 7.86 (d, J = 8.1 Hz, 1H, H_3), 7.48 – 7.13 (m, 16H, $H_{5,6,11,12,13,14, ortho, meta, para}$), 7.06 (t, J = 7.7 Hz, 1H, H_4). ^{13}C NMR (101 MHz, CD_2Cl_2) δ 147.2 (d, J = 54.5 Hz, Cq), 141.9 (s, Cq), 140.7 – 140.6 (m, Cq), 136.2 – 135.5 (m, Cq), 133.1 – 132.2 (m, CH), 128.8 - 128.6 (m, CH), 128.4 (s, CH), 126.1 (s, CH), 125.4 (s, CH), 124.7 (s, CH), 122.7 (s, CH). **^{31}P NMR** (162 MHz, CD_2Cl_2) δ - 26.9. HRMS: MALDI TOF: $[M+H]^+$ ($C_{28}H_{19}BrPS_2$) : m/z Calculated : 528.9849, m/z Found : 528.854.

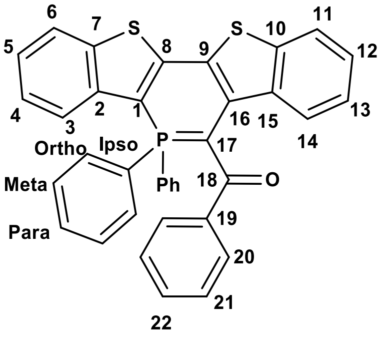

14: **General method C** was used with **13** (320 mg, 0.61 mmol, 1 eq), 2-bromoacetophenone (130 mg, 0.67 mmol, 1.1 eq), ^tBuOK (102 mg, 1.50 mmol, 1.5 eq) in 4 mL of DMF. After evaporation of the solvent, the crude product was purified through silica gel column chromatography (DCM/Acetone: 99/1). A red powder was obtained. (285 mg, η = 83 %). ^1H NMR (300 MHz, CD_2Cl_2) δ 7.97 (ddd, J = 13.8, 6.9, 1.5 Hz, 4H, H_{ortho}), 7.88 (d, J = 8.0 Hz, 1H, H_3), 7.69 – 7.60 (m, 3H, $H_{14,20}$), 7.57 – 7.49 (m, 2H, H_{para}), 7.49 – 7.40 (m, 4H, H_{meta}), 7.31 – 7.21 (m, 3H, $H_{4,13,22}$), 7.19 – 7.07 (m, 4H, $H_{5,11,21}$), 6.98 (d, J = 8.3 Hz, 1H, H_6), 6.81 (t, J = 8.3 Hz, 1H, H_{12}). ^{13}C NMR (101 MHz, CD_2Cl_2) δ 187.0 (d, J_{C-P} = 5.4 Hz, C_{18}), 148.5 (d, J_{C-P} = 8.1 Hz, Cq), 142.8 (d, J_{C-P} = 10.0 Hz, Cq), 140.3 (d, J_{C-P} = 9.6 Hz, Cq), 139.9 (s, Cq), 138.6 (d, J_{C-P} = 13.4 Hz, Cq), 138.3 (d, J_{C-P} = 11.6 Hz, Cq), 136.0 (d, J_{C-P} = 8.4 Hz, Cq), 133.2 (d, J_{C-P} = 11.4 Hz, C_{ortho}), 132.3 (d, J_{C-P} = 3.2 Hz, C_{para}), 130.5 (s, C_{22}), 129.5 (s, C_{20}), 129.1 (d, J_{C-P} = 13.1 Hz, C_{meta}), 128.3 (s, C_{21}), 127.9 (d, J_{C-P} = 93.5 Hz, C_{ipso}),127.6 (s, C_{13}), 125.7 (d, J_{C-P} = 4.2 Hz, C_5), 124.8 (s, C_4), 123.5 (s, C_{12}), 122.9 (s, C_3), 122.8 (s, C_6), 122.7 (s, C_{14}), 115.0 (d, J_{C-P} = 8.5 Hz, Cq), 103.7 (d, J_{C-P} = 94.3 Hz, C_1), 70.9 (d, J_{C-P} = 110.2 Hz, C_{17}).^{31}P NMR (162 MHz, CD_2Cl_2) δ +6.9. HRMS (ESI, CH_3OH/CH_2Cl_2 : 90/10) $[M+H]^+$ ($C_{36}H_{24}OPS_2$) : m/z Calculated : 567.1001, m/z Found : 567.0997.

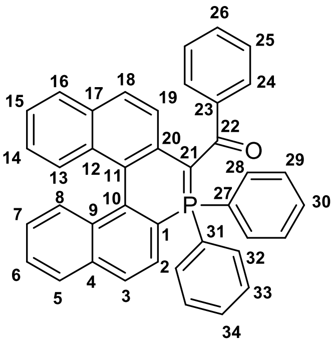

16a: **General method C** was used with **15** (200 mg, 0.39 mmol, 1 eq) and 2-Bromoacetophenone (85 mg, 0.43 mmol, 1.1 eq), ^tBuOK (65 mg, 0.58 mmol, 1.5 eq) in 2 mL of DMF. The product was purified through silica gel column chromatography (DCM/Acetone: 95/5). An orange powder was obtained (133 mg, η = 62 %). ^1H NMR (500 MHz, CD_2Cl_2) δ 7.96 – 7.90 (m, 3H, $H_{5,28}$), 7.87 – 7.81 (m, 3H, $H_{3,24}$), 7.74 (d, J = 8.7 Hz, 1H, H_8), 7.61 – 7.58 (m, 2H, $H_{6,16}$), 7.58 – 7.48 (m, 4H, $H_{30,32,34}$), 7.47 – 7.38 (m, 5H, $H_{26,29,33}$), 7.38 – 7.28 (m, 5H, $H_{7,18,19,25}$), 7.20 – 7.14 (m, 3H, $H_{2,13,15}$), 7.02 – 6.97 (m, 1H, H_{14}). ^{13}C NMR (126 MHz, CD_2Cl_2) δ 186.0 (d, J_{C-P} = 6.3 Hz, C_{22}), 142.3 (d, J_{C-P} = 10.9 Hz, C_{23}), 141.0 (d, J_{C-P} = 4.6 Hz, C_{10}), 138.1 (d, J_{C-P} = 7.0 Hz, C_{20}), 135.2 (d, J_{C-P} = 2.1 Hz, C_4), 133.6 (d, J_{C-P} = 11.3 Hz, C_{28}), 133.6-133.3 (m, $C_{12,32}$), 132.9 (d, J_{C-P} = 3.0 Hz, C_{30}), 132.2 (d, J_{C-P} = 3.1 Hz, C_{34}), 131.3 (d, J_{C-P} = 9.3 Hz, C_9), 130.5 (s, C_{26}), 130.2 (s, C_{24}), 130.1 (s, C_8), 130.1 (d, J_{C-P} = 8.3 Hz, C_{19}), 129.7 (s, C_{17}), 129.5 (d, J_{C-P} = 12.4 Hz, C_{29}), 129.1 (d, J_{C-P} = 13.1 Hz, C_{33}), 128.5 (s, $C_{5,25}$), 128.2 (s, C_6), 128.1 (s, C_{16}), 128.1 (s, C_{18}), 127.3 (s, C_{13}), 127.2 (d, J_{C-P} = 12.2 Hz, C_3), 127.0 (d, J_{C-P} = 8.7 Hz, C_2), 126.5 (d, J_{C-P} = 84.2 Hz, C_{31}), 126.5 (d, J_{C-P} = 94.0 Hz, C_{27}), 126.2 (s, C_7), 125.4 (s, C_{14}), 123.5 (s, C_{15}), 120.8 (s, C_{11}), 120.1 (d, J_{C-P} = 95.9 Hz, C_1), 71.3 (d, J_{C-P} = 108.6 Hz, C_{21}). ^{31}P NMR (162 MHz, CD_2Cl_2) δ +3.8. HRMS (ESI, CH_3OH /CH_2Cl_2 : 95/5) $[M+H]^+$ ($C_{40}H_{28}OP$) : m/z Theoretical : 555.1872, m/z Found : 555.1870.

P-(+)-16a: $[\alpha]_D^{23}$ = +346 (± 5%) (DCM, 8.2 10^{-3} mol·L^{-1}).

M-(-)-16a:$[\alpha]_D^{23}$=-350 (± 5%) (DCM, 1.2 10^{-3} mol·L−1).

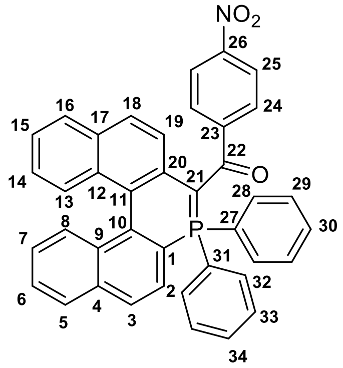

<u>16b</u>: **General method C** was used with **15** (190 mg, 0.37 mmol, 1 eq) and 4-Nitrophenacyl bromide (99 mg, 0.40 mmol, 1.1 eq), ^{t}BuOK (62 mg, 0.55 mmol, 1.5 eq.) in 2 mL of DMF. The product was purified through silica gel column chromatography (DCM/Acetone: 95/5). A dark red powder was obtained (155 mg, η = 70 %).

^{1}H NMR (400 MHz, CD_2Cl_2) δ 8.14 (d, J = 8.2 Hz, 2H, H_{25}), 8.04 – 7.83 (m, 6H, $H_{3,5,24,28}$), 7.71 (d, J = 8.7 Hz, 1H, H_8), 7.64 – 7.37 (m, 11H, $H_{6,16,18,29,30,32,33,34}$), 7.36 – 7.29 (m, 1H, H_7), 7.23 – 7.10 (m, 4H, $H_{2,13,15,19}$), 7.04 – 6.97 (m, 1H, H_{14}). ^{13}C NMR (101 MHz, CD_2Cl_2) δ 182.8 (d, J_{C-P} = 7.5 Hz, C_{22}), 141.2 (d, J_{C-P} = 5.2 Hz, C_{10}), 136.9 (d, J_{C-P} = 5,1 Hz, C_{23}), 135.3 (d, J_{C-P} = 2.0 Hz, C_4), 133.8-133.4 (m, $C_{28,\,32}$), 133.2 (d, J_{C-P} = 2.9 Hz, C_{30}), 132.5 (d, J_{C-P} = 2.0 Hz, C_{18}), 131.4 (d, J_{C-P} = 9.6 Hz, C_9), 131.1 (s, C_{24}), 130.2 (s, C_{17}), 130.1 (d, J_{C-P} = 7.9 Hz, C_{19}), 130.0 (s, C_8), 129.6 (d, J_{C-P} = 12.4 Hz, C_{29}), 129.2 (d, J_{C-P} = 12.2 Hz, C_{33}), 128.6 (s, C_{18}), 128.5 (s, C_5), 128.5 (s, C_6), 128.3 (s, C_{16}), 127.6 (d, J_{C-P} = 12.2 Hz, C_3), 127.5 (s, C_{13}), 127.0 (d, J_{C-P} = 7.2 Hz, C_2), 126.5 (s, C_7), 125.7 (s, C_{14}), 124.0 (s, C_{15}), 123.7 (s, C_{25}), 122.0 (s, C_{11}), 120.3 (d, J_{C-P} = 98.0 Hz, C_1), 72.6 (d, J_{C-P} = 107.1 Hz, C_{21}). ^{31}P NMR (162 MHz, CD_2Cl_2) δ +4.06. HRMS (ESI, CH_2Cl_2/CH_3OH : 95/5) - $[M+Na]^+$ ($C_{40}H_{26}NO_3NaP$) : m/z Theoretical : 622.1543, m/z Found : 622.1544.

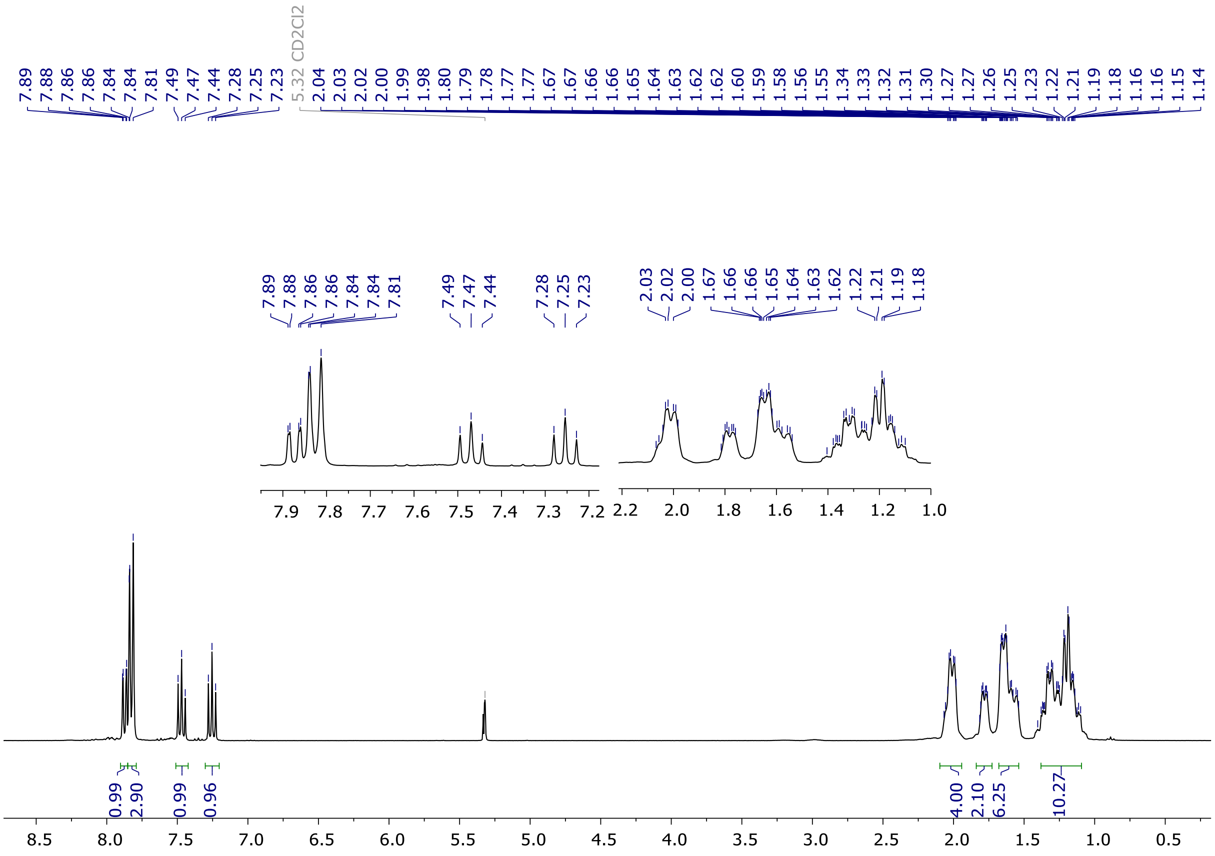

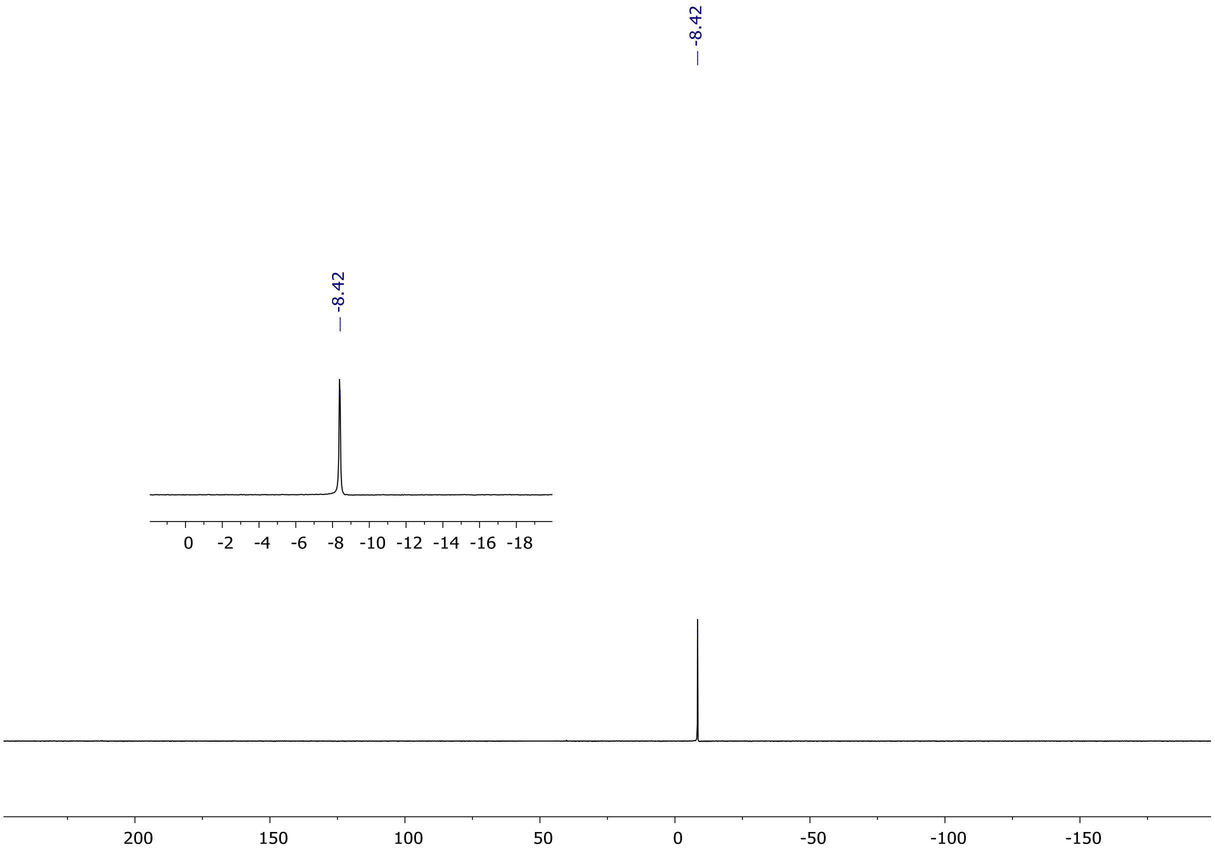

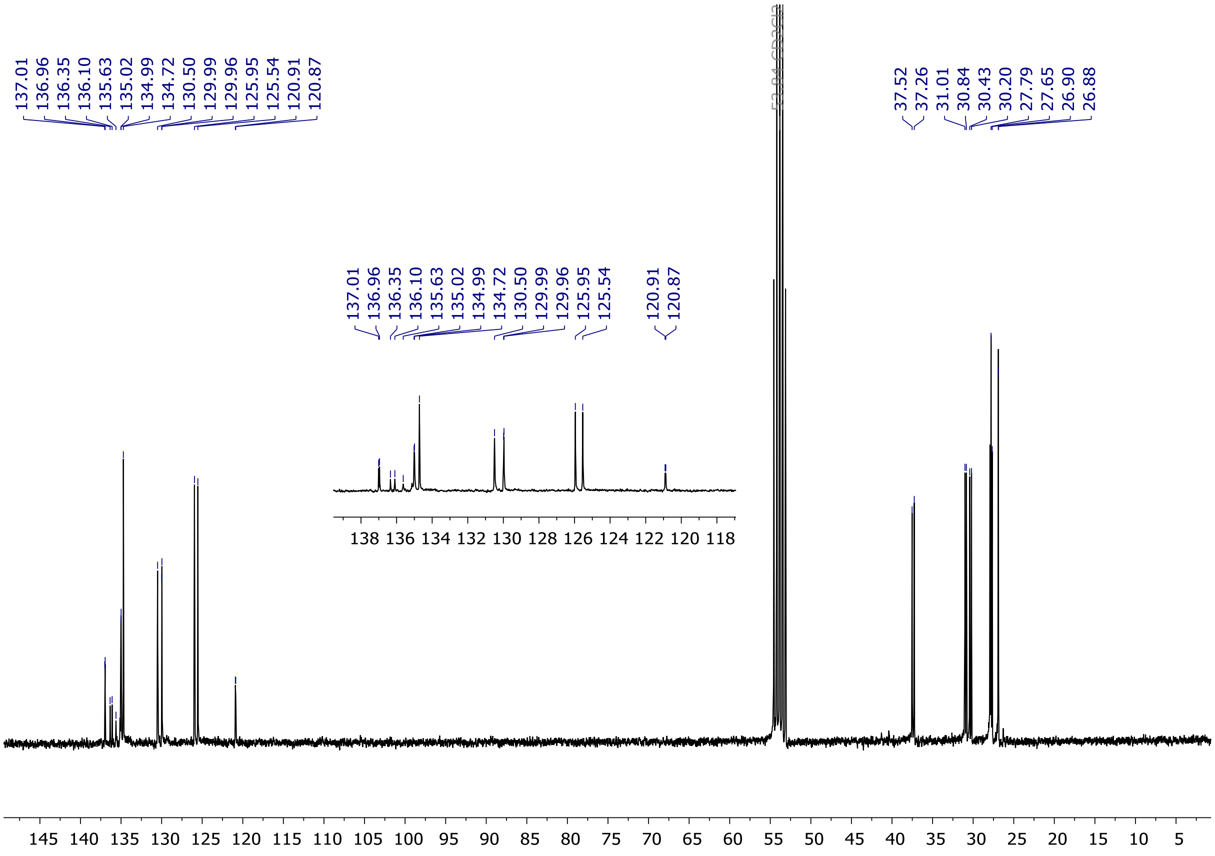

Figure S1: ^{1}H NMR (300 MHz, CD_2Cl_2, 293K), ^{13}C NMR (75 MHz, CD_2Cl_2, 293K) and $^{31}P\{^{1}H\}$ NMR (121 MHz, CD_2Cl_2, 293K) spectra of compound **1b.**

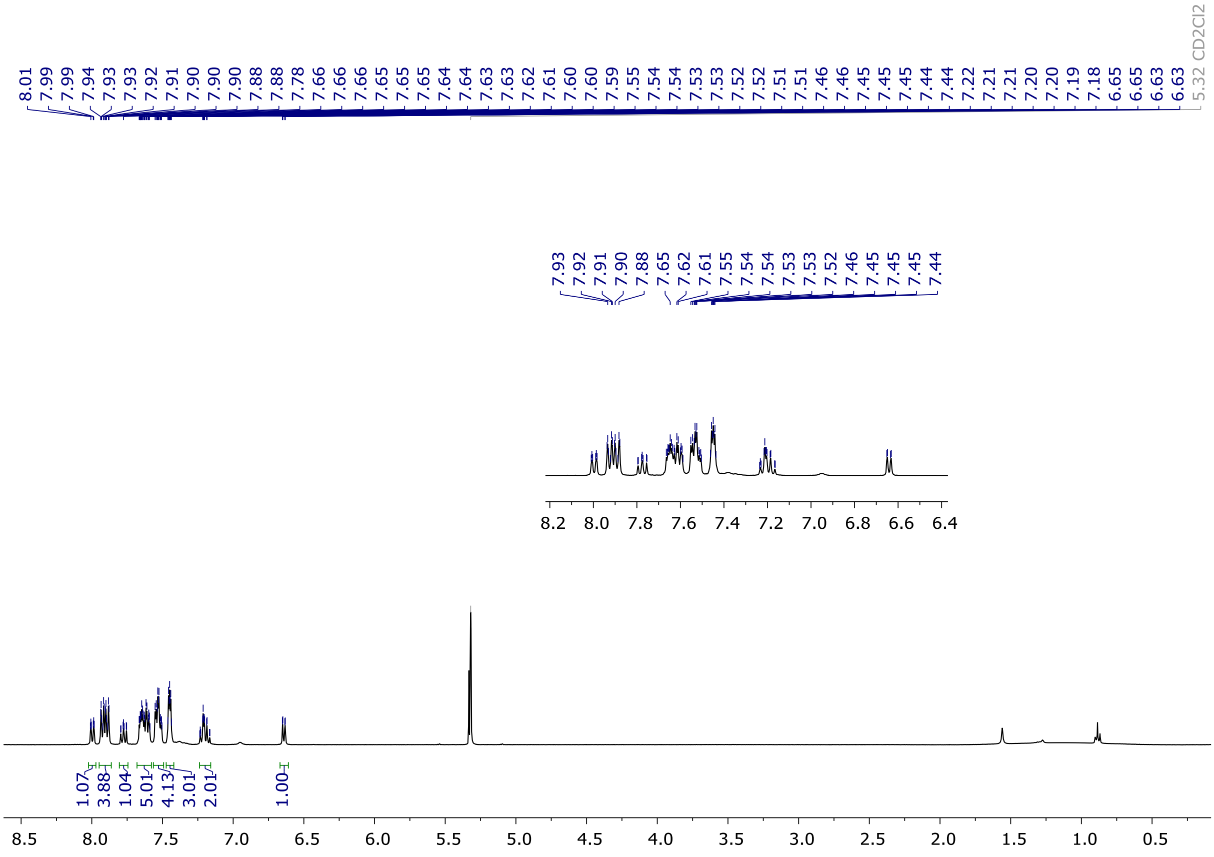
8.01 7.99 7.99 7.94 7.93 7.93 7.92 7.91 7.90 7.90 7.88 7.88 7.78 7.66 7.66 7.65 7.65 7.65 7.64 7.63 7.63 7.62 7.61 7.60 7.60 7.59 7.55 7.54 7.54 7.53 7.53 7.52 7.52 7.51 7.51 7.46 7.46 7.45 7.45 7.44 7.44 7.22 7.21 7.21 7.20 7.20 7.19 7.18 6.65 6.65 6.63 6.63
5.32 CD2Cl2
7.93 7.92 7.91 7.90 7.88 7.65 7.62 7.61 7.55 7.54 7.54 7.53 7.53 7.52 7.46 7.45 7.45 7.45 7.44
8.2 8.0 7.8 7.6 7.4 7.2 7.0 6.8 6.6 6.4
1.07 3.88 1.04 5.01 4.13 3.01 2.01 1.00
8.5 8.0 7.5 7.0 6.5 6.0 5.5 5.0 4.5 4.0 3.5 3.0 2.5 2.0 1.5 1.0 0.5

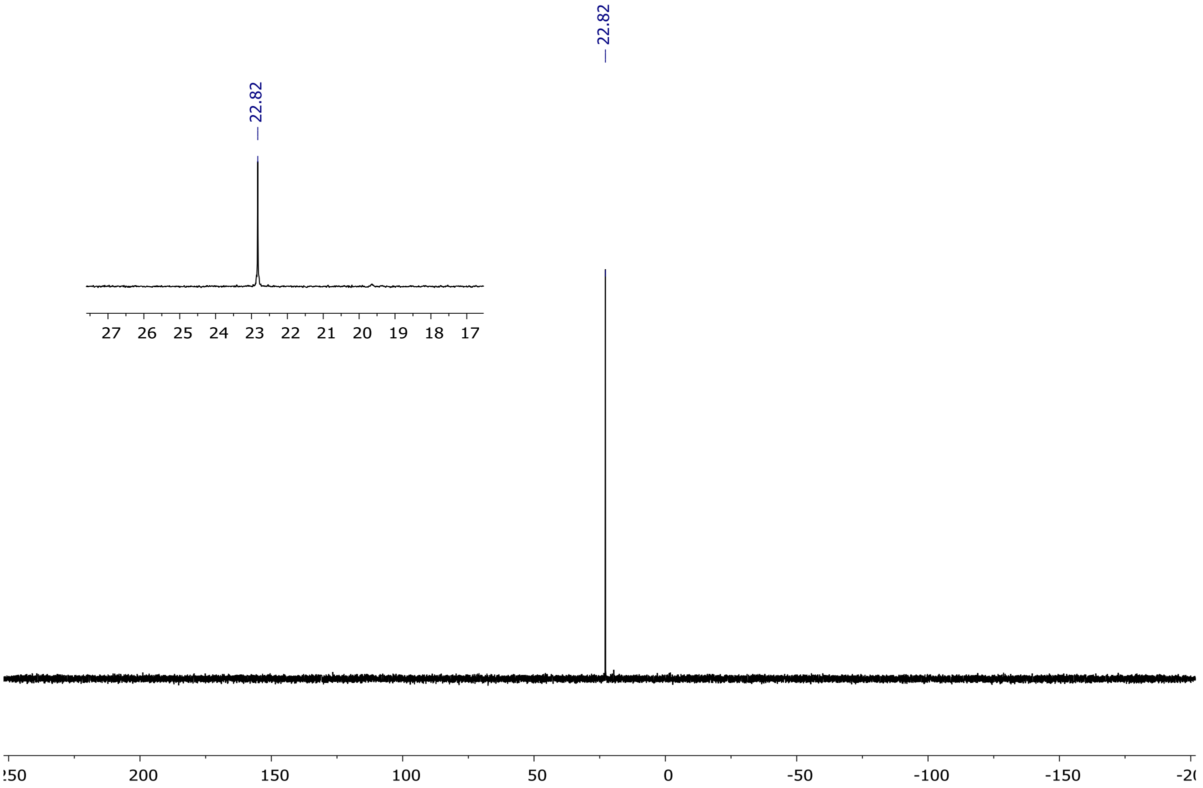
22.82
22.82
27 26 25 24 23 22 21 20 19 18 17
250 200 150 100 50 0 -50 -100 -150 -200

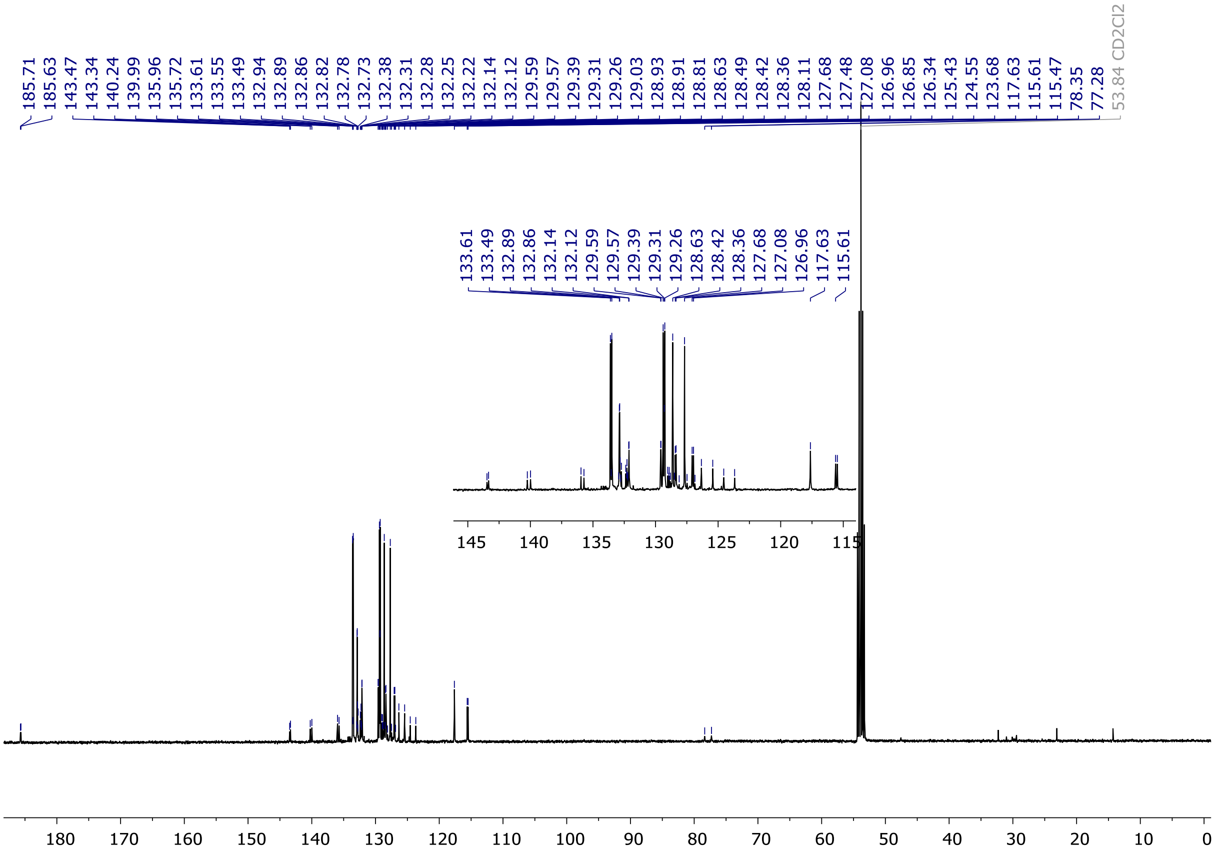

Figure S2: ^{1}H NMR (400 MHz, CD_2Cl_2, 293K), ^{13}C NMR (101 MHz, CD_2Cl_2, 293K) and $^{31}P\{^{1}H\}$ NMR (162 MHz, CD_2Cl_2, 293K) spectra of compound **5a.**

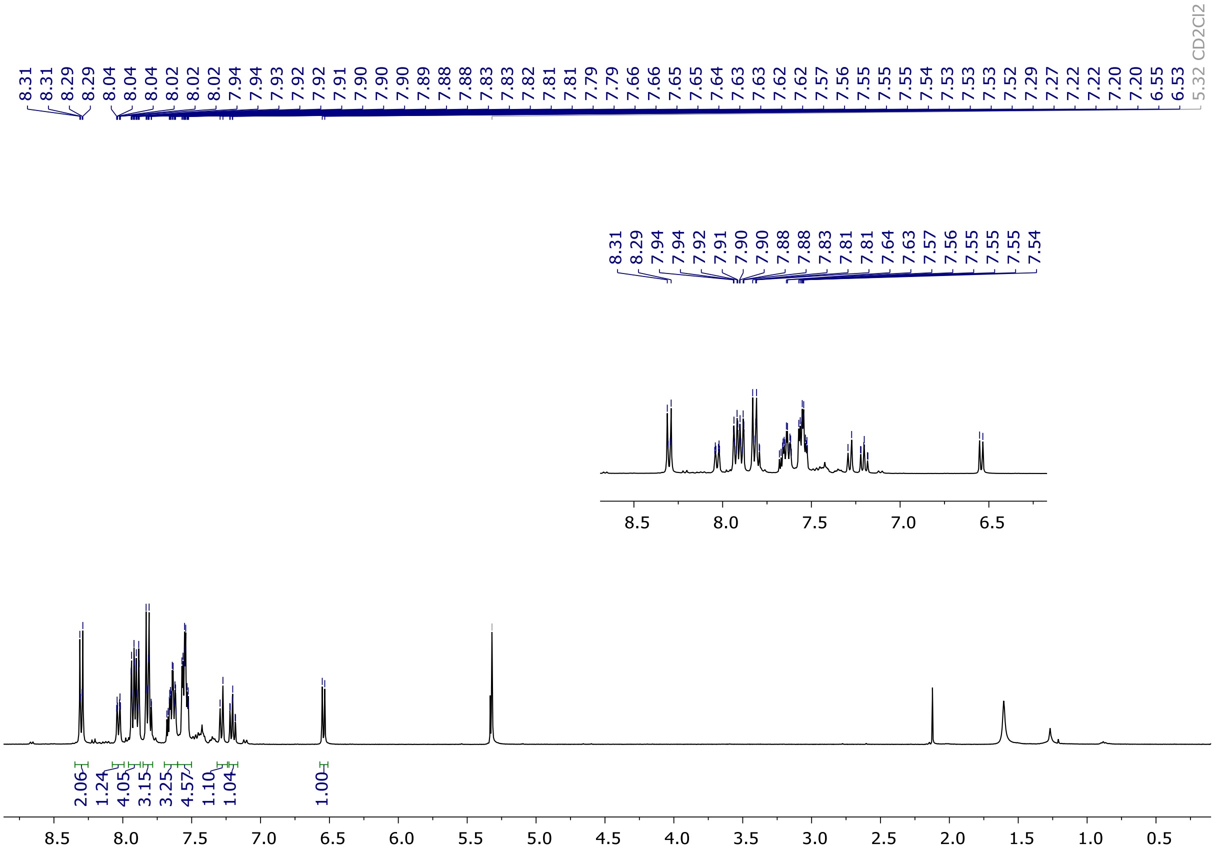

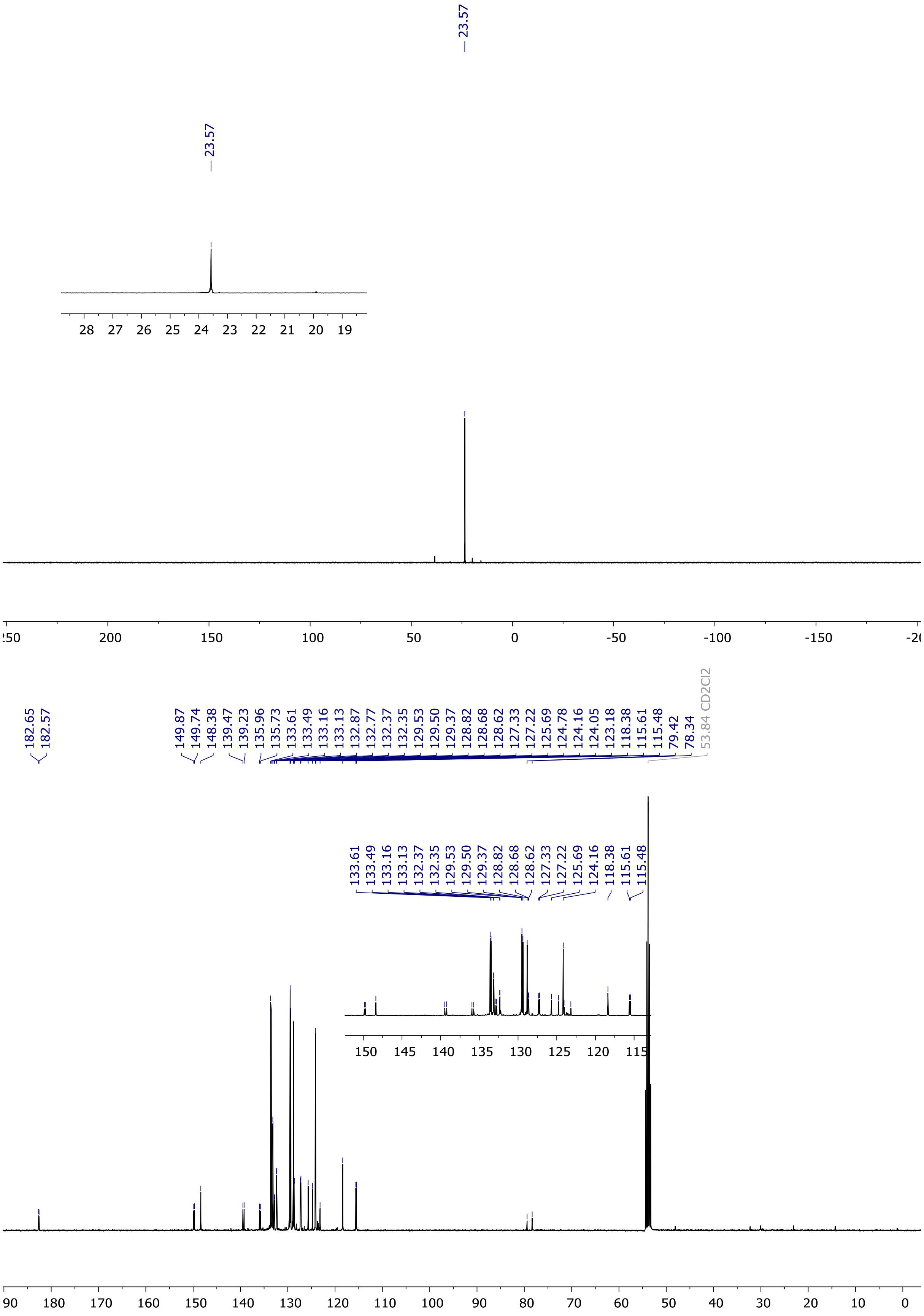

Figure S3: ^{1}H NMR (400 MHz, CD_2Cl_2, 293K), ^{13}C NMR (101 MHz, CD_2Cl_2, 293K) and $^{31}P\{^{1}H\}$ NMR (162 MHz, CD_2Cl_2, 293K) spectra of compound **5b.**

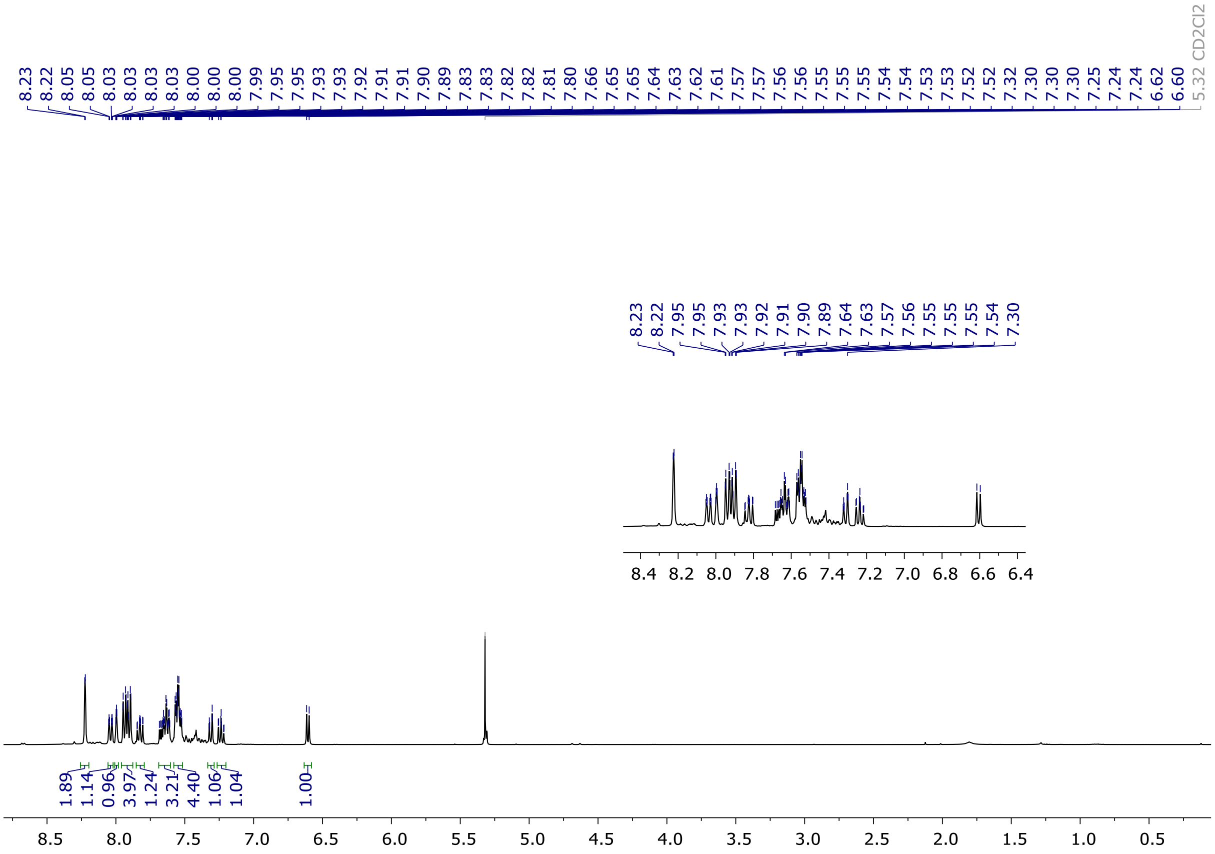
8.23 8.22 8.05 8.05 8.03 8.03 8.03 8.03 8.00 8.00 8.00 7.99 7.95 7.95 7.93 7.93 7.92 7.91 7.91 7.90 7.89 7.83 7.82 7.82 7.81 7.80 7.66 7.65 7.65 7.64 7.63 7.62 7.61 7.57 7.57 7.56 7.56 7.55 7.55 7.55 7.54 7.54 7.53 7.53 7.52 7.52 7.32 7.30 7.30 7.30 7.25 7.24 7.24 6.62 6.60
5.32 CD2Cl2
8.23 8.22 7.95 7.95 7.93 7.93 7.92 7.91 7.90 7.89 7.64 7.63 7.57 7.56 7.55 7.55 7.55 7.54 7.30
8.4 8.2 8.0 7.8 7.6 7.4 7.2 7.0 6.8 6.6 6.4
1.89 1.14 0.96 3.97 1.24 3.21 4.40 1.06 1.04 1.00
8.5 8.0 7.5 7.0 6.5 6.0 5.5 5.0 4.5 4.0 3.5 3.0 2.5 2.0 1.5 1.0 0.5

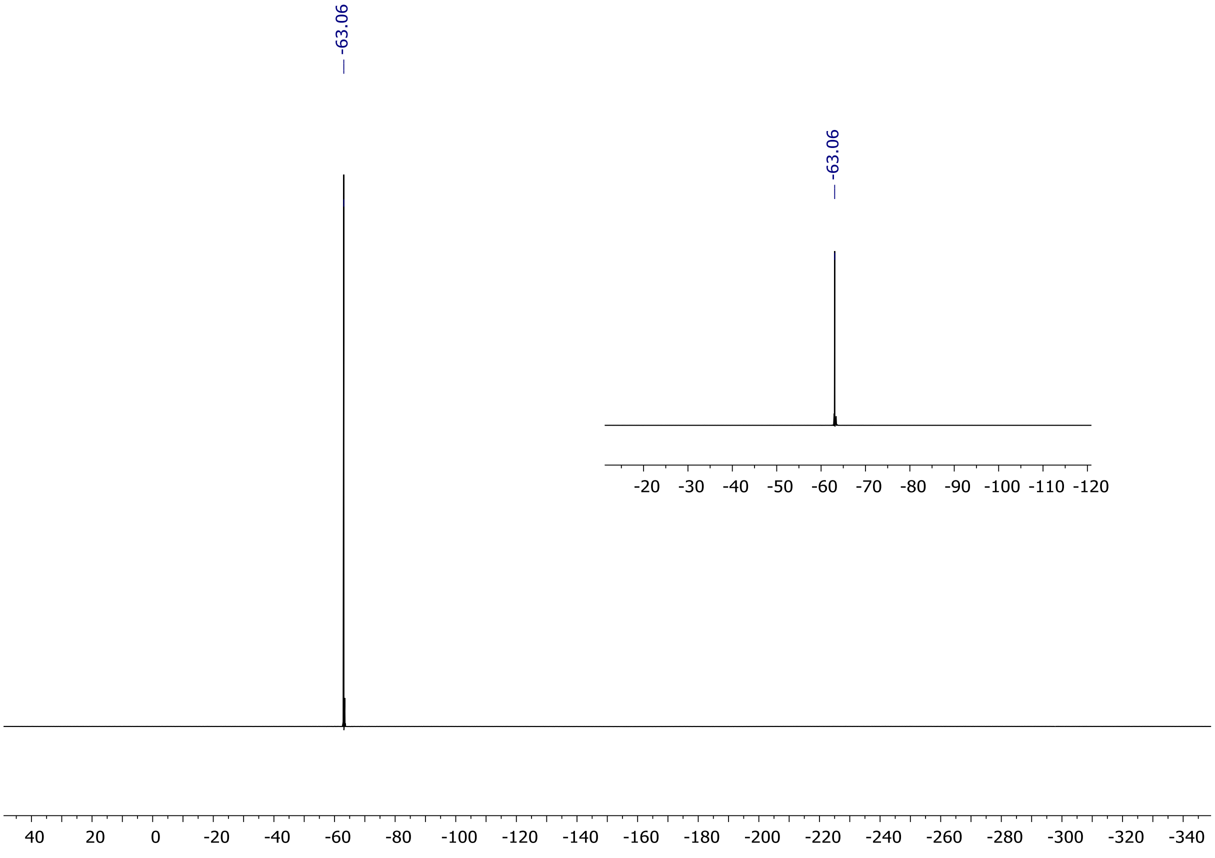
-63.06
-63.06
-20 -30 -40 -50 -60 -70 -80 -90 -100 -110 -120
40 20 0 -20 -40 -60 -80 -100 -120 -140 -160 -180 -200 -220 -240 -260 -280 -300 -320 -340

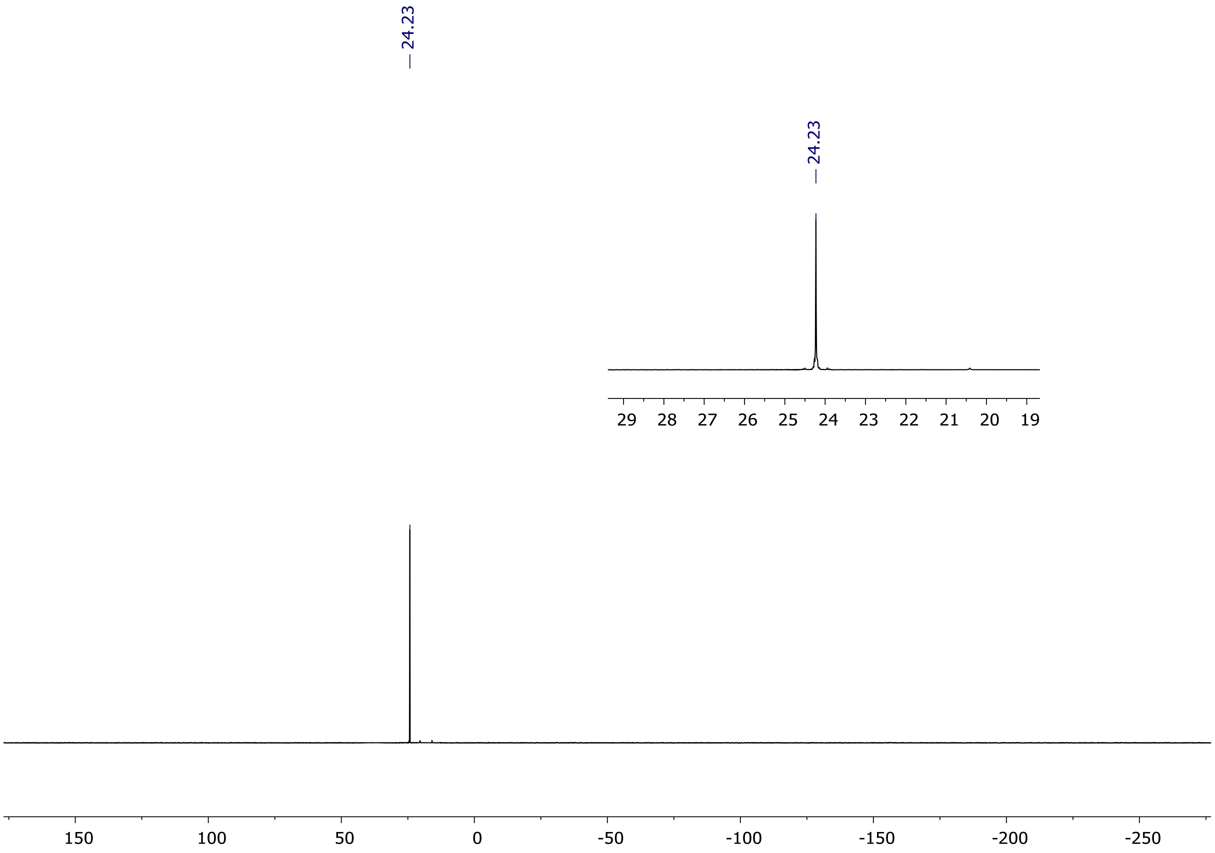

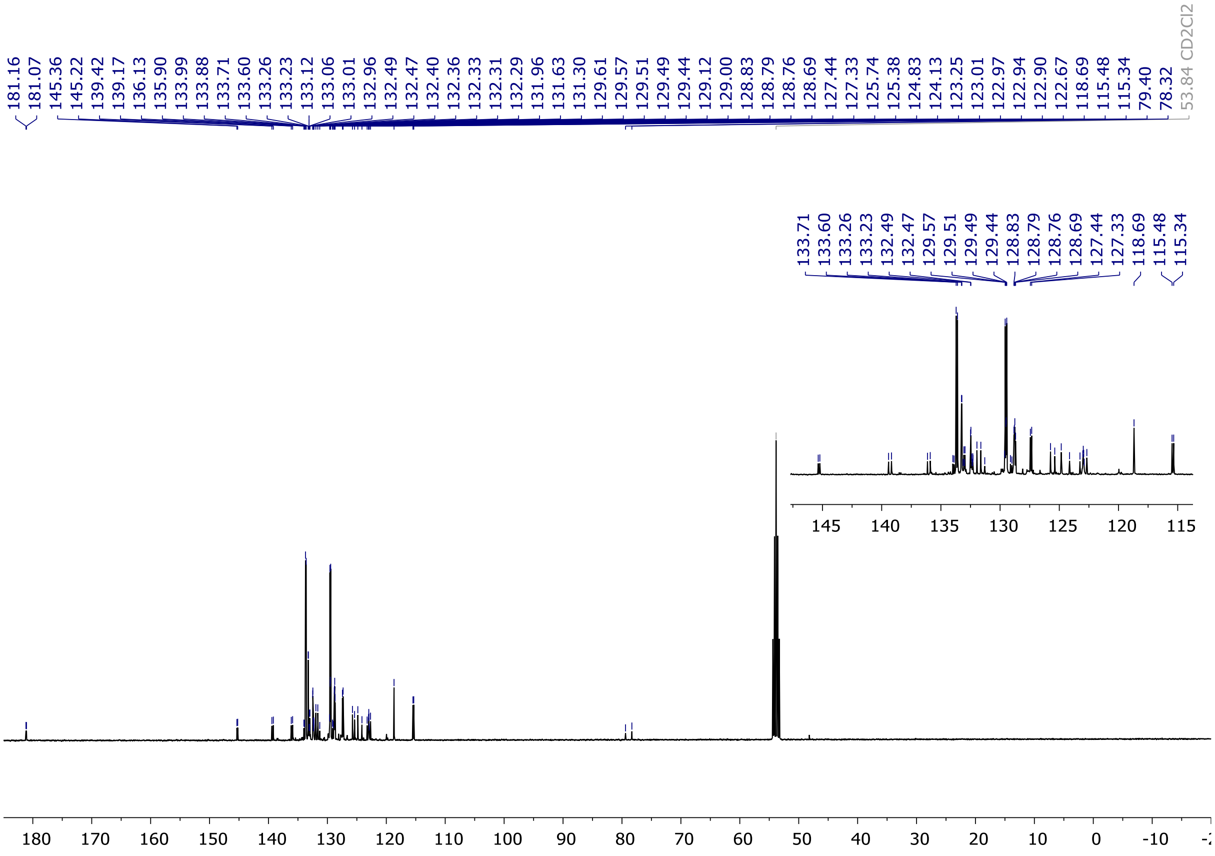

Figure S4: ^{1}H NMR (400 MHz, CD_2Cl_2, 293K), ^{13}C NMR (101 MHz, CD_2Cl_2, 293K), ^{19}F NMR (376 MHz, CD_2Cl_2 and $^{31}P\{^{1}H\}$ NMR (162 MHz, CD_2Cl_2, 293K) spectra of compound **5c.**

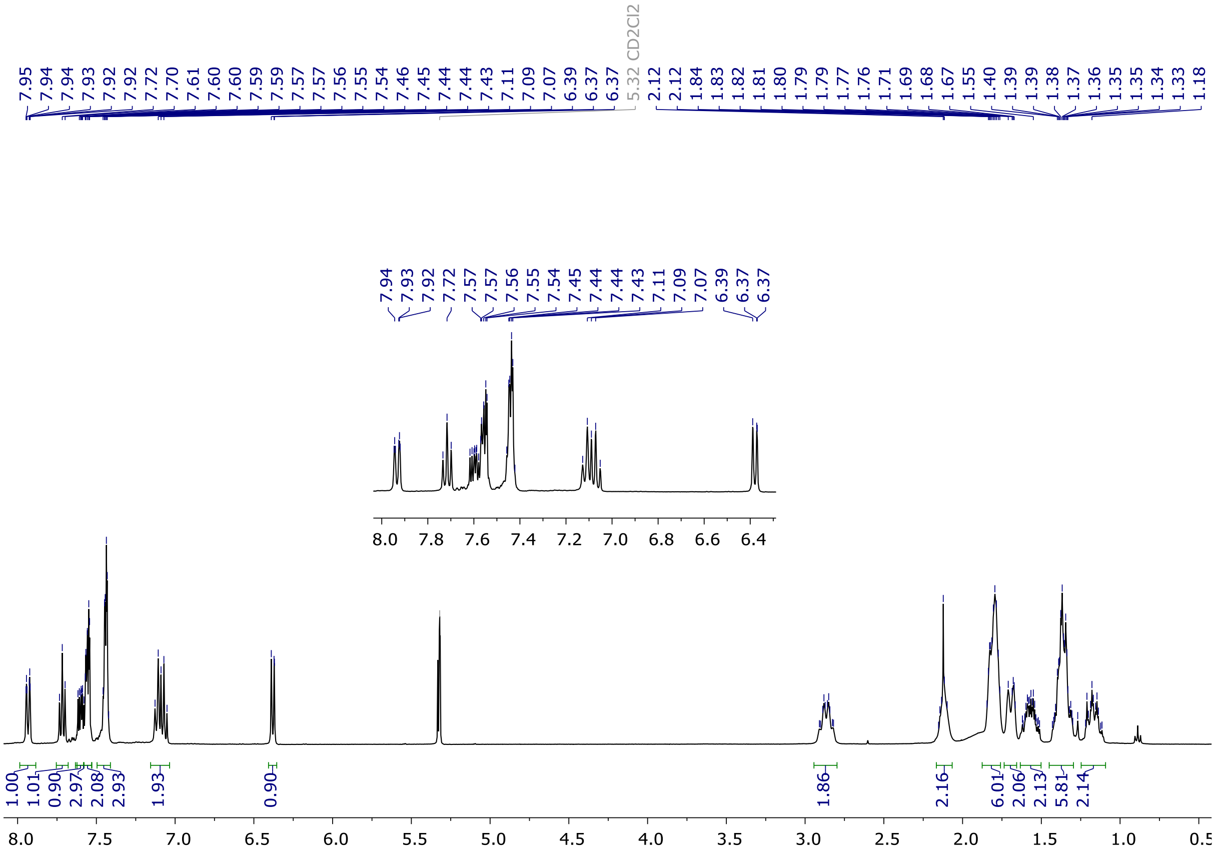
7.95
7.94
7.94
7.93
7.92
7.92
7.72
7.70
7.61
7.60
7.60
7.59
7.59
7.57
7.57
7.56
7.55
7.54
7.46
7.45
7.44
7.44
7.43
7.11
7.09
7.07
6.39
6.37
6.37
5.32 CD2Cl2
2.12
2.12
1.84
1.83
1.82
1.81
1.80
1.79
1.77
1.76
1.71
1.69
1.68
1.67
1.55
1.40
1.39
1.39
1.38
1.37
1.36
1.35
1.35
1.34
1.33
1.18
1.00
1.01
0.90
2.97
2.08
2.93
1.93
0.90
1.86
2.16
6.01
2.06
2.13
5.81
2.14

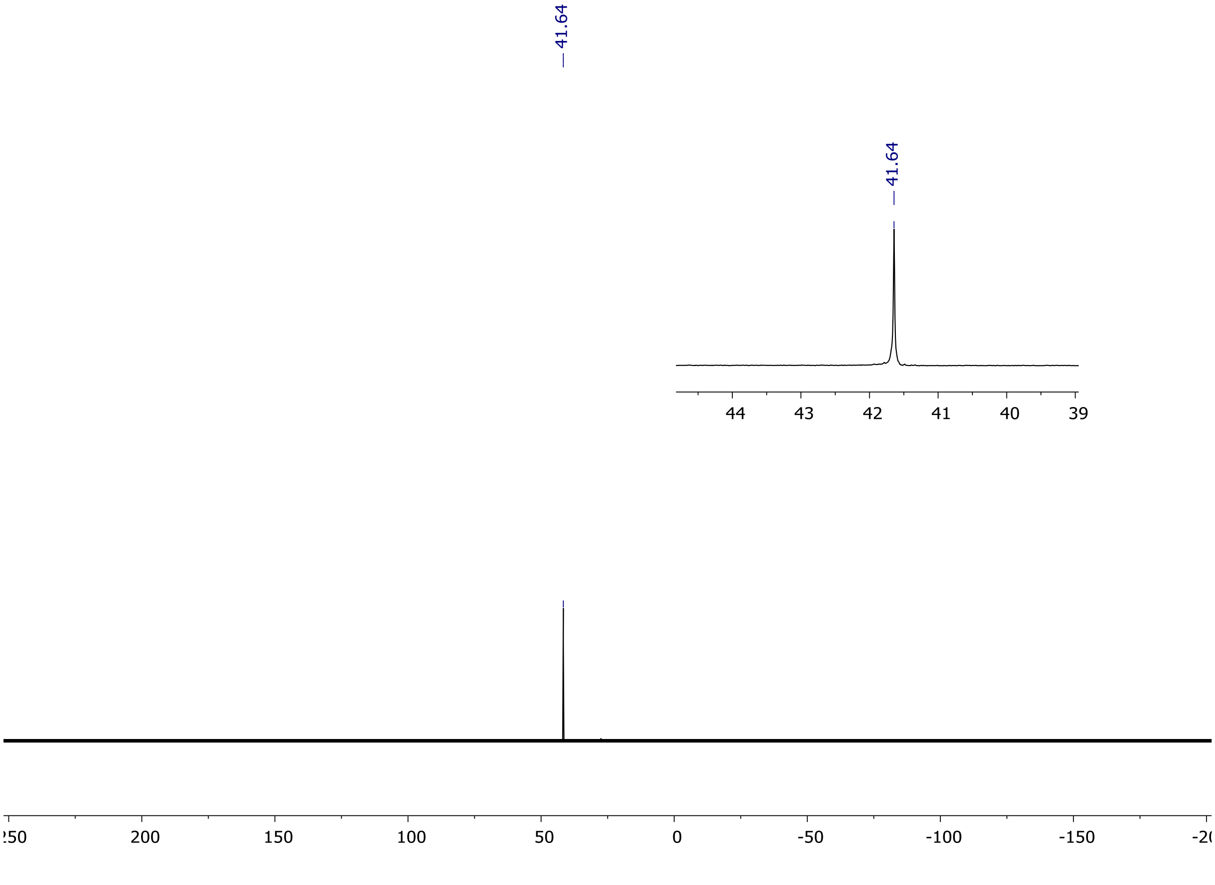
41.64
41.64

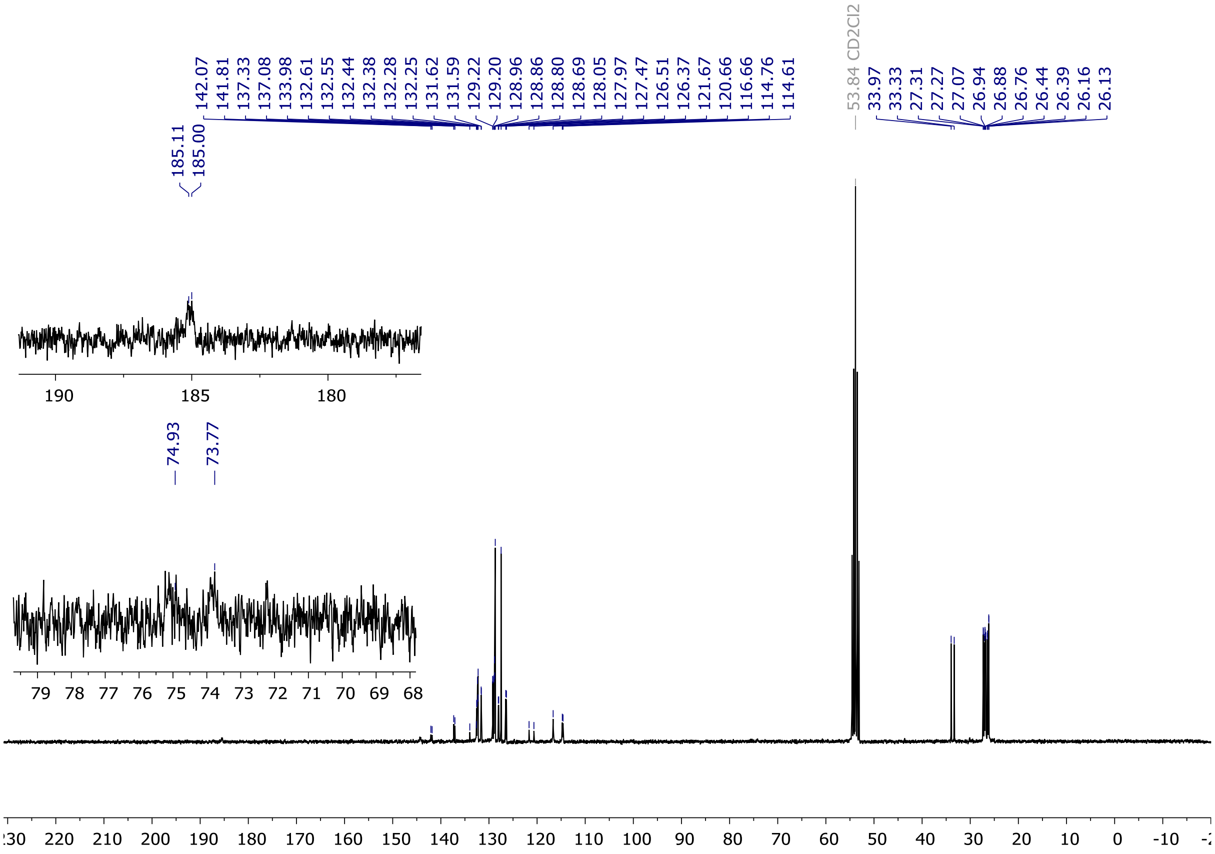

Figure S5: ^{1}H NMR (400 MHz, CD_2Cl_2, 293K), ^{13}C NMR (75 MHz, CD_2Cl_2, 293K) and $^{31}P\{^{1}H\}$ NMR (162 MHz, CD_2Cl_2, 293K) spectra of compound **5f.**

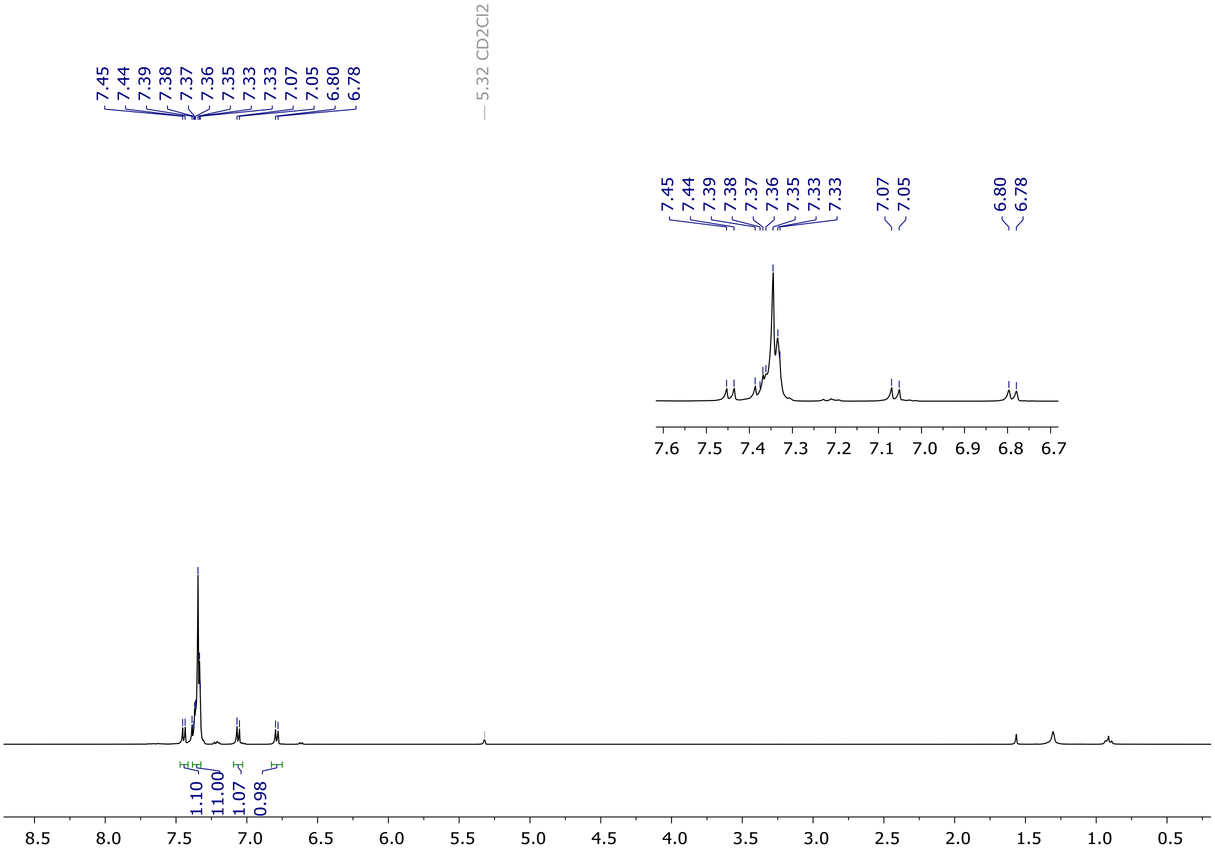

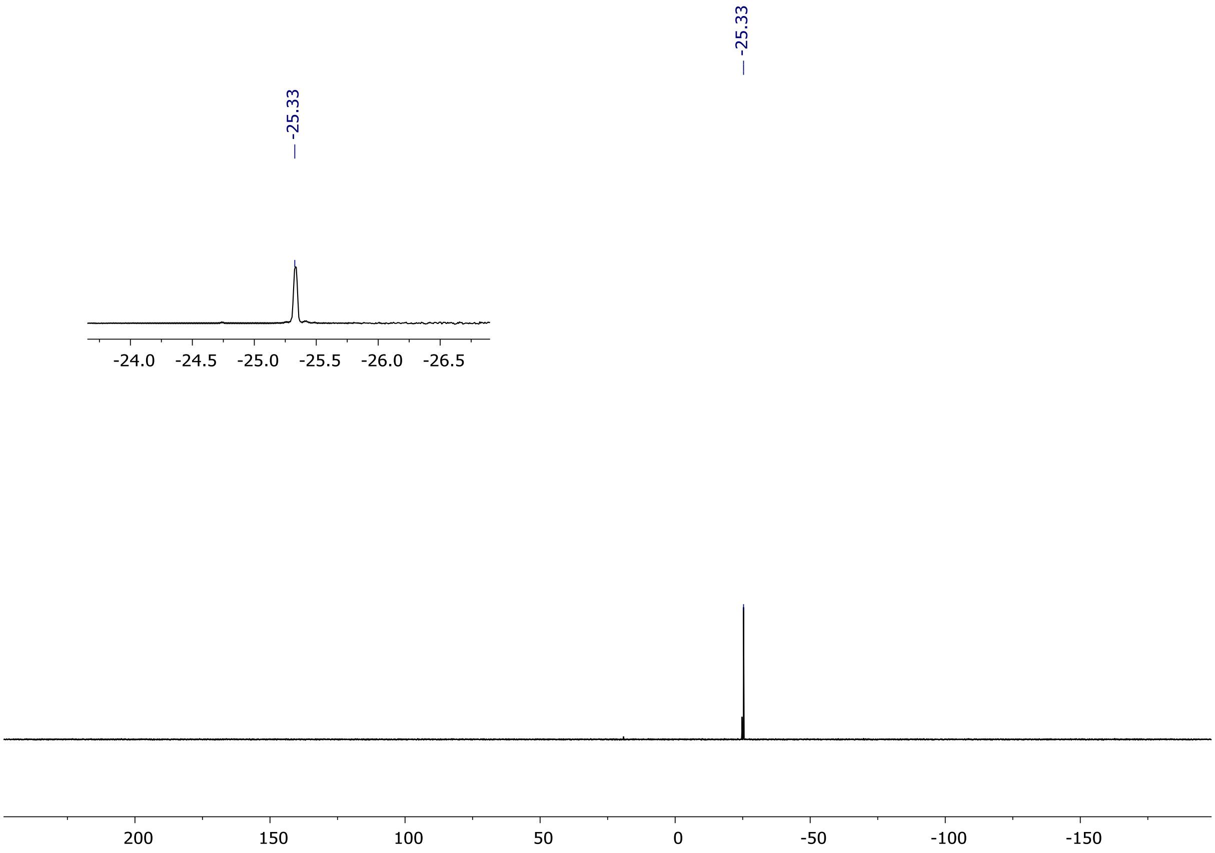

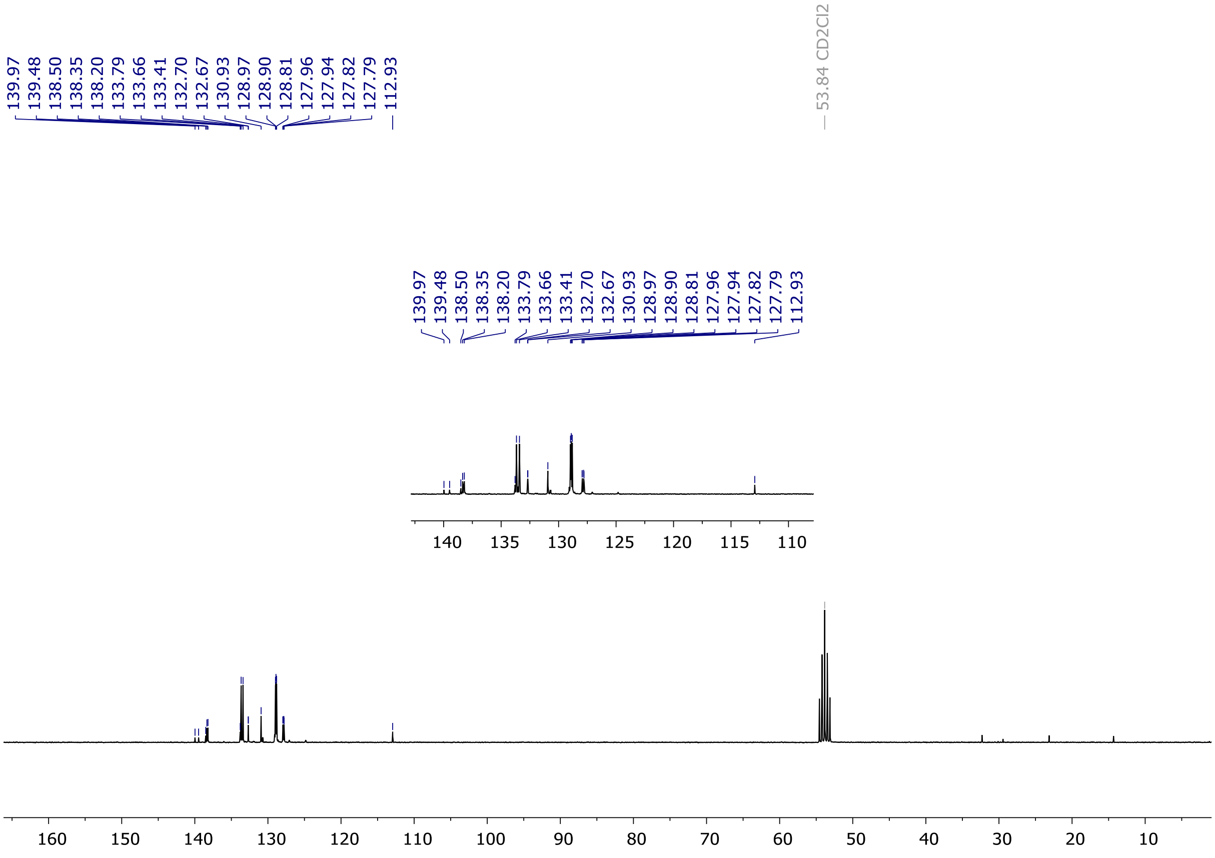

Figure S6: ^{1}H NMR (300 MHz, CD_2Cl_2, 293K), ^{13}C NMR (75 MHz, CD_2Cl_2, 293K) and ^{31}P{^{1}H} NMR (121 MHz, CD_2Cl_2, 293K) spectra of compound **8.**

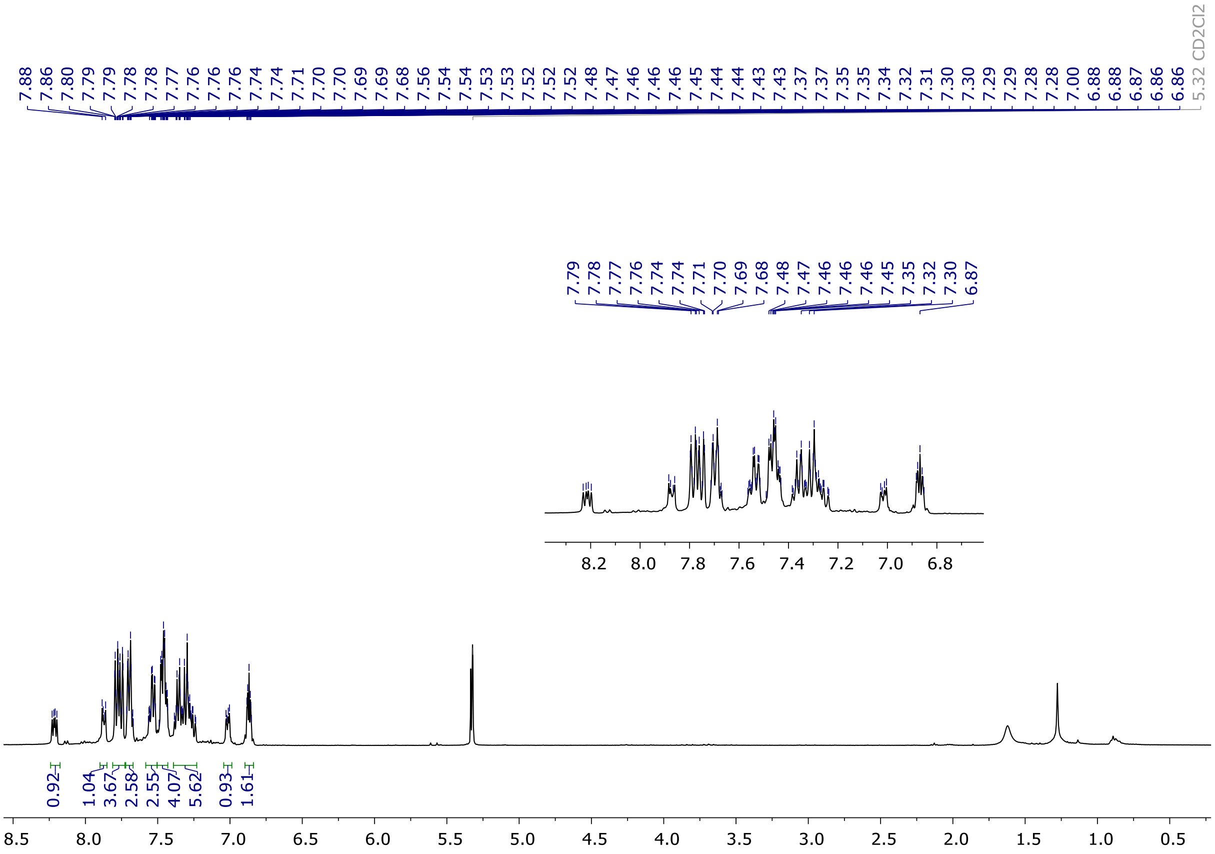

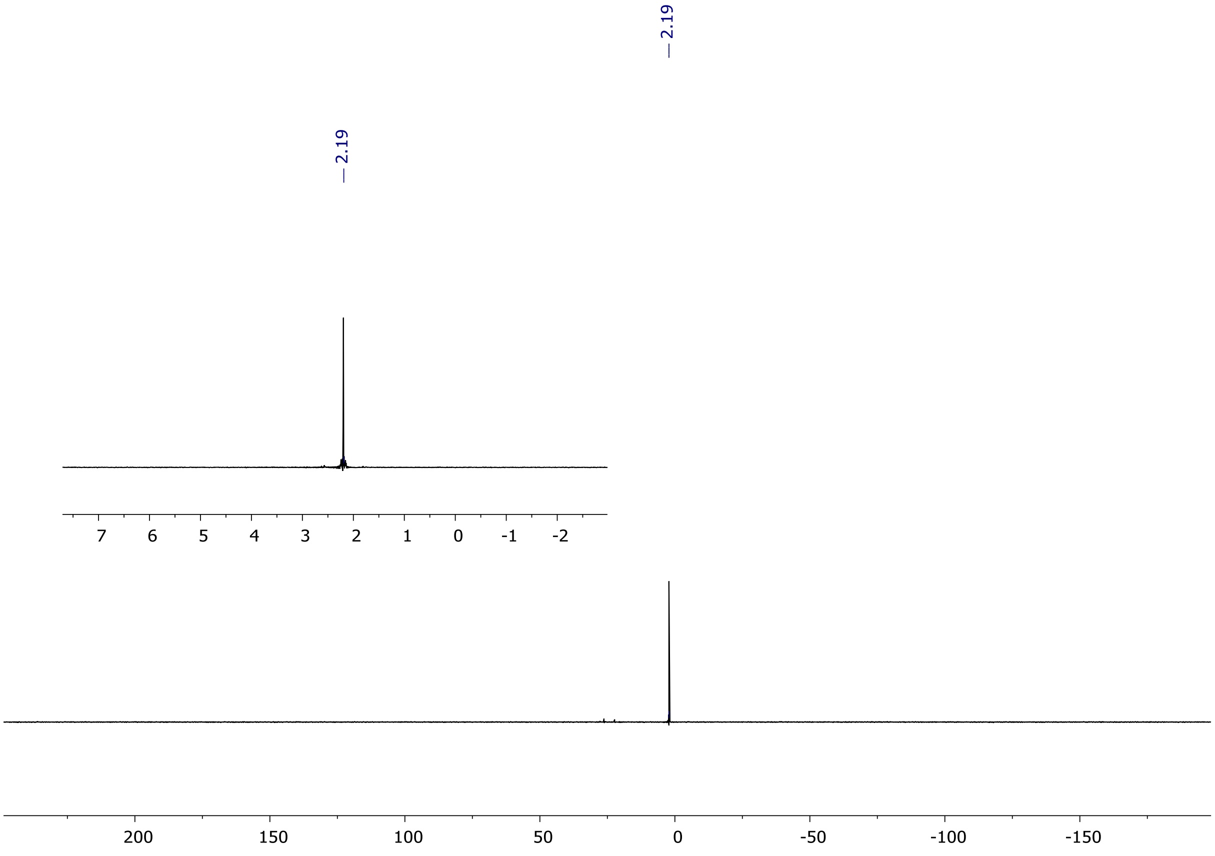

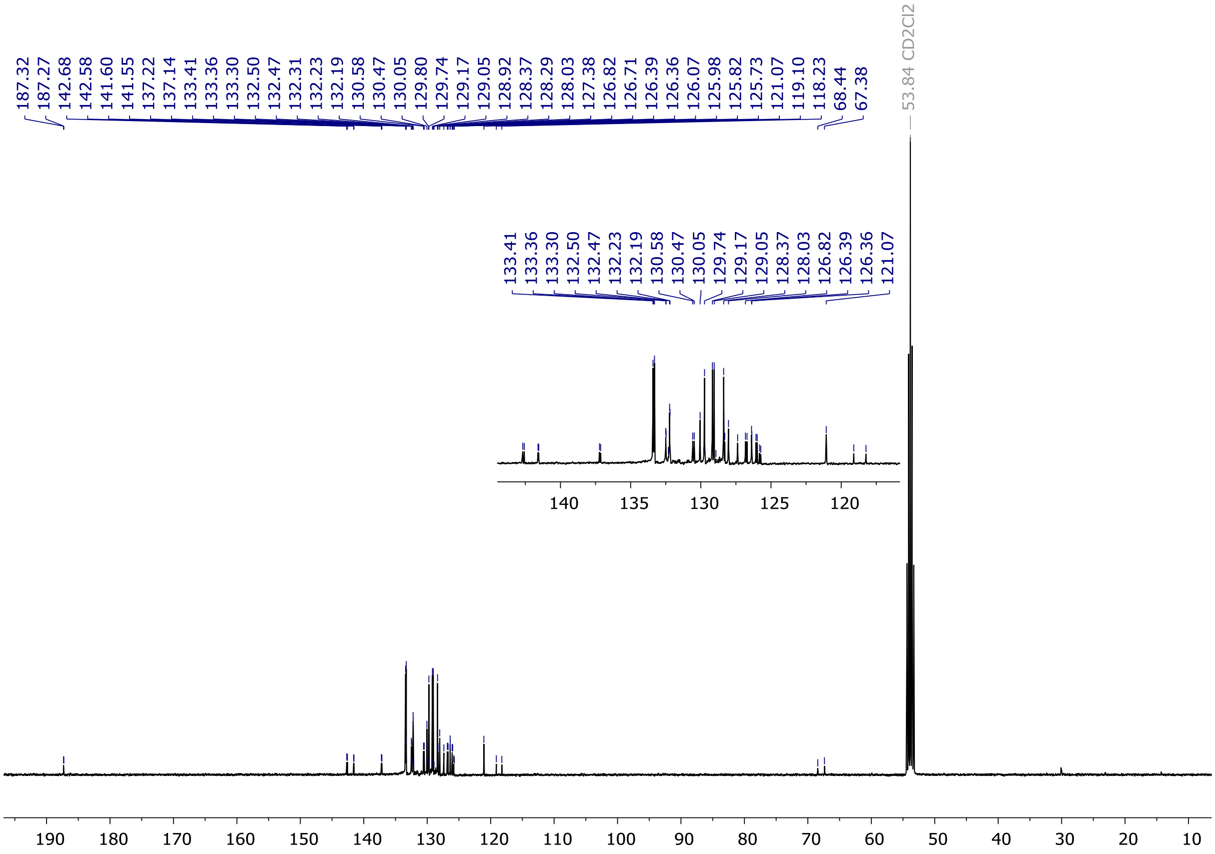

Figure S7: ^{1}H NMR (400 MHz, CD_2Cl_2, 293K), ^{13}C NMR (101 MHz, CD_2Cl_2, 293K) and ^{31}P{^{1}H} NMR (121 MHz, CD_2Cl_2, 293K) spectra of compound **7a.**

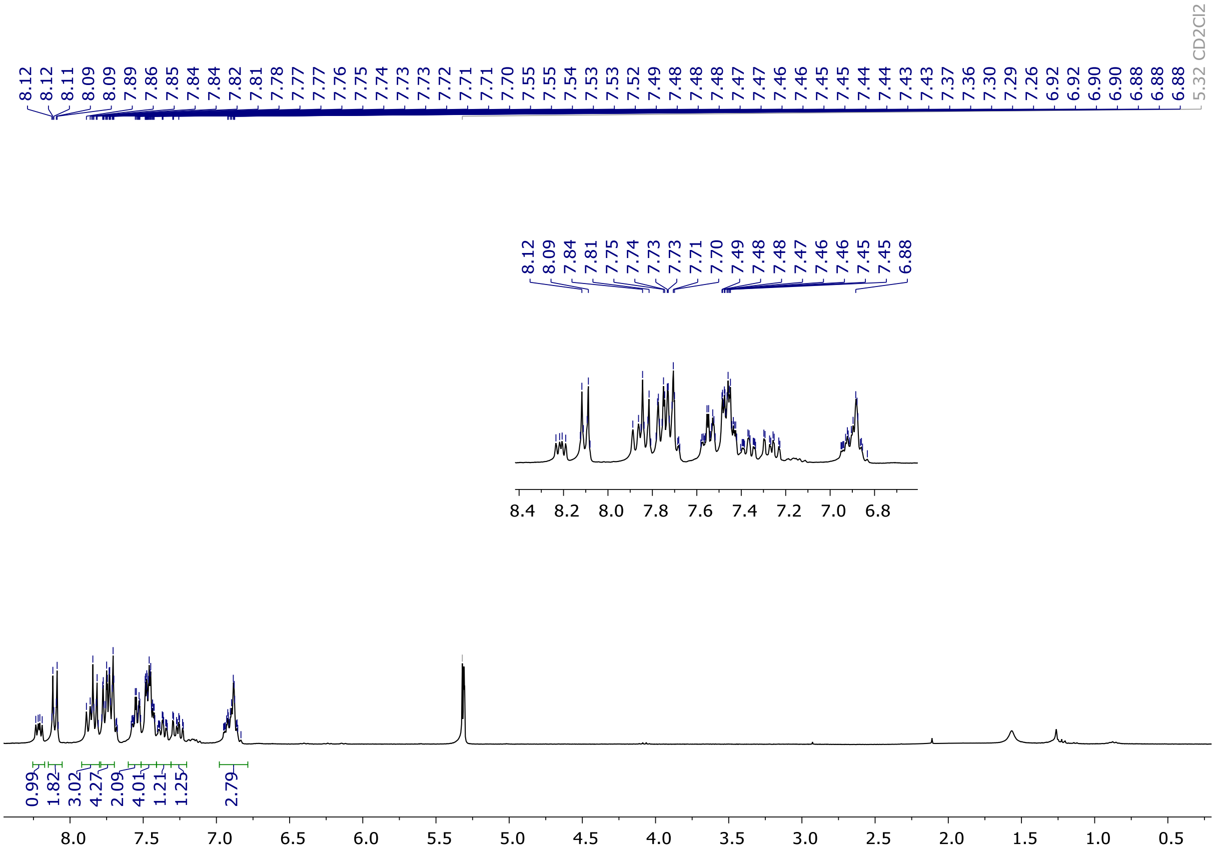

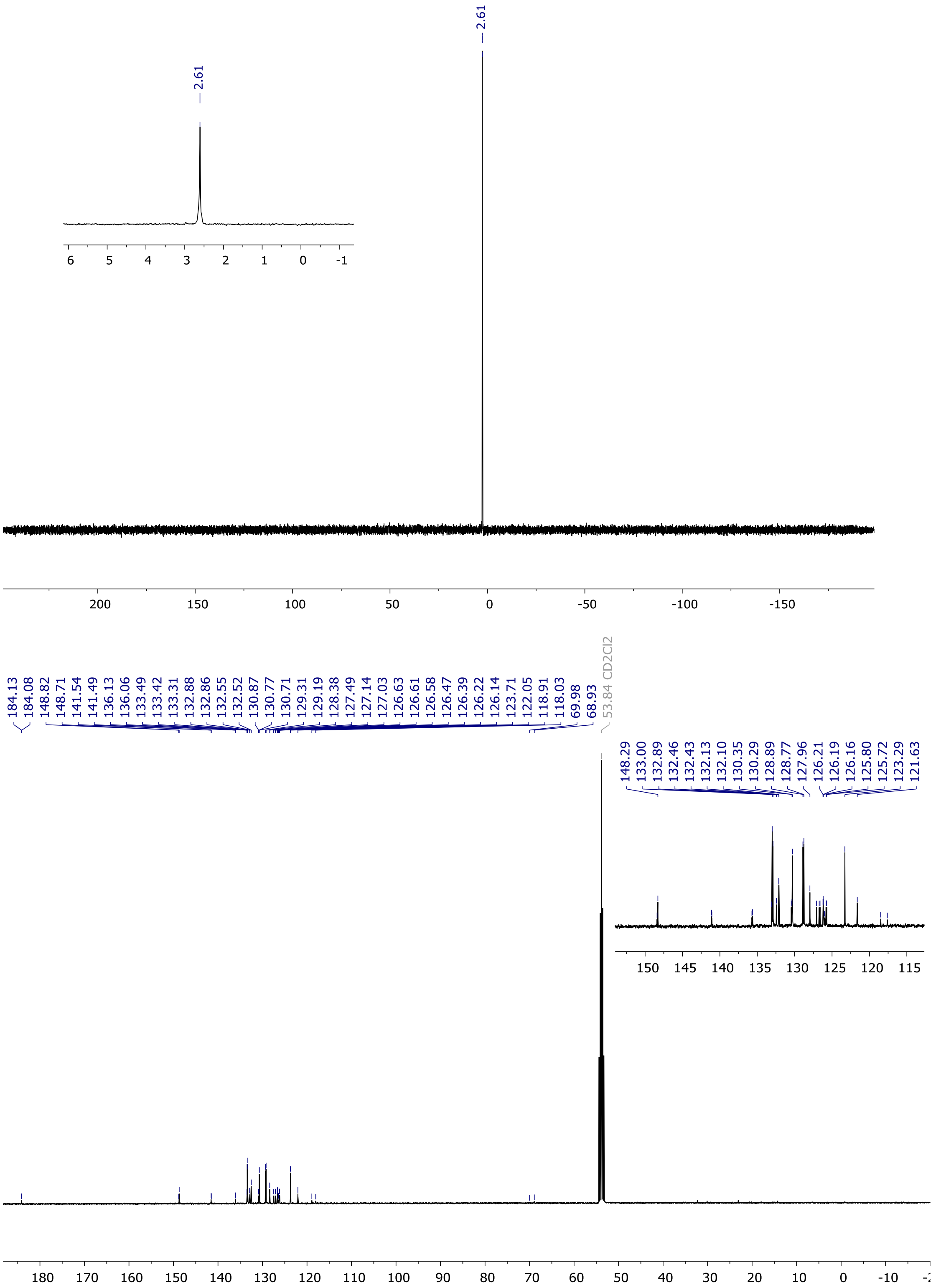

Figure S8: ^{1}H NMR (300 MHz, CD_2Cl_2, 293K), ^{13}C NMR (101 MHz, CD_2Cl_2, 293K) and $^{31}P\{^{1}H\}$ NMR (121 MHz, CD_2Cl_2, 293K) spectra of compound **7b.**

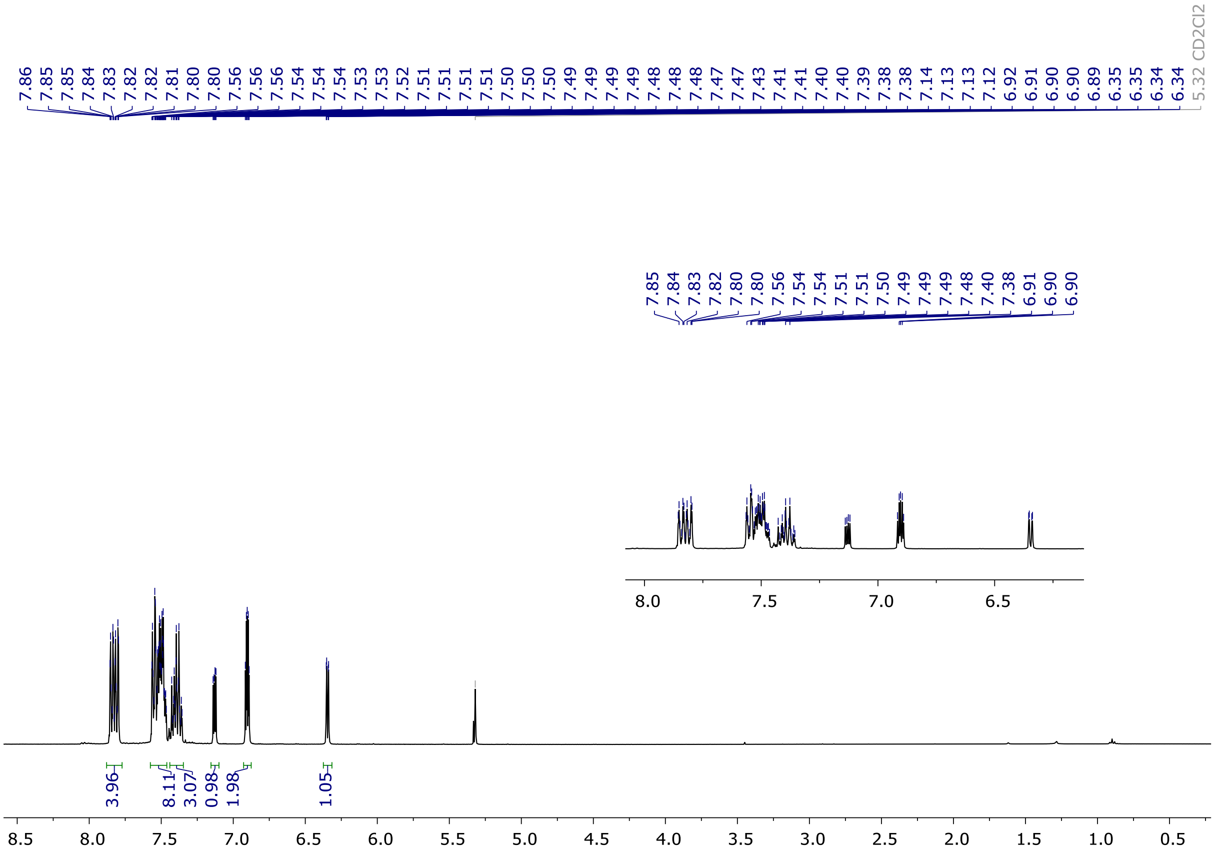
7.86 7.85 7.85 7.84 7.83 7.82 7.82 7.81 7.80 7.80 7.56 7.56 7.56 7.54 7.54 7.54 7.53 7.52 7.51 7.51 7.51 7.51 7.50 7.50 7.50 7.49 7.49 7.49 7.49 7.48 7.48 7.48 7.47 7.47 7.43 7.41 7.41 7.40 7.40 7.39 7.38 7.38 7.14 7.13 7.13 7.12 6.92 6.91 6.90 6.90 6.89 6.35 6.35 6.34 6.34
5.32 CD2Cl2
7.85 7.84 7.83 7.82 7.80 7.80 7.56 7.54 7.54 7.51 7.51 7.50 7.49 7.49 7.49 7.48 7.40 7.38 6.91 6.90 6.90
8.0 7.5 7.0 6.5
3.96 8.11 3.07 0.98 1.98 1.05
8.5 8.0 7.5 7.0 6.5 6.0 5.5 5.0 4.5 4.0 3.5 3.0 2.5 2.0 1.5 1.0 0.5

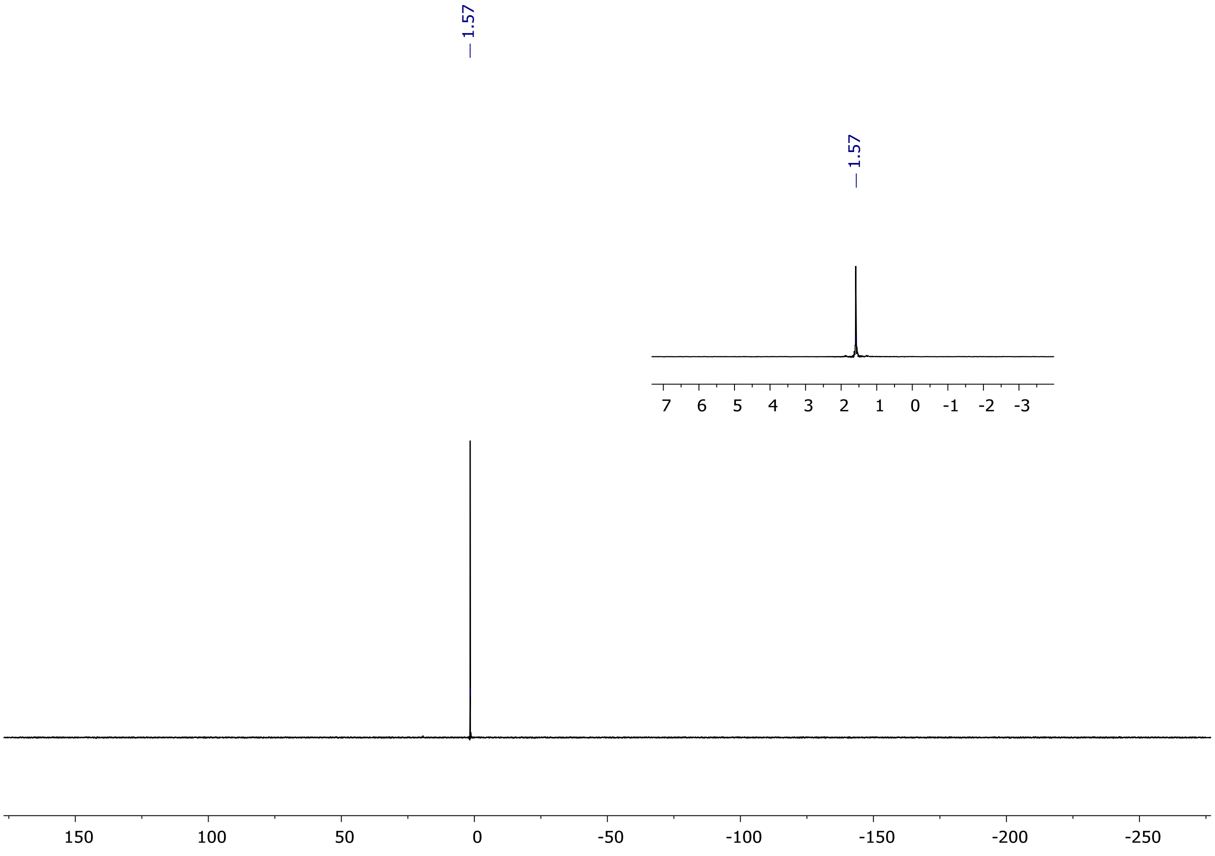
1.57
1.57
7 6 5 4 3 2 1 0 -1 -2 -3
150 100 50 0 -50 -100 -150 -200 -250

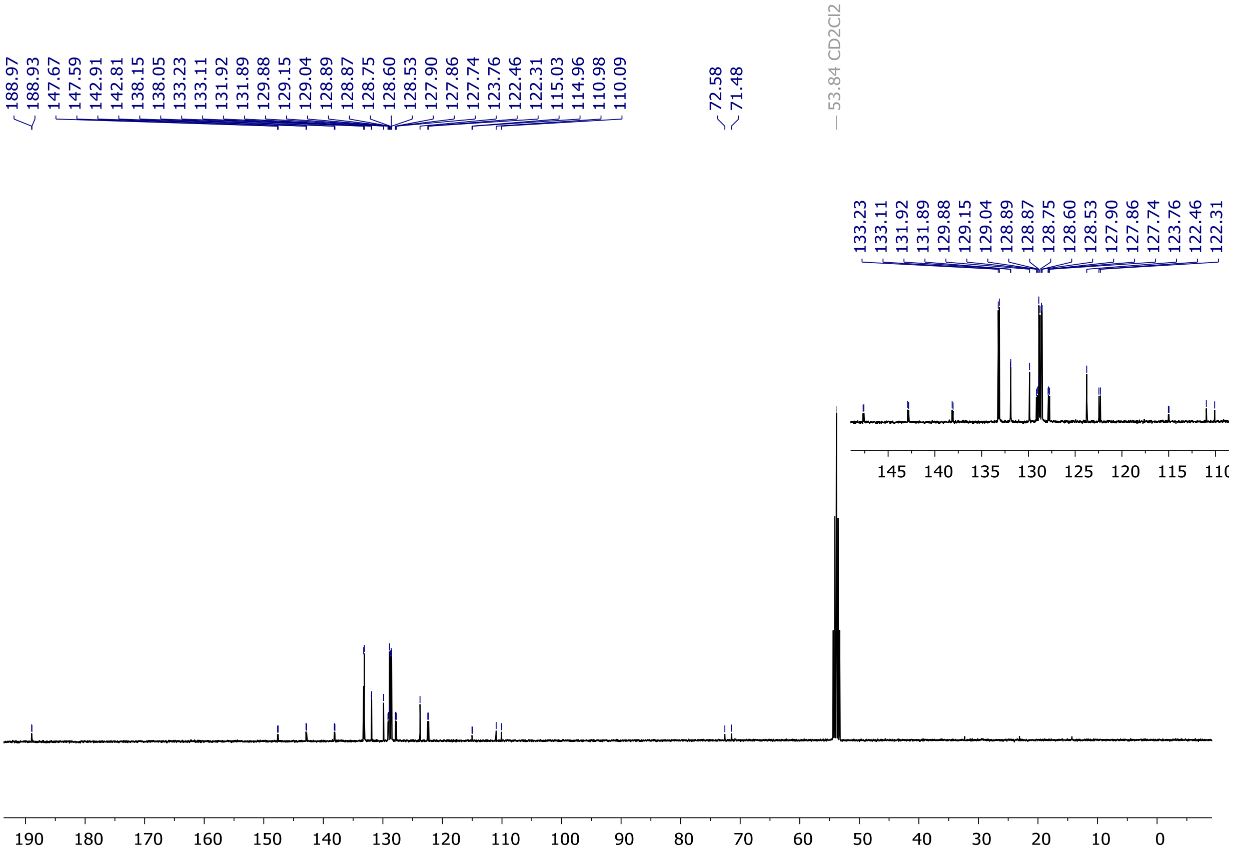

Figure S10: ^{1}H NMR (400 MHz, CD_2Cl_2, 293K), ^{13}C NMR (101 MHz, CD_2Cl_2, 293K) and $^{31}P\{^{1}H\}$ NMR (162 MHz, CD_2Cl_2, 293K) spectra of compound **9.**

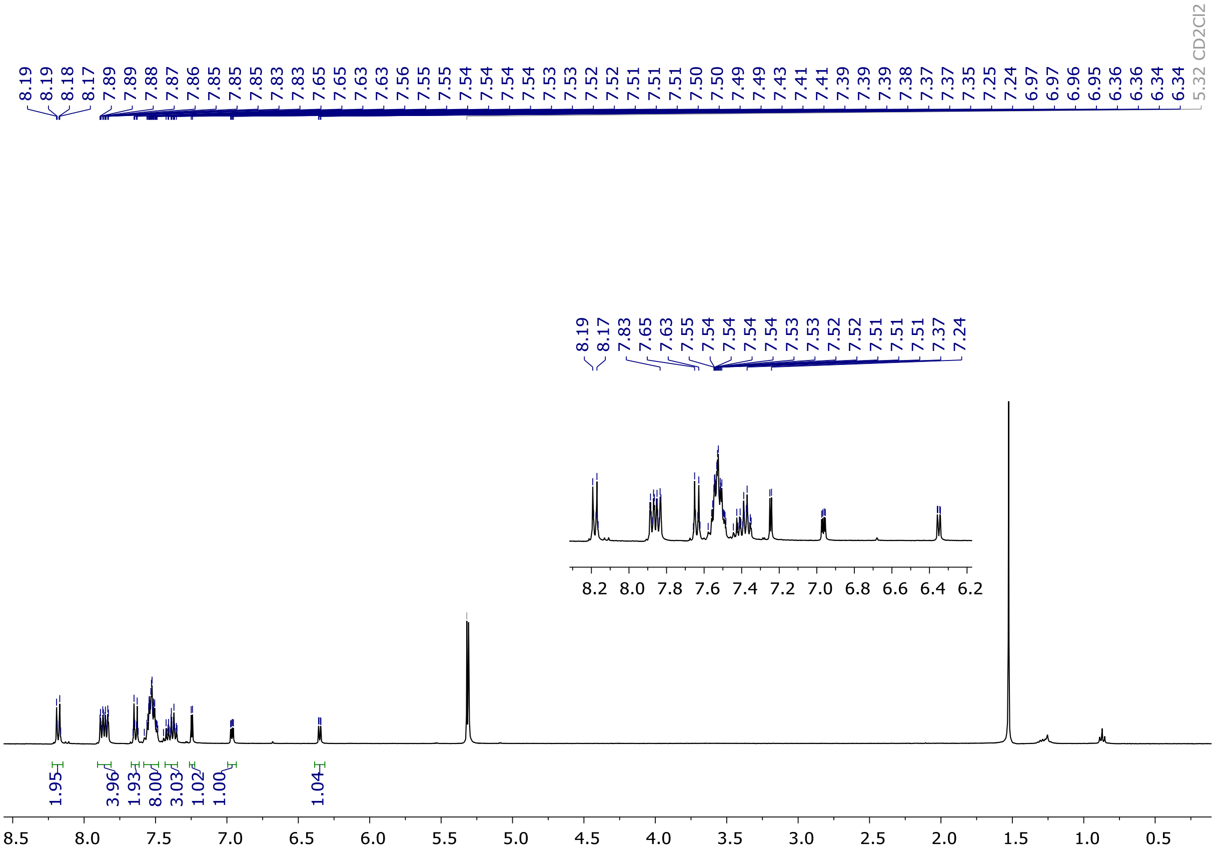

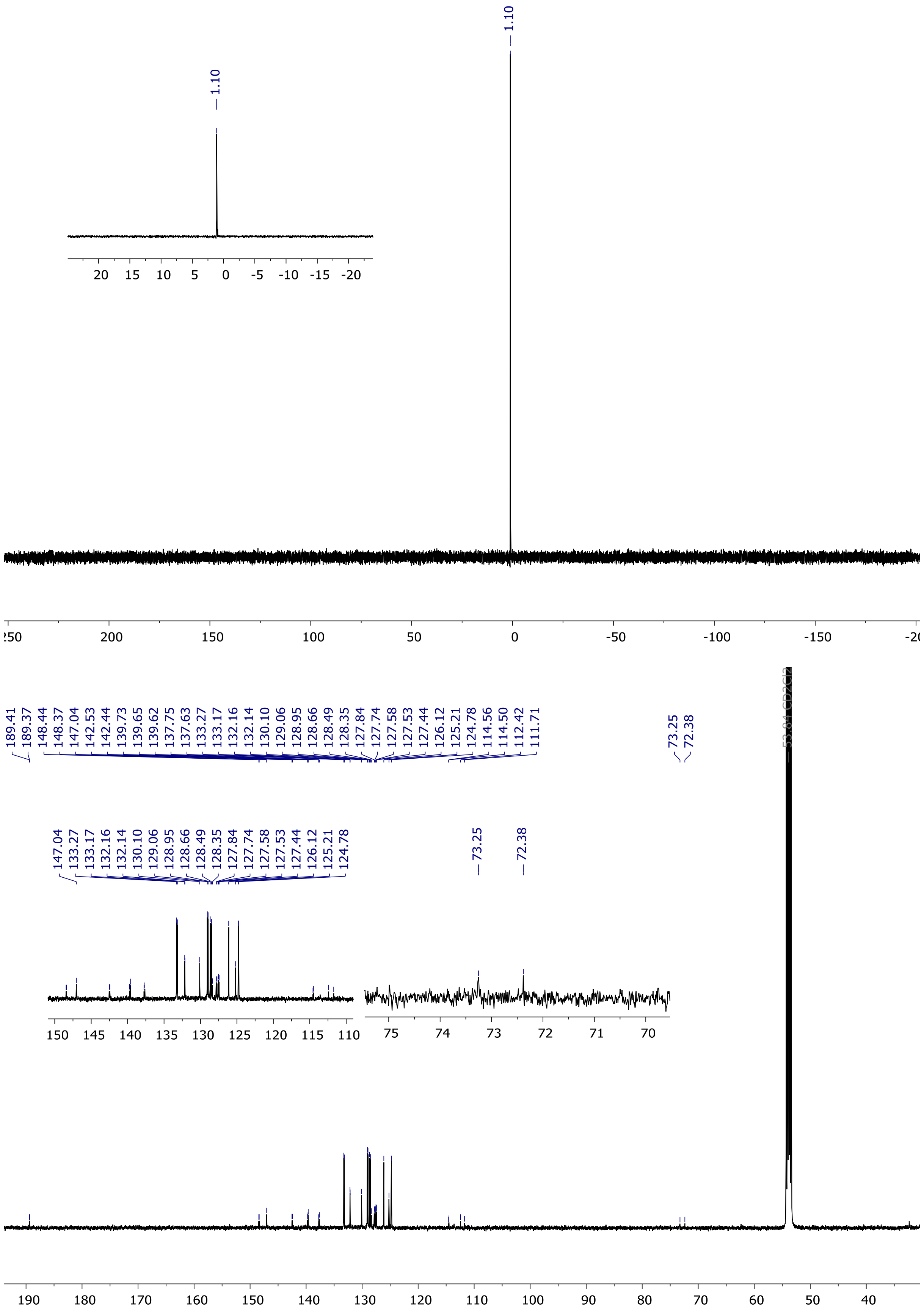

Figure S11: ^{1}H NMR (400 MHz, CD_2Cl_2, 293K), ^{13}C NMR (126 MHz, CD_2Cl_2, 293K) and $^{31}P\{^{1}H\}$ NMR (162 MHz, CD_2Cl_2, 293K) spectra of **10’.**

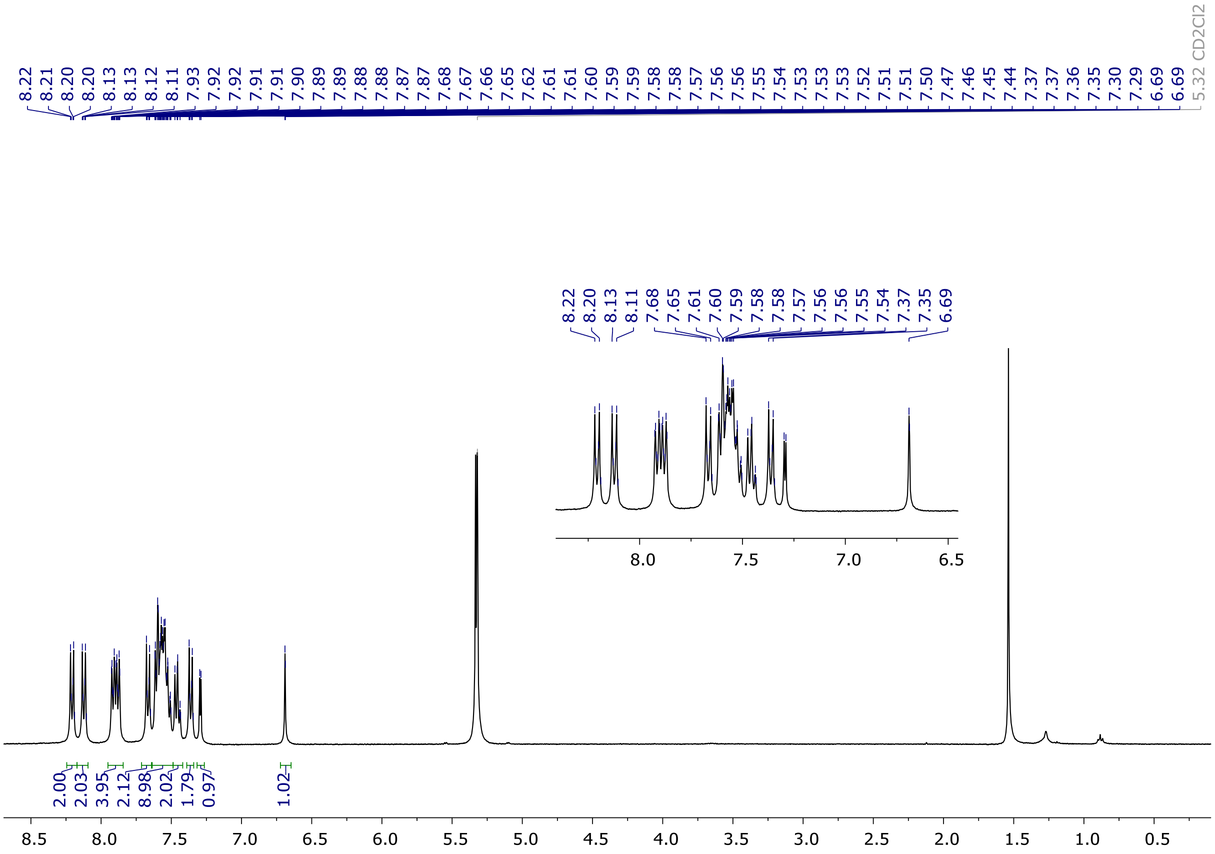
8.22
8.21
8.20
8.20
8.13
8.13
8.12
8.11
7.93
7.92
7.92
7.91
7.91
7.90
7.89
7.89
7.88
7.88
7.87
7.87
7.68
7.67
7.66
7.65
7.62
7.61
7.61
7.60
7.59
7.59
7.58
7.58
7.57
7.56
7.56
7.55
7.54
7.53
7.53
7.53
7.52
7.51
7.51
7.50
7.47
7.46
7.45
7.44
7.37
7.37
7.36
7.35
7.30
7.29
6.69
6.69
5.32 CD2Cl2
2.00
2.03
3.95
2.12
8.98
2.02
1.79
0.97
1.02
8.5 8.0 7.5 7.0 6.5 6.0 5.5 5.0 4.5 4.0 3.5 3.0 2.5 2.0 1.5 1.0 0.5

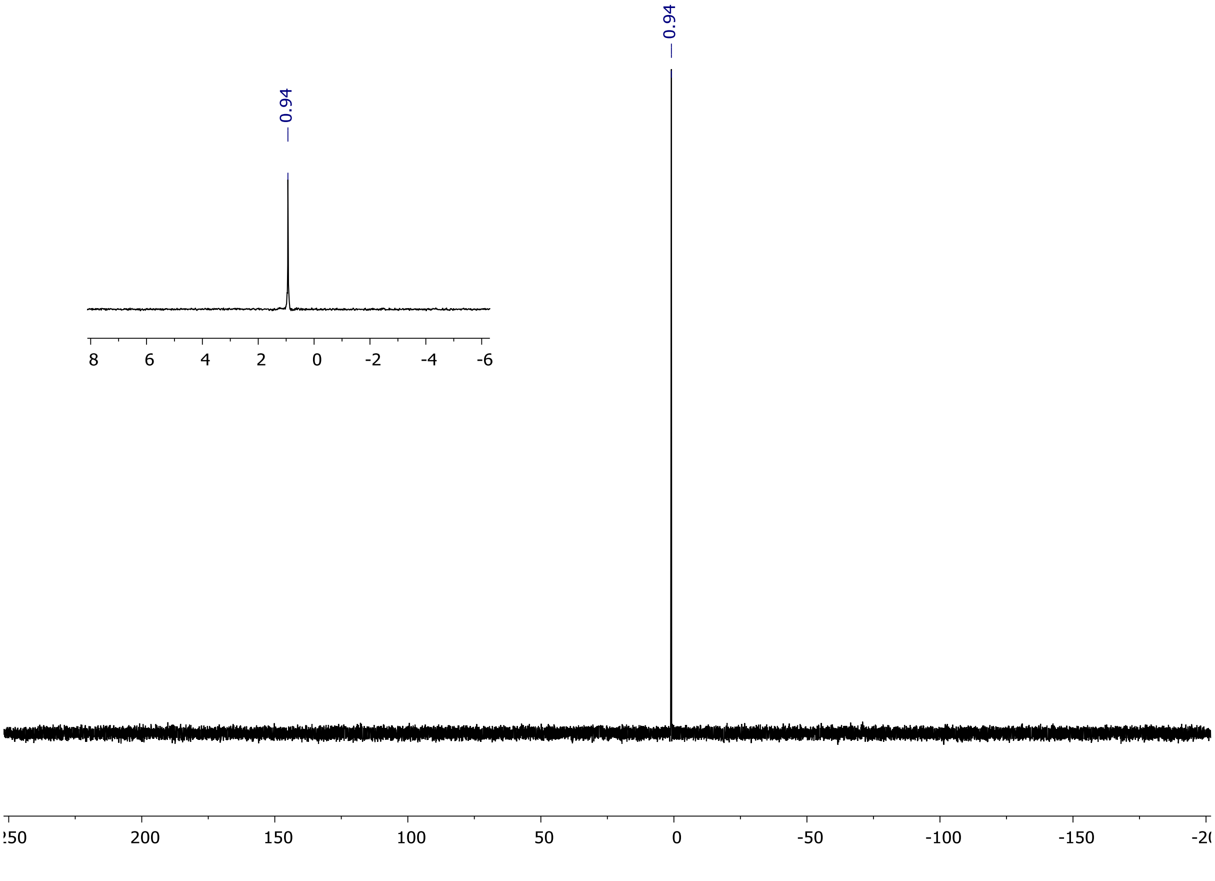
0.94
0.94
8 6 4 2 0 -2 -4 -6
250 200 150 100 50 0 -50 -100 -150 -200

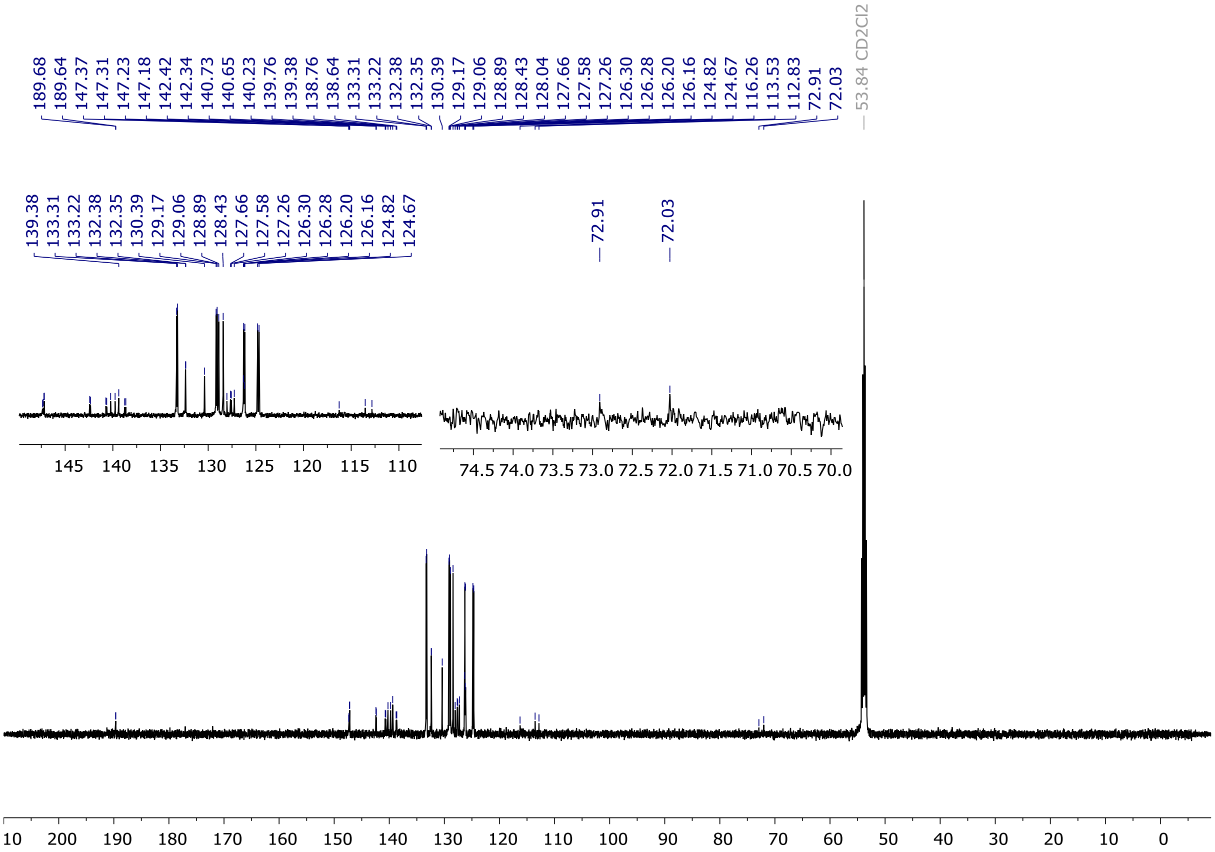

Figure S12: ^{1}H NMR (400 MHz, CD_2Cl_2, 293K), ^{13}C NMR (126 MHz, CD_2Cl_2, 293K) and $^{31}P\{^{1}H\}$ NMR (162 MHz, CD_2Cl_2, 293K) spectra of **10.**

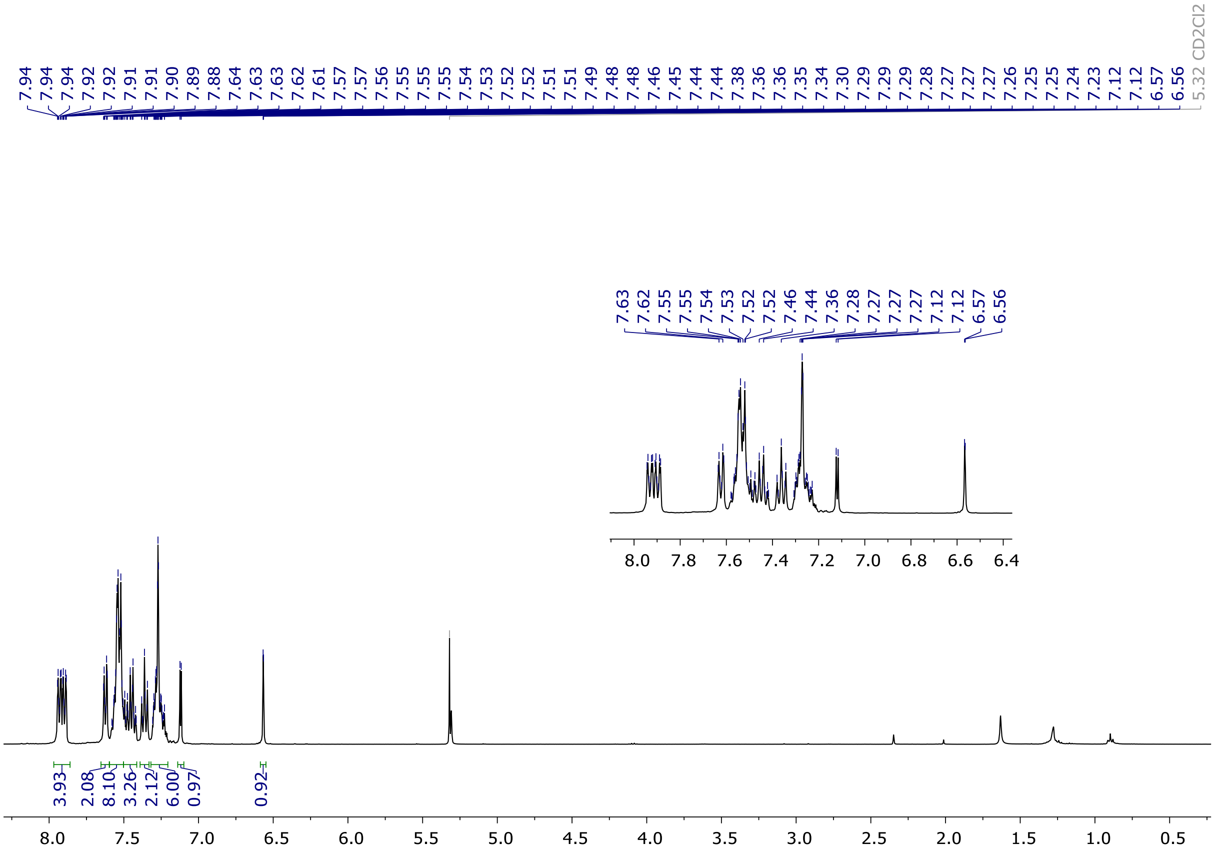

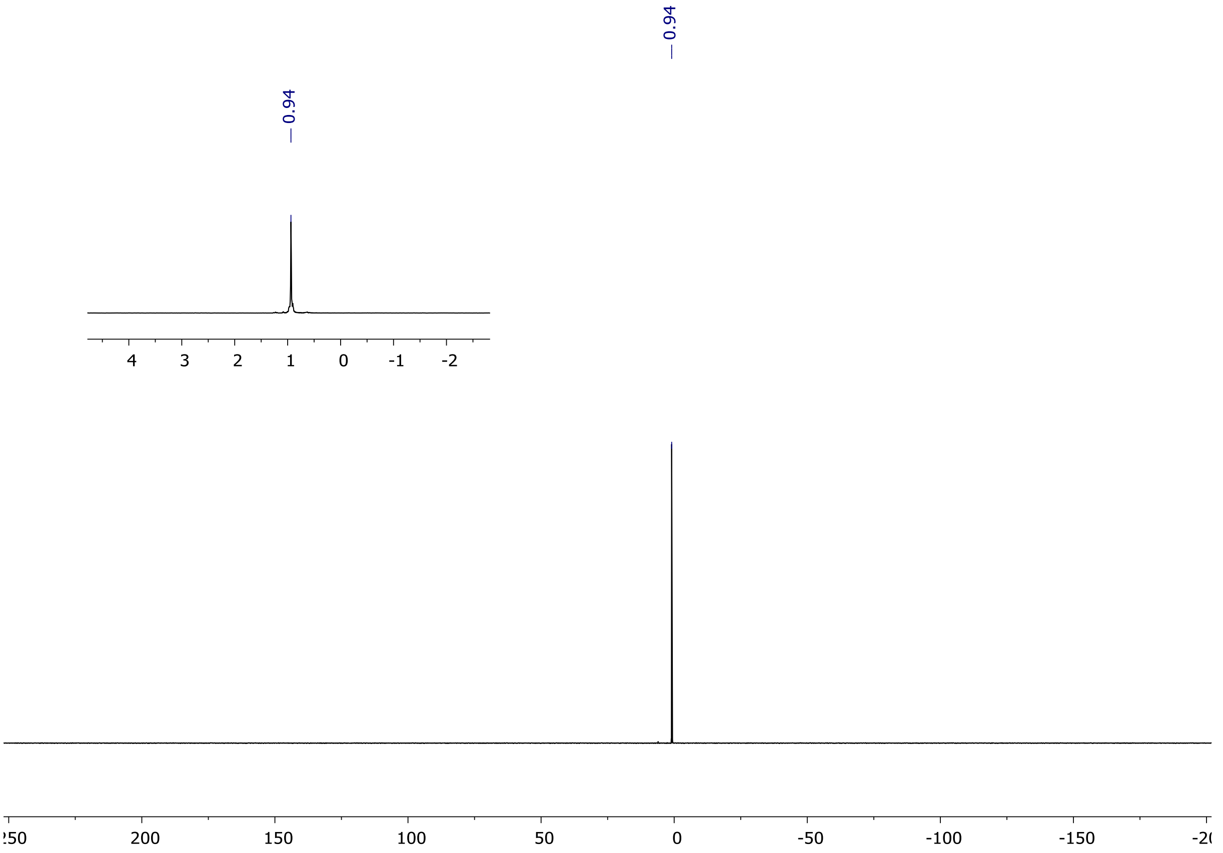

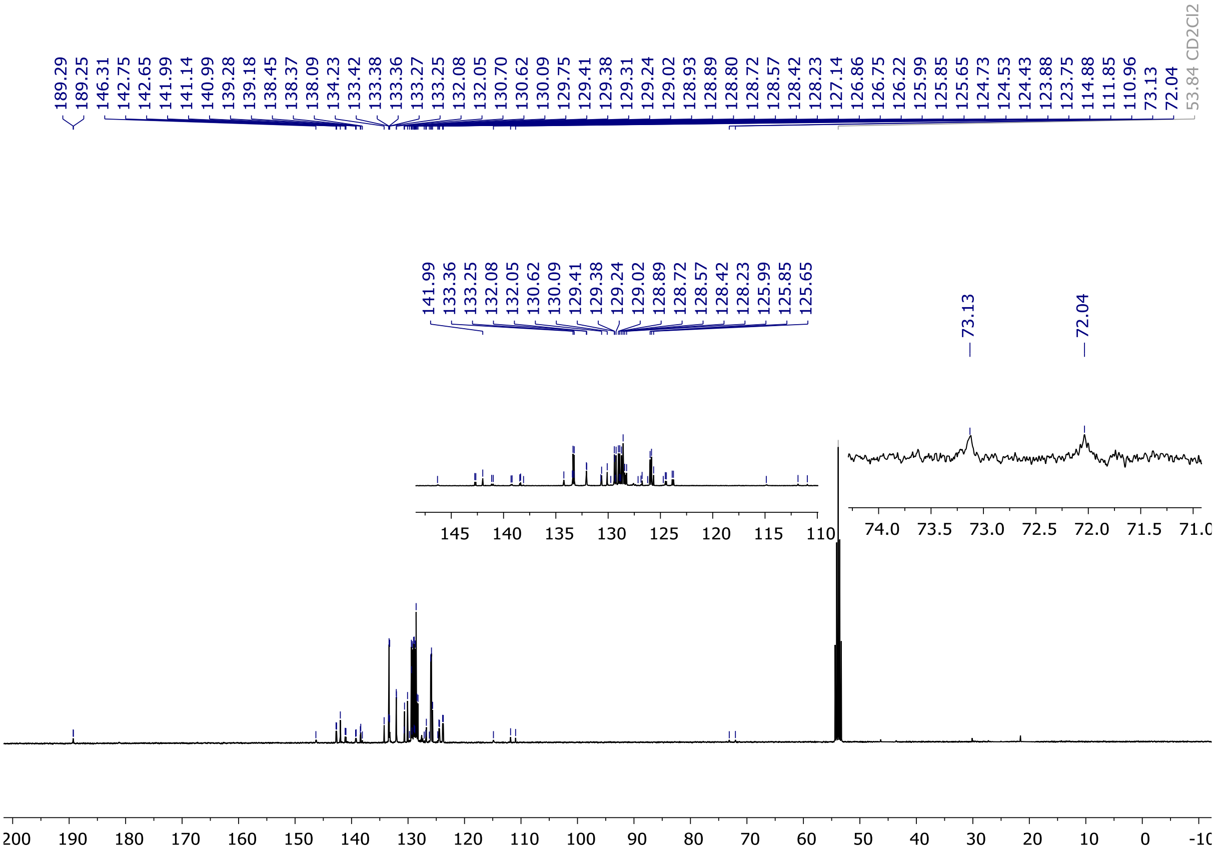

Figure S13: ^{1}H NMR (400 MHz, CD_2Cl_2, 293K), ^{13}C NMR (101 MHz, CD_2Cl_2, 293K) and $^{31}P\{^{1}H\}$ NMR (162 MHz, CD_2Cl_2, 293K) spectra of compound **11.**

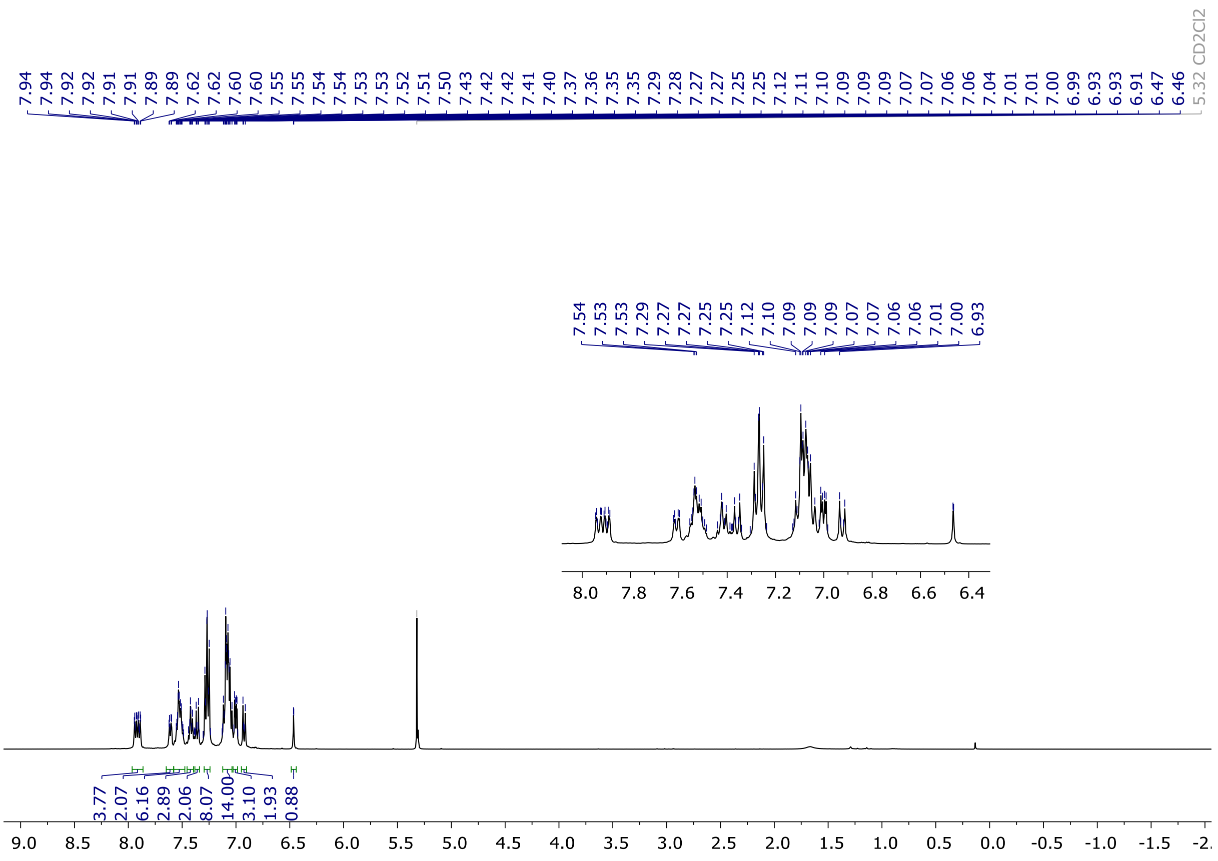

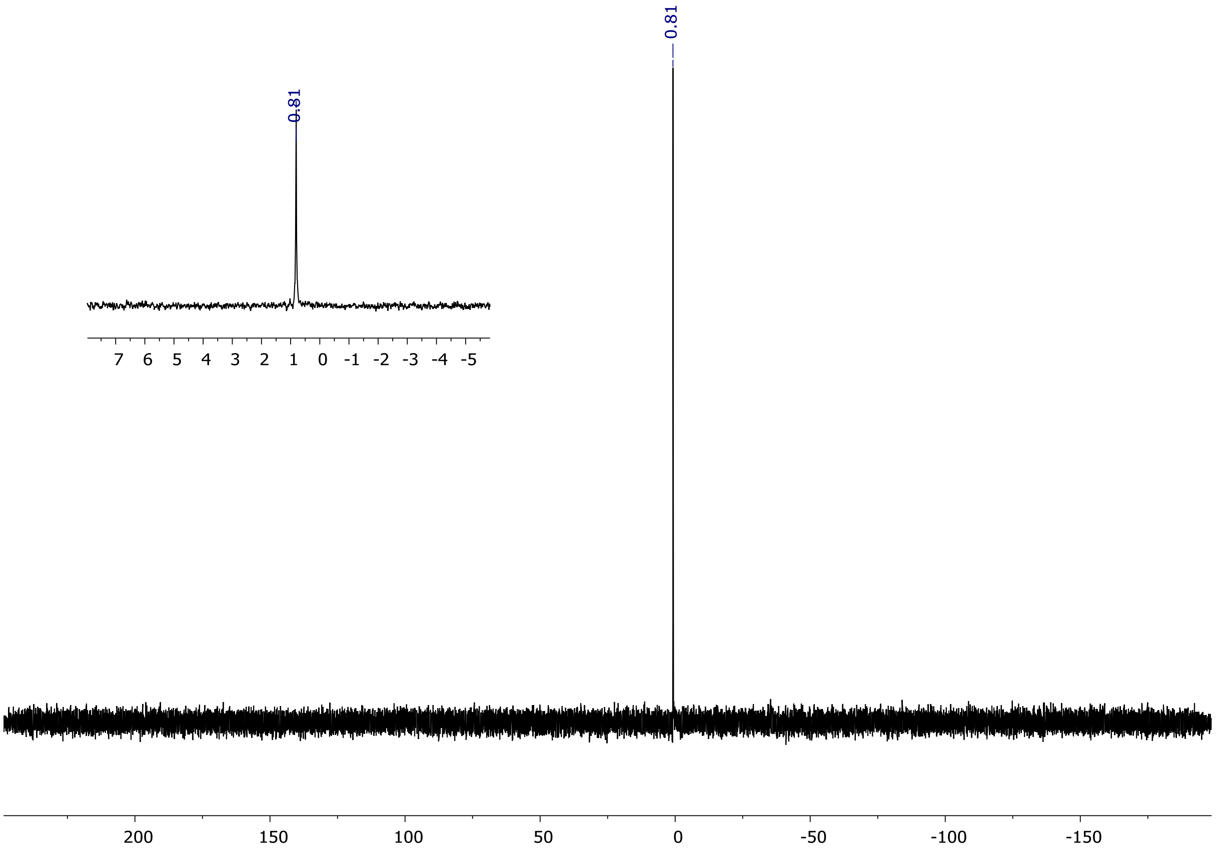

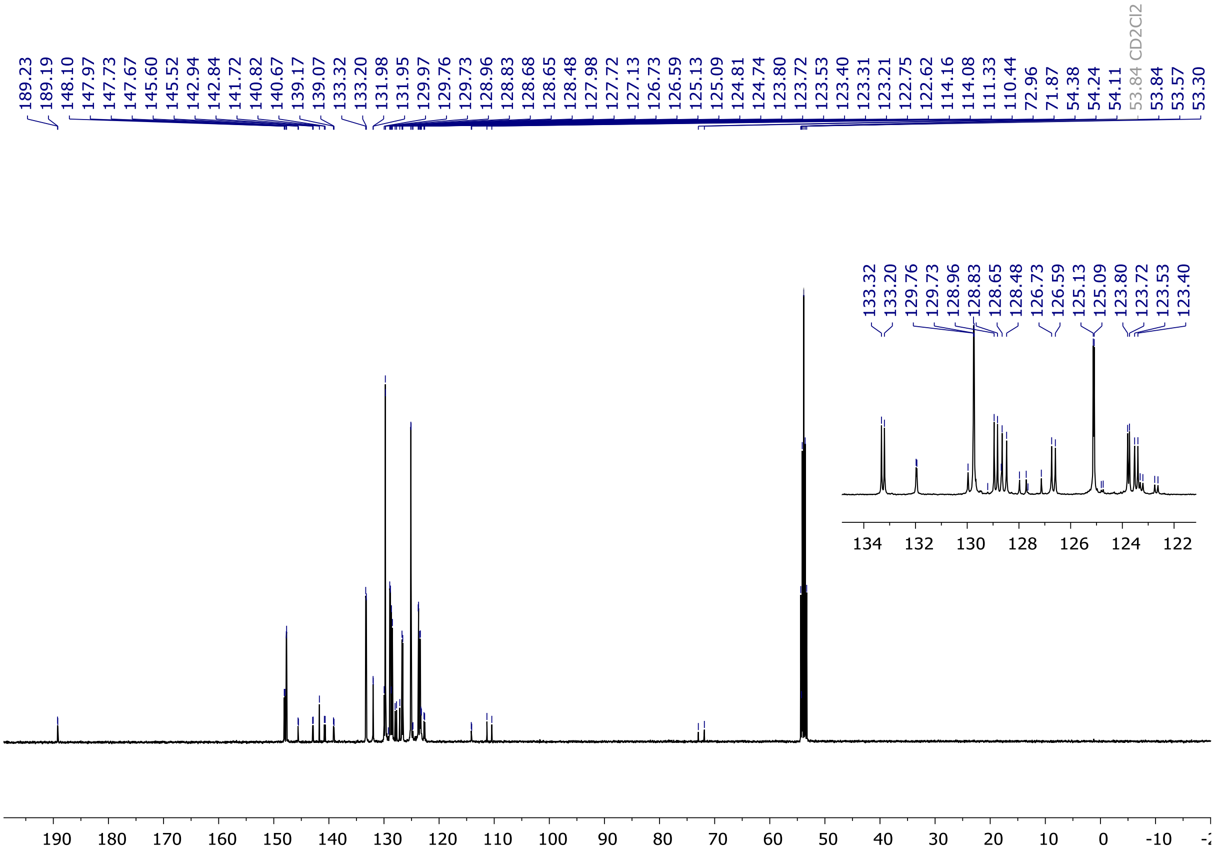

Figure S14: ^{1}H NMR (400 MHz, CD_2Cl_2, 293K), ^{13}C NMR (101 MHz, CD_2Cl_2, 293K) and $^{31}P\{^{1}H\}$ NMR (121 MHz, CD_2Cl_2, 293K) spectra of compound **12.**

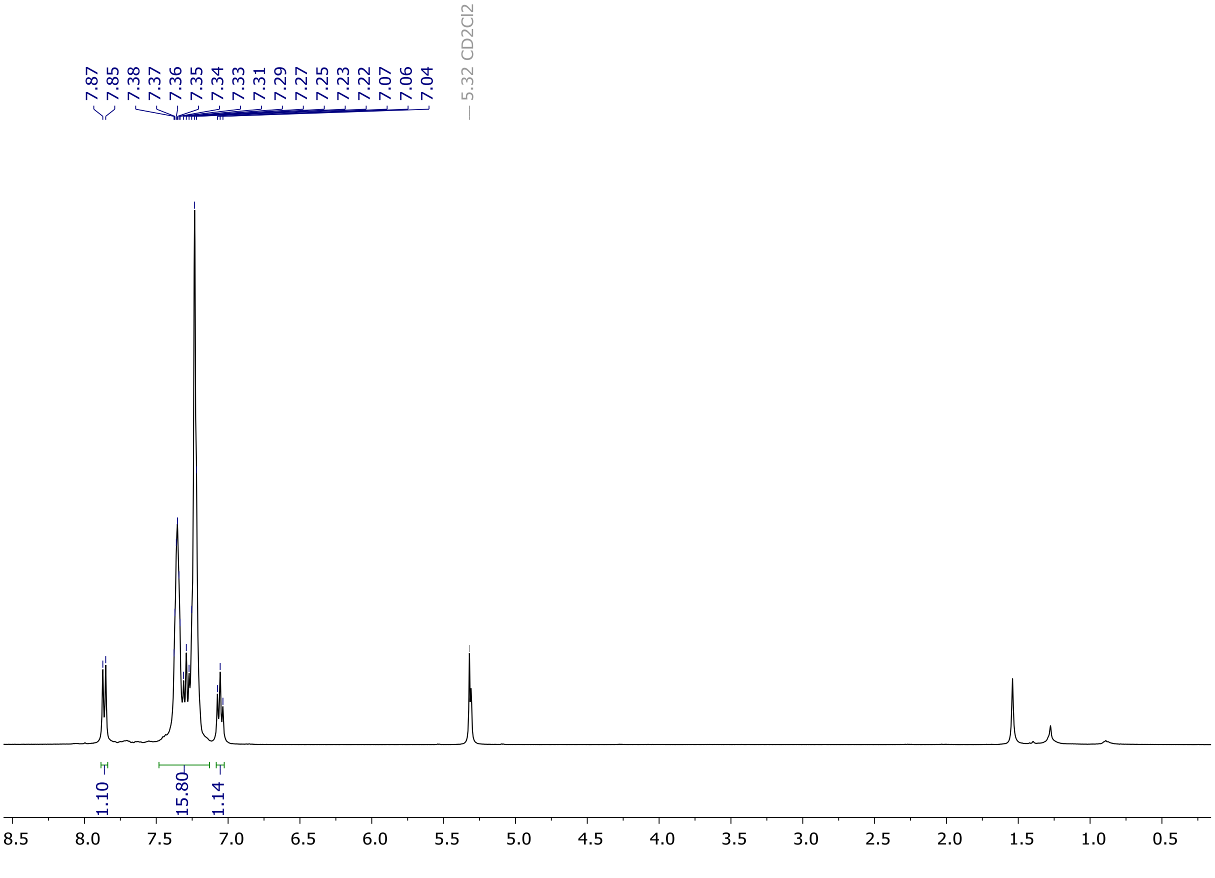

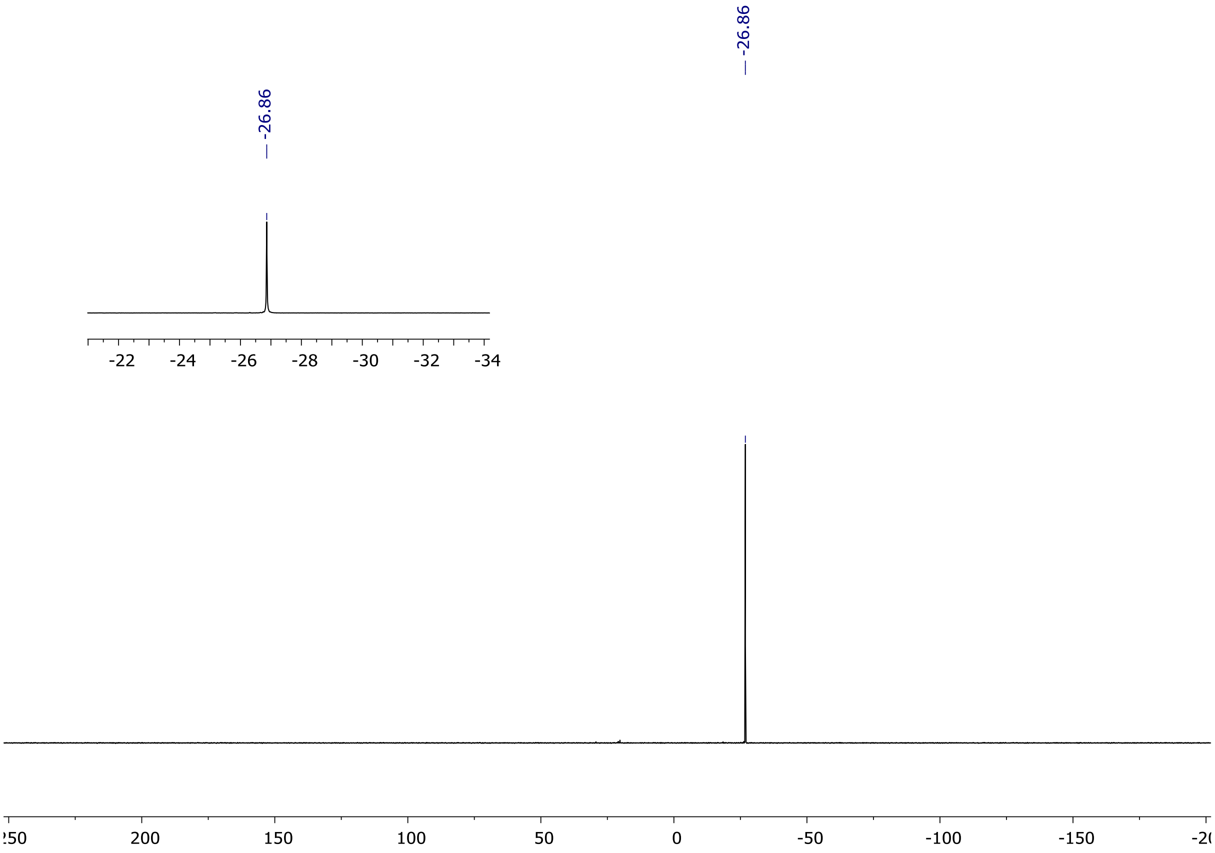

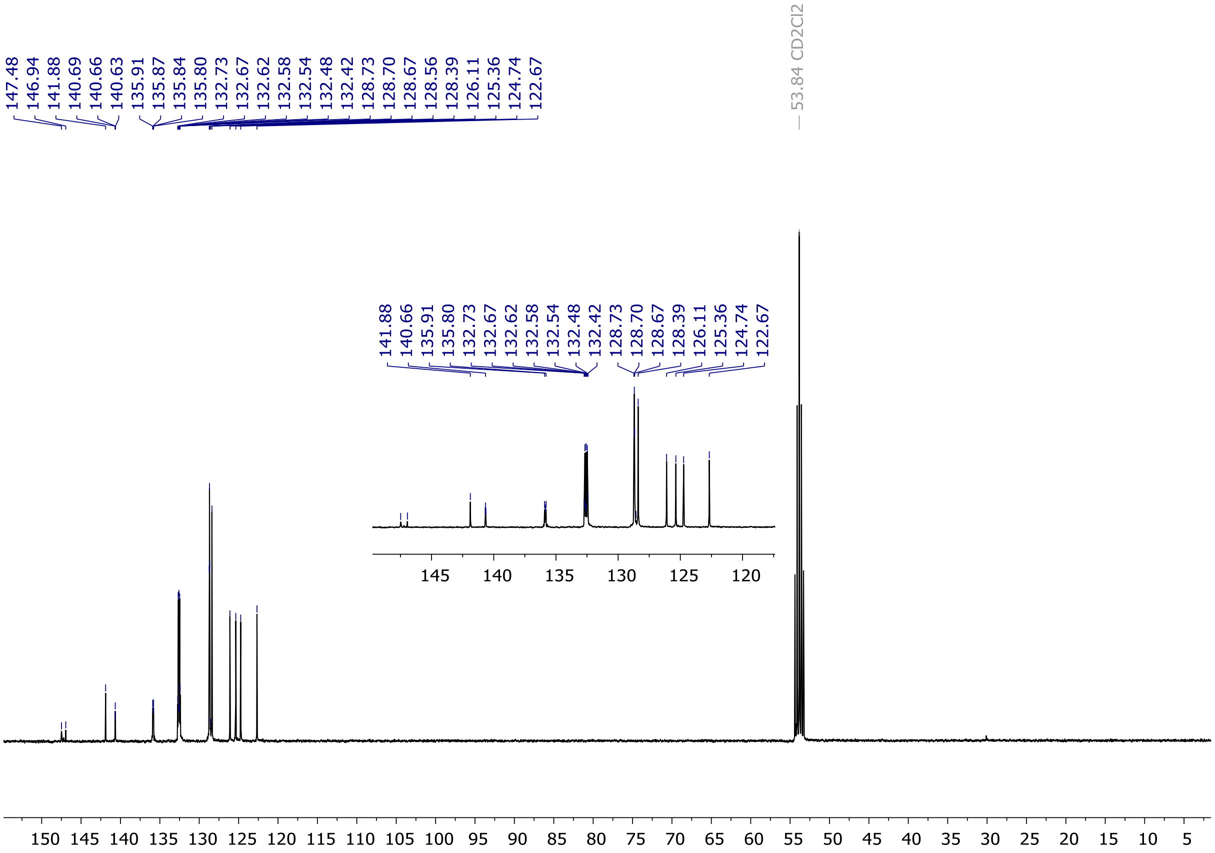

Figure S15: ^{1}H NMR (400 MHz, CD_2Cl_2, 293K), ^{13}C NMR (101 MHz, CD_2Cl_2, 293K) and $^{31}P\{^{1}H\}$ NMR (162 MHz, CD_2Cl_2, 293K) spectra of compound **13.**

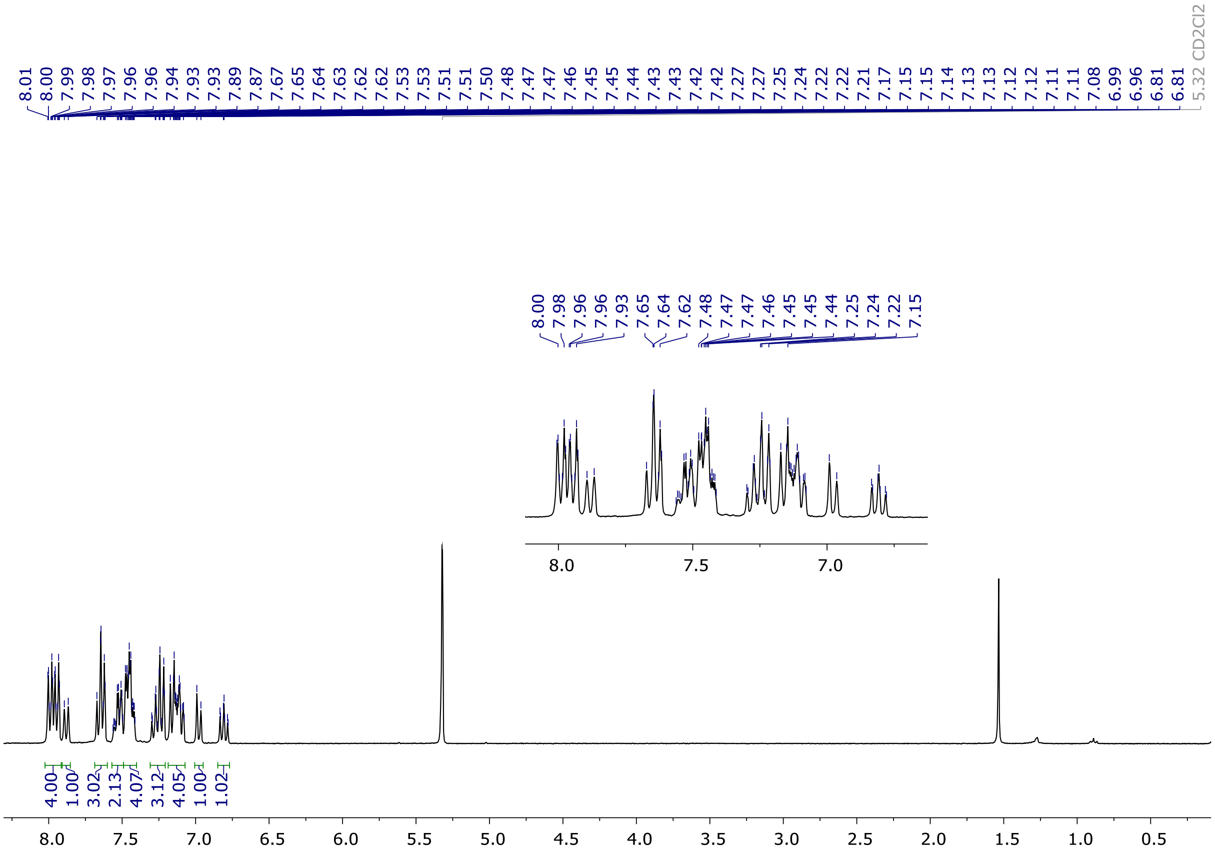

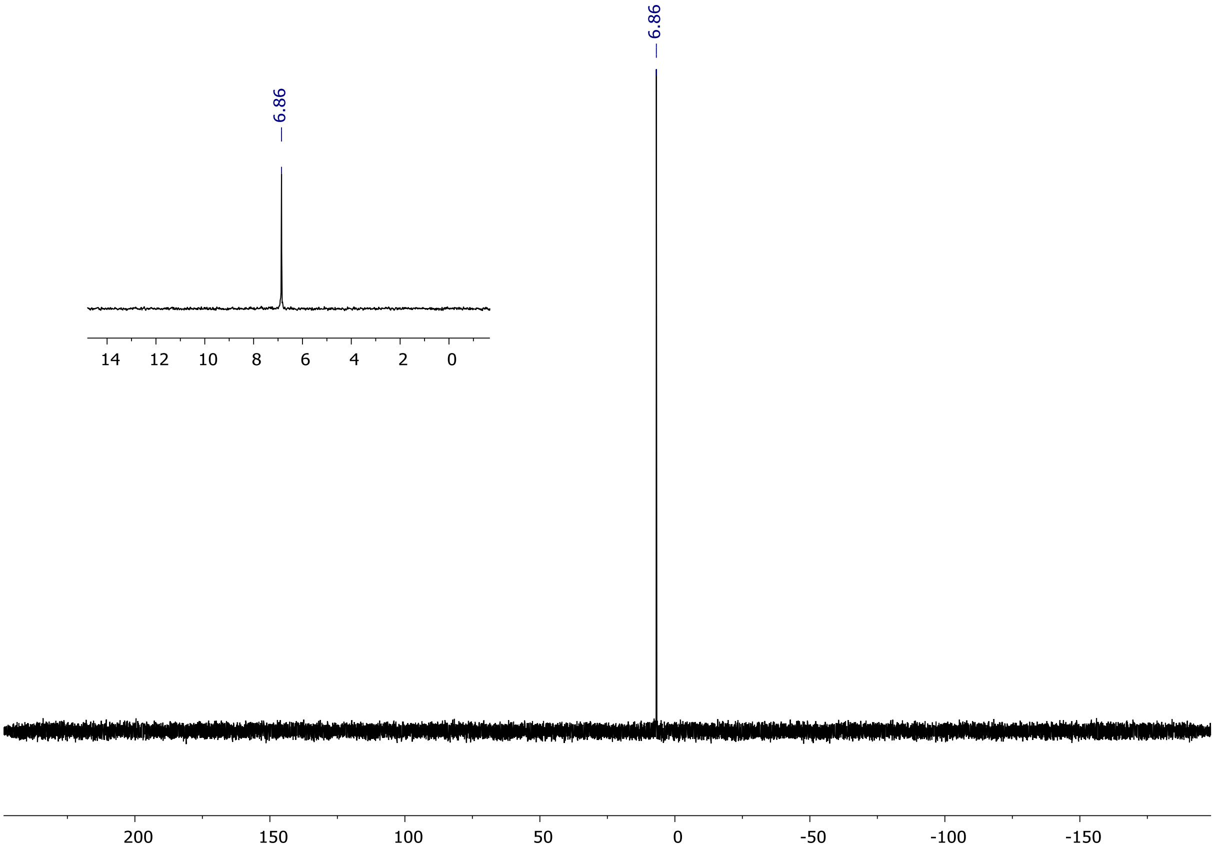

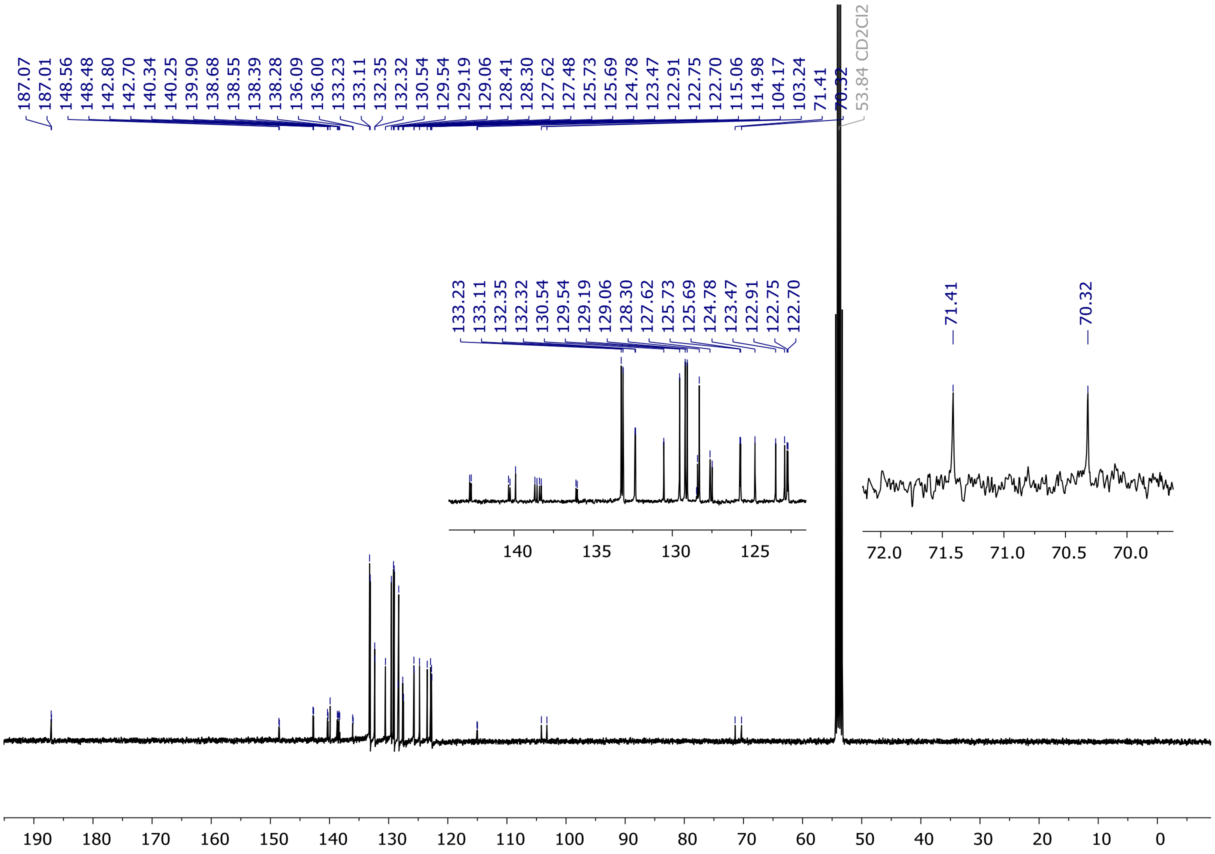

Figure S16: ^{1}H NMR (300 MHz, CD_2Cl_2, 293K), ^{13}C NMR (101 MHz, CD_2Cl_2, 293K) and $^{31}P\{^{1}H\}$ NMR (121 MHz, CD_2Cl_2, 293K) spectra of compound **14.**

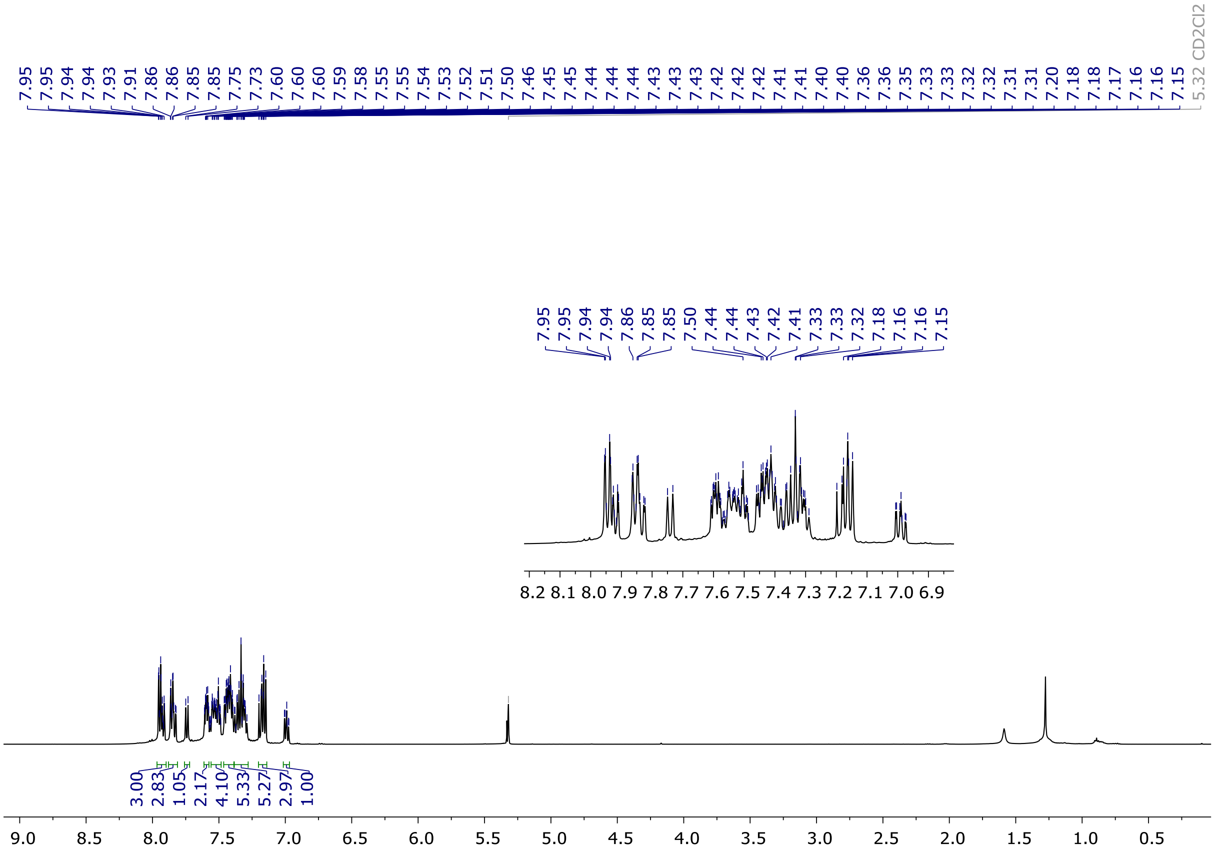

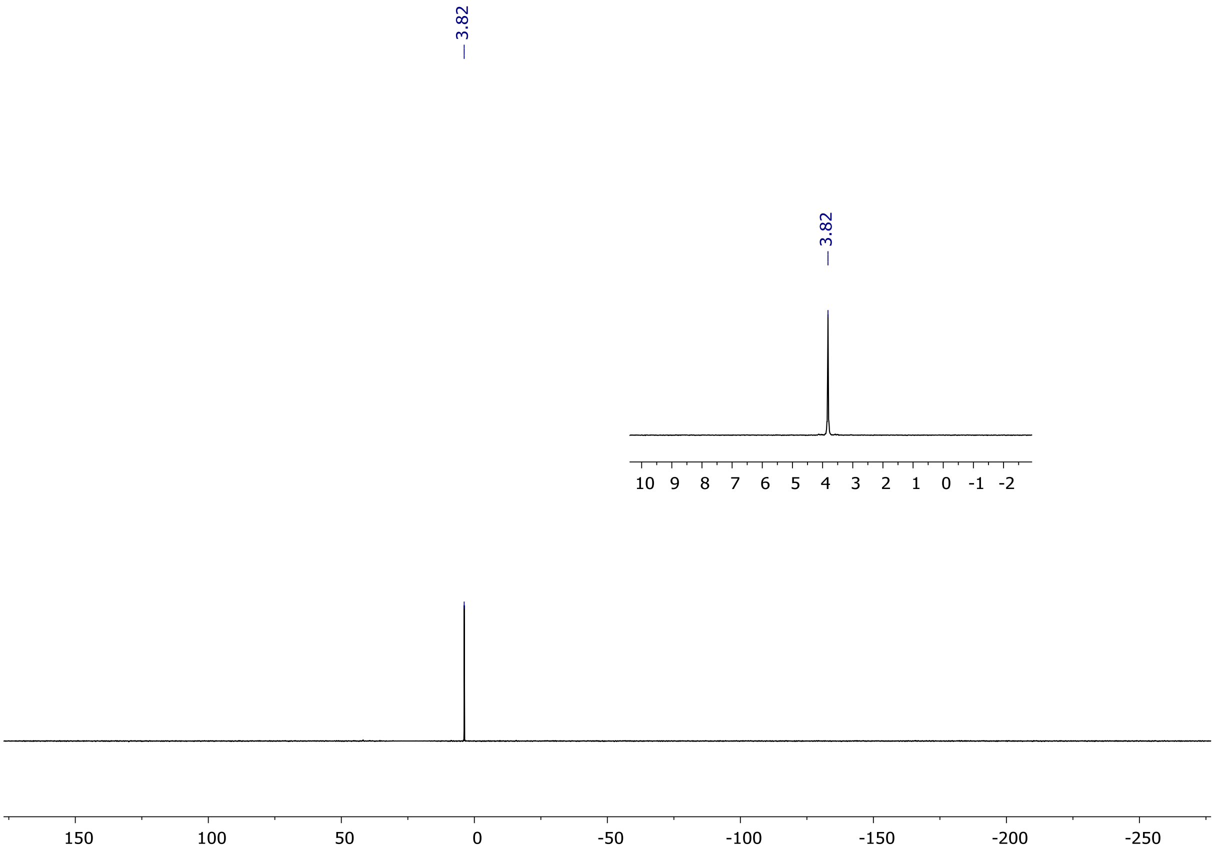

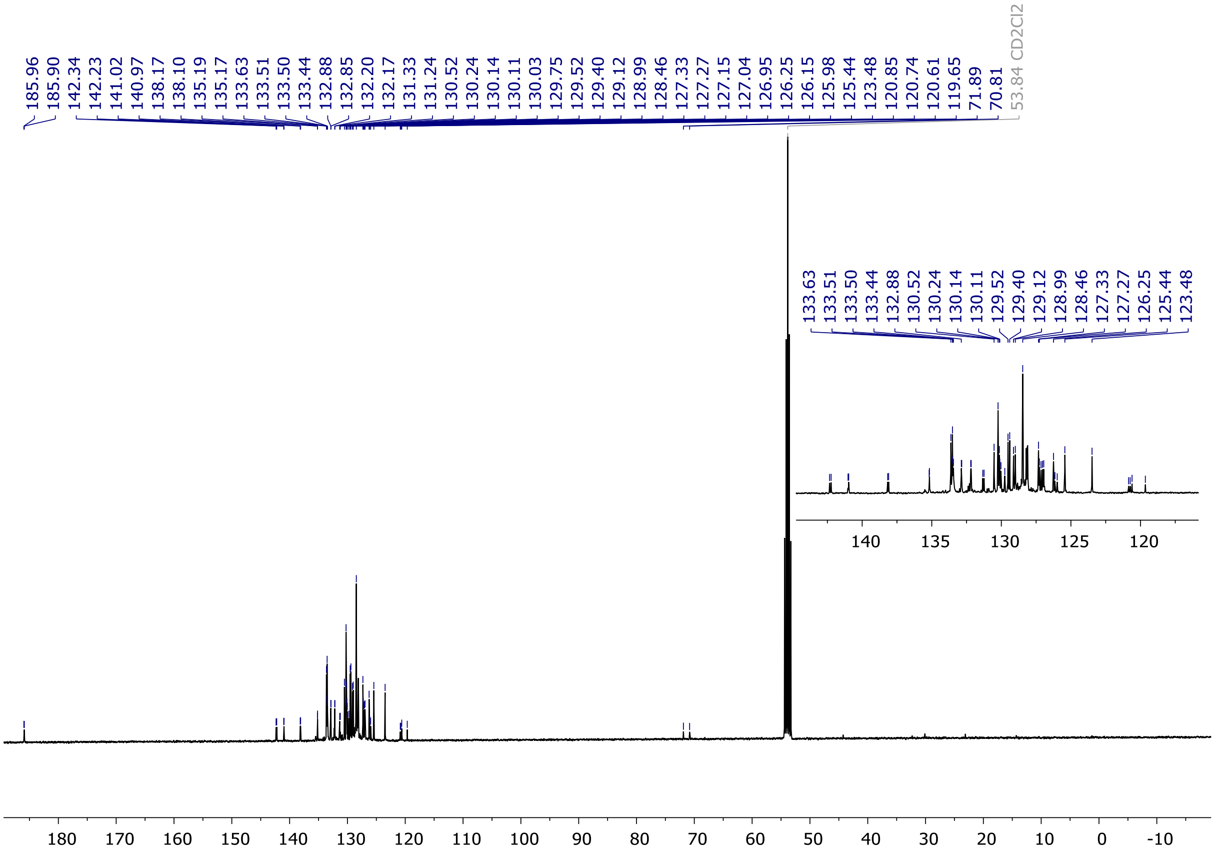

Figure S17: ^{1}H NMR (500 MHz, CD_2Cl_2, 293K), ^{13}C NMR (101 MHz, CD_2Cl_2, 293K) and $^{31}P\{^{1}H\}$ NMR (162 MHz, CD_2Cl_2, 293K) spectra of compound **16a.**

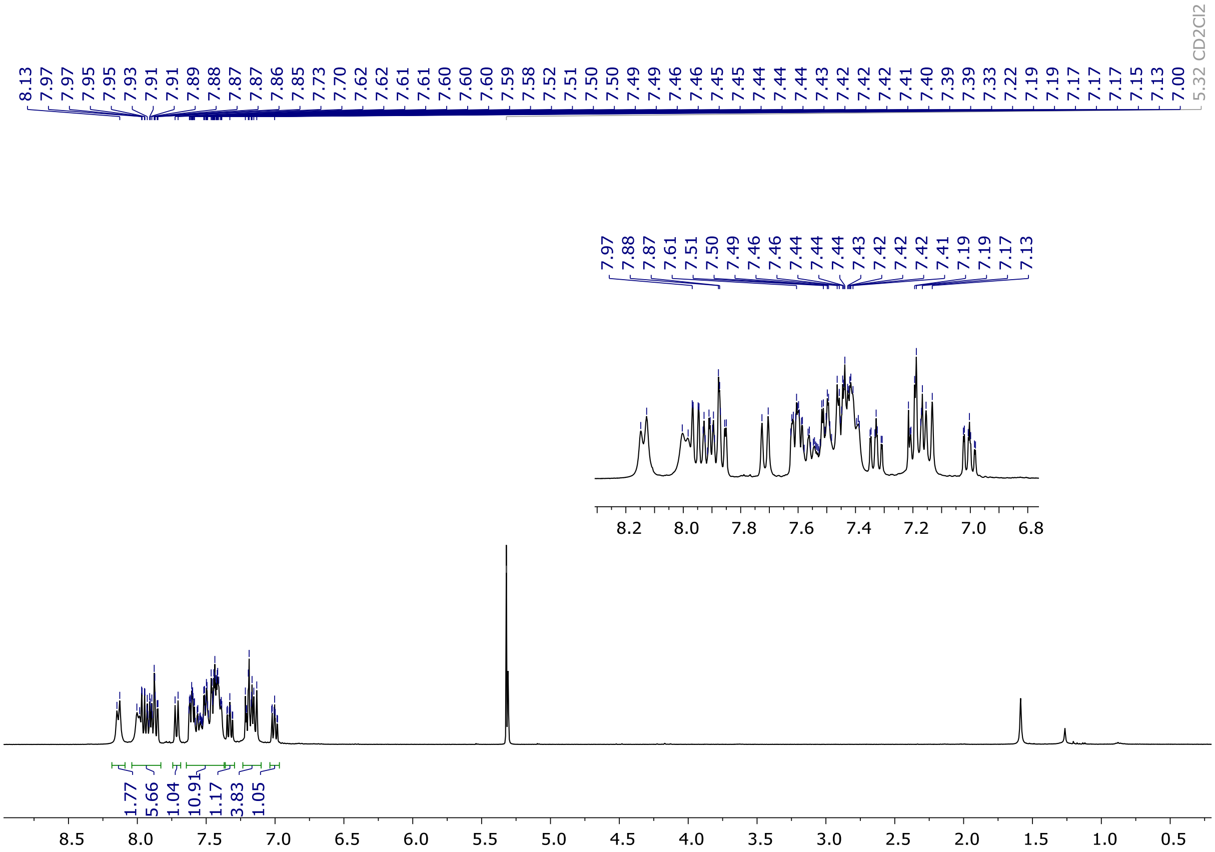

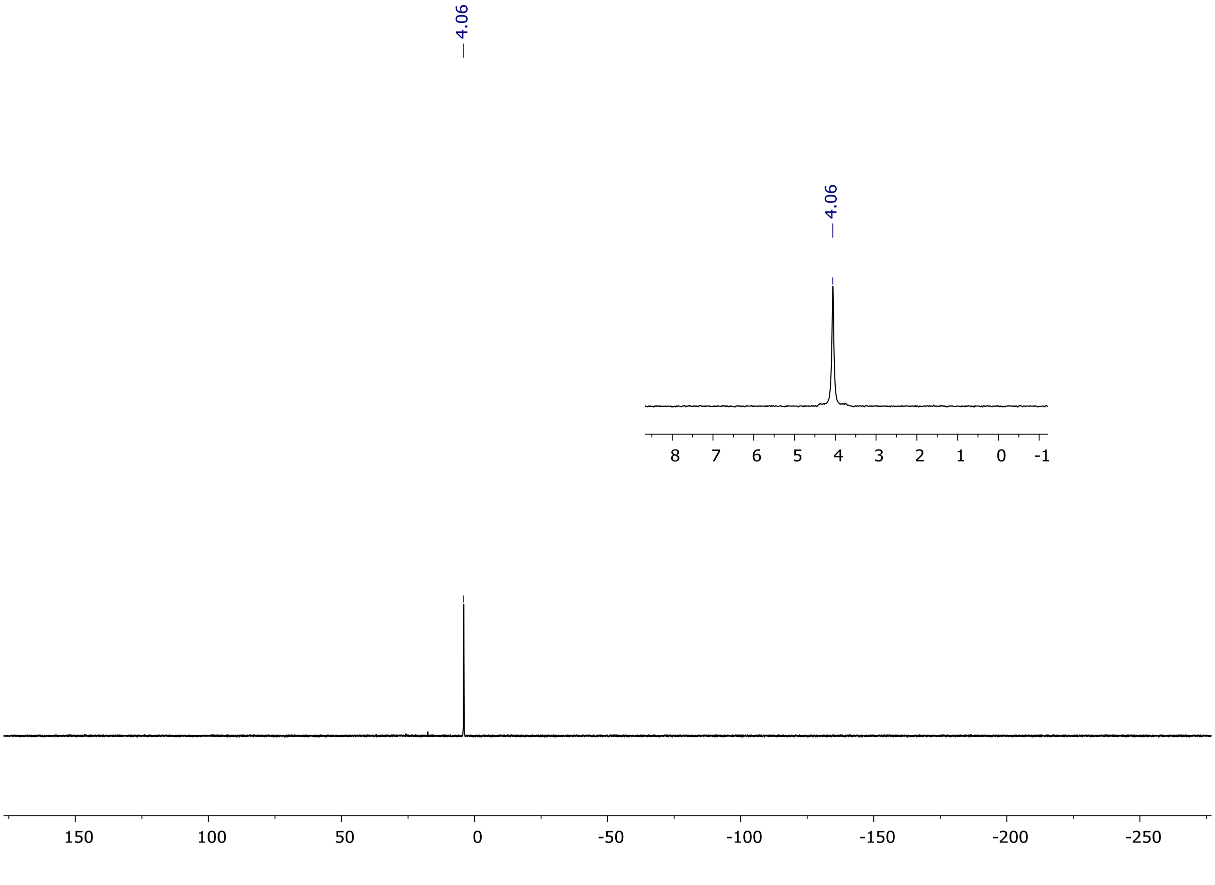

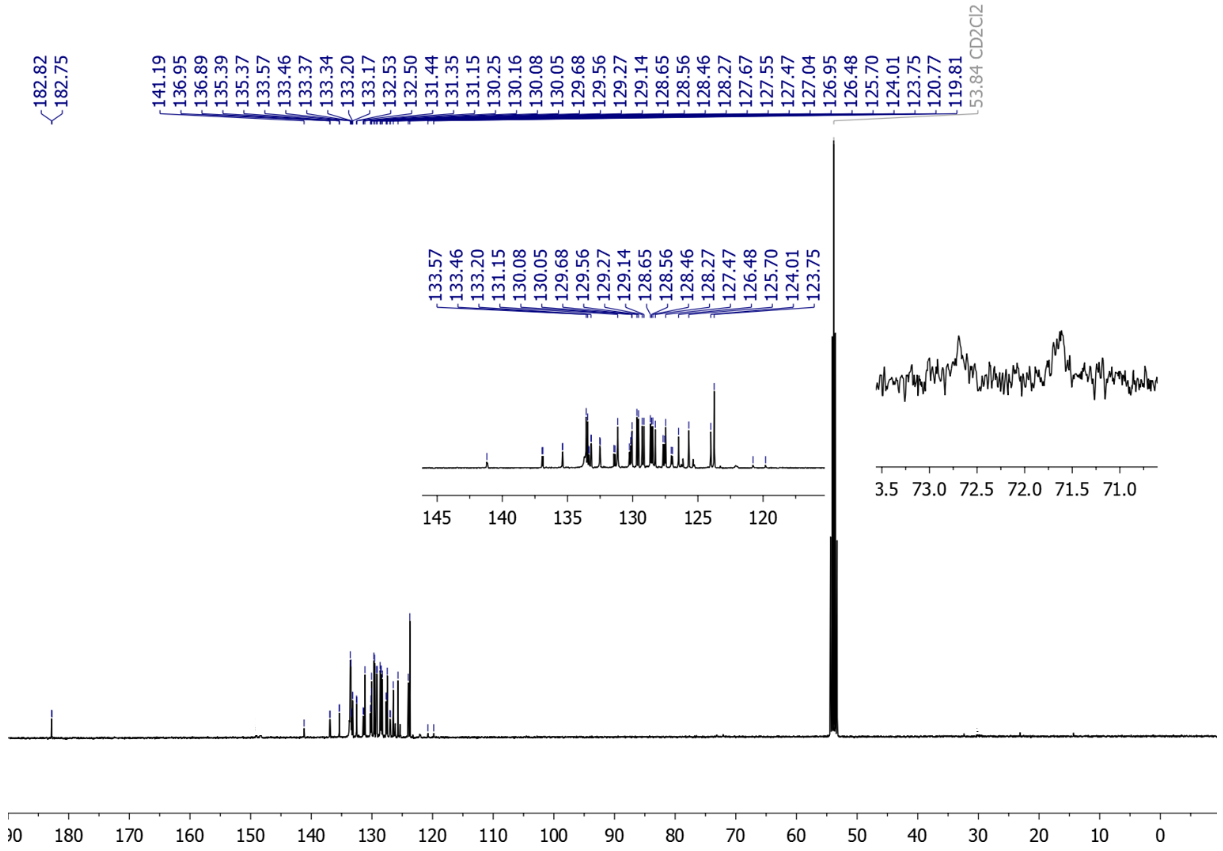

Figure S18: ^{1}H NMR (400 MHz, CD_2Cl_2, 293K), ^{13}C NMR (101 MHz, CD_2Cl_2, 293K) and $^{31}P\{^{1}H\}$ NMR (162 MHz, CD_2Cl_2, 293K) spectra of compound **16b.**

X-ray crystallography

Single crystals suitable for X-Ray crystal analysis were obtained by slow diffusion of vapors of pentane into a dichloromethane solution of the derivatives at rt. Single crystal data collection were performed at 150 K with an D8 Venture Bruker-AXS diffractometer with Mo-$K\alpha$ radiation (λ = 0.71073 Å). The structure was solved by dual-space algorithm using the *SHELXT* program, and then refined with full-matrix least-squares methods based on F^2(*SHELXL*). All non-hydrogen atoms were refined with anisotropic atomic displacement parameters. H atoms were finally included in their calculated positions and treated as riding on their parent atom with constrained thermal parameters.

Table S1: Crystallographic data

**Compound**	**5a**	**5b**	**5f**	**7a**
CCDC	2143639	2143642	2143643	2143644
Formula	C30 H21 O P	C30 H20 N O3 P	C30 H33 O P	C32 H23 O P
MW	428.44	473.44	440.53	454.47
a (Å)	*12.0964(11)*	*10.8566(16)*	*12.5560(11)*	*10.1144(8)*
b (Å)	*11.0843(13)*	*15.166(2)*	*13.9341(10)*	*14.5902(12)*
c (Å)	*19.243(2)*	*17.859(3)*	*13.9603(12)*	*17.2815(12)*
α (°)	90	79.317(5)	82.137(3)	90.342
β (°)	104.733(4)	75.424(5)	86.710(3)	97.270(3)
γ (°)	90	70.381(5)	89.314(3)	103.927(3)
V (Å^3)	*2495.3(5)*	*2664.2(7)*	*2415.5(3)*	*2453.6(3)*
Z	*4*	*4*	*4*	*4*
*D*c (g.cm^{-3})	*1.140*	*1.180*	*1.211*	*1.230*
Crystal system	monoclinic	triclinic	triclinic	triclinic
Space group	P 21/n	P -1	P -1	P -1
T (K)	150	150	150	150
WavelengthMo-Kα (Å)	0.71073	0.71073	0.71073	0.71073
μ (mm^{-1})	0.128	0.133	0.134	0.134
F (000)	*896*	*984*	*944*	*952*
θ limit (°)	2.139 to 27.523	2.371 to 27.666	2.132 to 27.521	2.267 to 27.566
Index ranges *hkl*	-15 ≤ h ≥ 15	-13 ≤ h ≥ 14	-16 ≤ h ≥ 16	-13 ≤ h ≥ 13
	-14 ≤ k ≥ 14	-19 ≤ k ≥ 19	-18 ≤ k ≥ 15	-18 ≤ k ≥ 18
	-24 ≤ l ≥ 25	0 ≤ l ≥ 23	-18 ≤ l ≥ 18	-21 ≤ l ≥ 22
Reflectionscollected	28373	11965	45844	42583
Independantreflections	5671	11965	11020	11188
Reflections [*I*>2σ(*I*)]	4139	8631	8858	8699
Data / restraints / parameters	5671 / 0 / 290	11965 / 0 / 632	11020 / 0 / 577	11188 / 0 / 613
Goodness-of-fit on *F*^{2}	1.025	1.085	0.993	1.030
Final *R* indices [I>2σ(I)]	R1 = 0.0636	R1 = 0.0927	R1 = 0.0431	R1 = 0.0521
	wR2 = 0.1646	*wR2* = 0.2478	*wR2 = 0.1064*	*wR2* = 0.1182
R indices (all data)	*R*1= 0.0902	*R*1= 0.1283	*R*1= 0.0588	*R*1= 0.0723
	*wR*2= 0.1860	*wR*2= 0.2738	*wR*2= 0.1178	*wR*2= 0.1288
Larg. Diff. peak / hole (e Å^{-3})	0.590 and -0.544	0.722 and -0.676	0.317 and -0.336	0.363 and -0.387

**Compound**	**9**	**10’**	**10**	**14**
CCDC	2143645	2143649	2143648	2143650
Formula	C28H19OPS2	C71H50Cl6N2O6P2S4	C41H27Cl2N2O5 PS2	C36H23OPS2
MW	466.52	1430.01	793.63	566.63
a (Å)	*9.9301(8)*	*9.3695(11)*	*16.921(2)*	*8.3232(7)*
b (Å)	*12.6533(13)*	*11.2239(15)*	*13.9828(13)*	*14.8898(14)*
c (Å)	*18.2991(16)*	*15.7853(19)*	*15.5120(18)*	*22.077(2)*
α (°)	90	92.323(5)	90	90
β (°)	100.319(3)	91.708(5)	97.303(4)	100.327
γ (°)	90	104.146(4)	90	90
V ($Å^3$)	*2262.1(4)*	*1607.0(3)*	*3640.4(7)*	*2691.7(4)*
Z	*4*	*1*	*4*	*4*
Dc ($g.cm^{-3}$)	*1.370*	*1.478*	*1.448*	*1.398*
Crystal system	monoclinic	triclinic	monoclinic	monoclinic
Space group	P 21/c	P -1	P 21/c	P 21/c
T (K)	150	150	150	150
WavelengthMo-Kα (Å)	0.71073	0.71073	0.71073	0.71073
μ (mm^{-1})	0.325	0.504	0.387	0.288
F (000)	*968*	*734*	*1632*	*1176*
θ limit (°)	2.634 to 27.502	2.223 to 27.497	2.222 to 25.680	2.321 to 27.503
Index ranges *hkl*	-12 ≤ h ≥ 12<br>-14 ≤ k ≥ 16<br>-23 ≤ l ≥ 23	-12 ≤ h ≥ 11<br>-14 ≤ k ≥ 14<br>-20 ≤ l ≥ 20	-20 ≤ h ≥ 20<br>-17 ≤ k ≥ 16<br>-18 ≤ l ≥ 18	-7 ≤ h ≥ 10<br>-19 ≤ k ≥ 19<br>-28 ≤ l ≥ 28
Reflectionscollected	23959	22210	40352	22710
Independantreflections	5168	7299	6902	6159
Reflections [*I*>2σ(*I*)]	4307	5660	5614	4901
Data / restraints / parameters	5168 / 0 / 289	7299 / 0 / 424	6902 / 55 / 535	6159 / 0 / 361

Goodness-of-fit on F^2	1.053	1.018	1.192	1.026
Final *R* indices [I>2σ(I)]	R1 = 0.0494	R1 = 0.0915	R1 = 0.1184	R1 = 0.0491
	wR2 = 0.1257	*wR2 = 0.2353*	*wR2 = 0.3043*	*wR2 = 0.1222*
R indices (all data)	*R*1= 0.0606,	*R1 = 0.1135*	*R1 = 0.1388*	*R1 = 0.0672*
	*wR*2= 0.1351	*wR2 = 0.2576*	*wR2 = 0.3263*	*wR2 = 0.1338*
Larg. diff peak / hole (e $Å^{-3}$)	0.945 and -0.562	1.361 and -1.126	1.610 and -1.193	0.460 and - 0.538

Table S2. Distances and angles

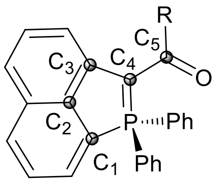

	5a	5b	5f
P-C_1 (Å)	1,794(2)	1,795(6)/1,791(5)	1,801(2)/1,799(2)
P-C_4 (Å)	1,751(2)	1,745(4)/1,763(6)	1,763(2)/1,756(2)
C_1-C_2(Å)	1,416(3)	1,412(6)/1,414(9)	1,417(2)/1,416(2)
C_2-C_3 (Å)	1,441(3)	1,430(8)/1,438(6)	1,438(2)/1,435(2)
C_3-C_4 (Å)	1,462(3)	1,464(6)/1,472(8)	1,460(2)/1,463(2)
C_4-C_5(Å)	1,416(3)	1,403(5)/1,396(7)	1,404(2)/1,400(2)
C_5=O (A)	1,257(3)	1,254(5)/1,263(7)	1,260(2)/1,264(2)
C_1-P-C_4(°)	94,8(1)	93,8(2)/94,6(2)	94,17(7)/94,30(7)
P- - -O (A)	2,905	2,945/2,901	2,907/2,882

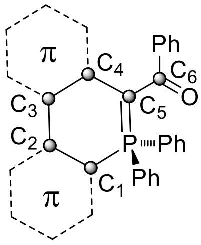

	**7a**[2]	**9**
P-C_1 (Å)	1,807(2)/1,795(2)	1.7773(19)
P-C_5 (Å)	1,729(2)/1,728(2)	1.758(2)
C_1-C_2(Å)	1,412(6)/1,410(2)	1.388(3)
C_2-C_3 (Å)	1,480(3)/1,483(3)	1.425(3)
C_3-C_4 (Å)	1,422(3)/1,425(3)	1.396(3)
C_4-C_5(Å)	1,457(3)/1,459(3)	1.461(3)
C_5-C_6(Å)	1,432(3)/1,438(3)	1.426(3)
C_6=O (A)	1,245(3)/1,249(2)	1.257(2)
C_1-P-C_5(°)	103,2(1)/103,9	105.01(9)
P- - -O (A)	2,906/2,869	2.741

[2] Two independent molecules of **7a** are present in the unit cell

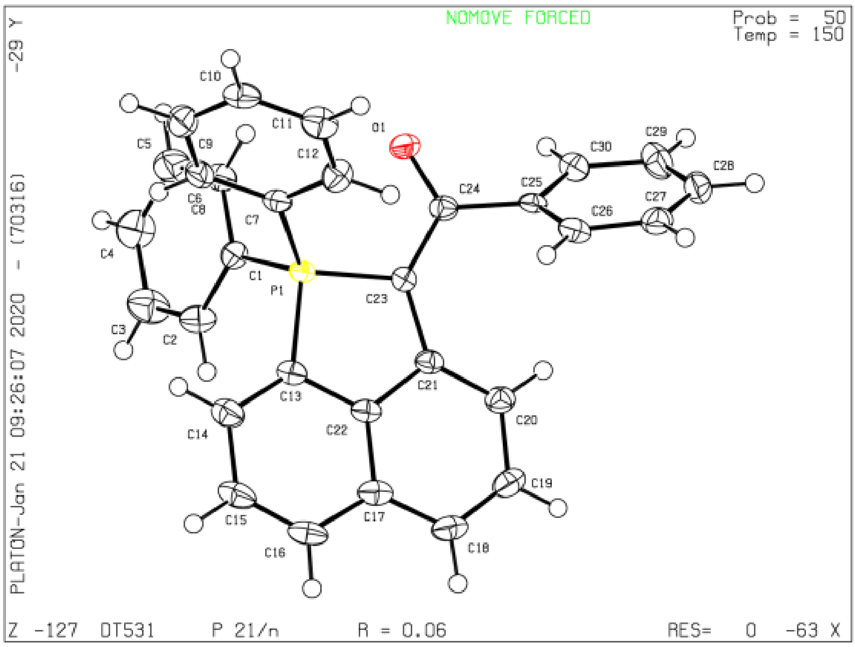

Figure S19: ORTEP representation of **5a** with 50% probability ellipsoids

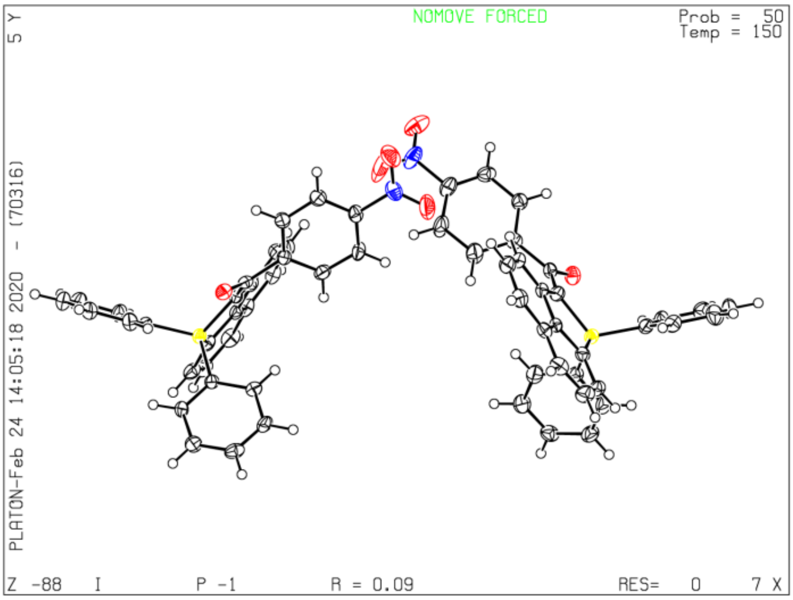

Figure S20: ORTEP representation of **5b** with 50% probability ellipsoids

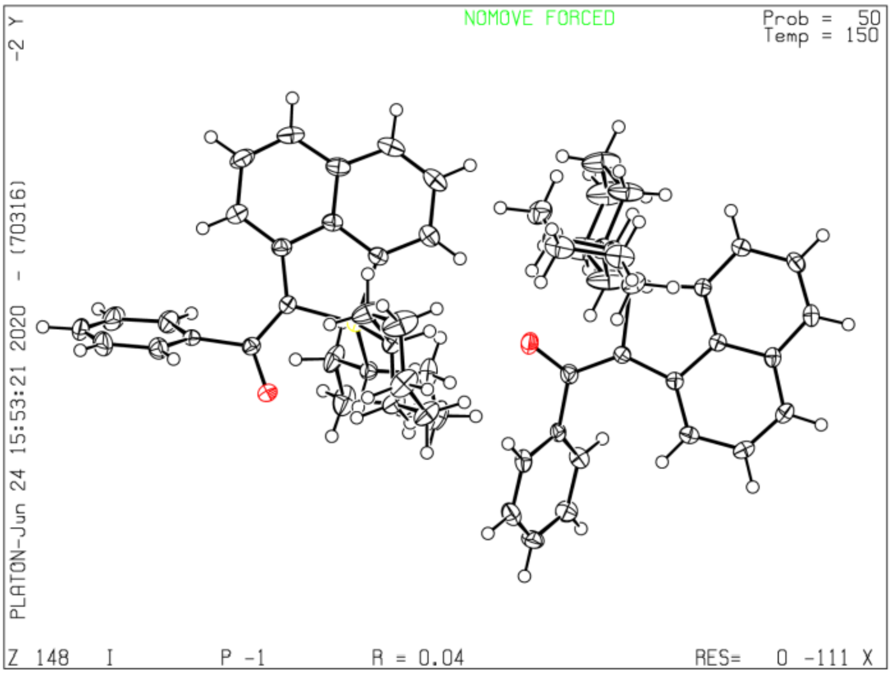

Figure S21: ORTEP representation of **5f** with 50% probability ellipsoids

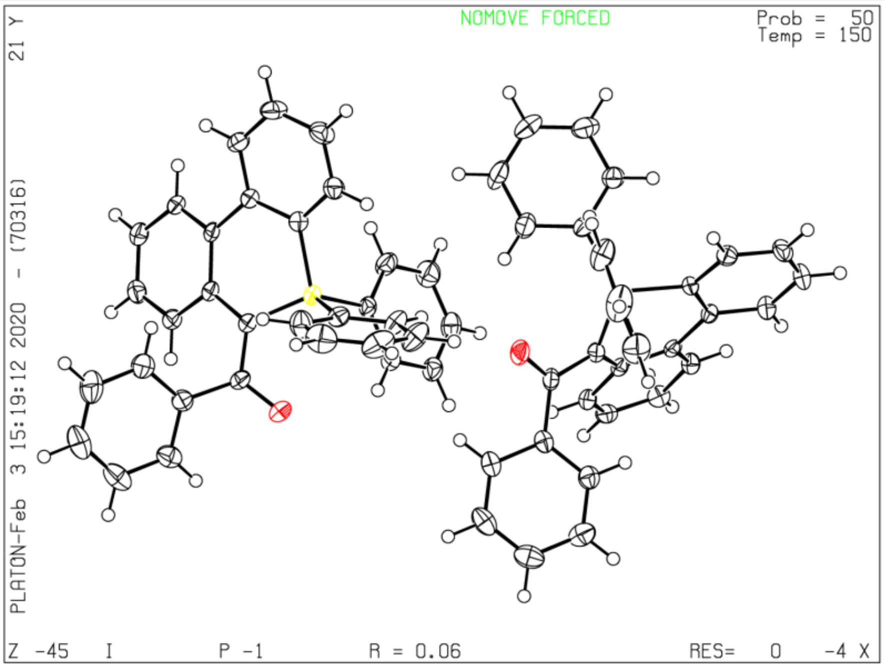

Figure S22: ORTEP representation of **7a** with 50% probability ellipsoids

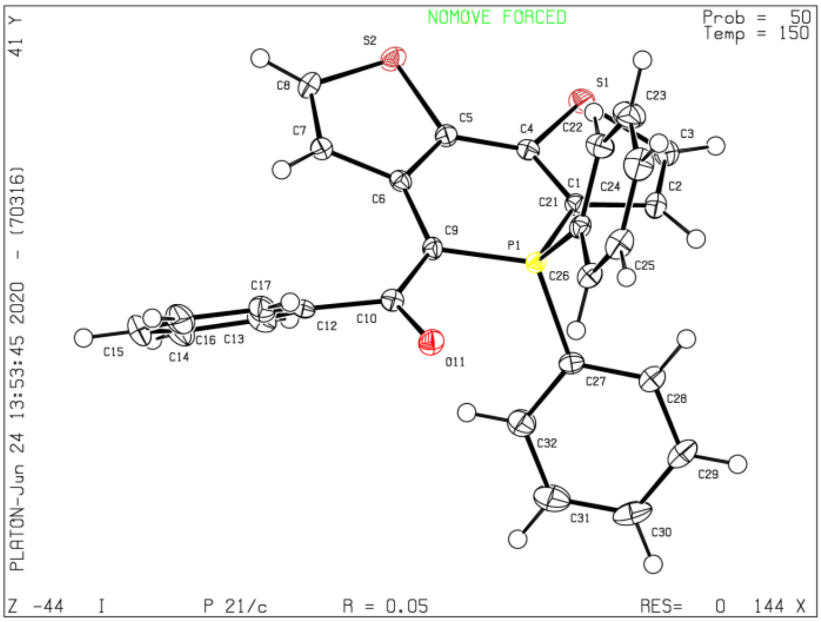

Figure S23: ORTEP representation of **9** with 50% probability ellipsoids

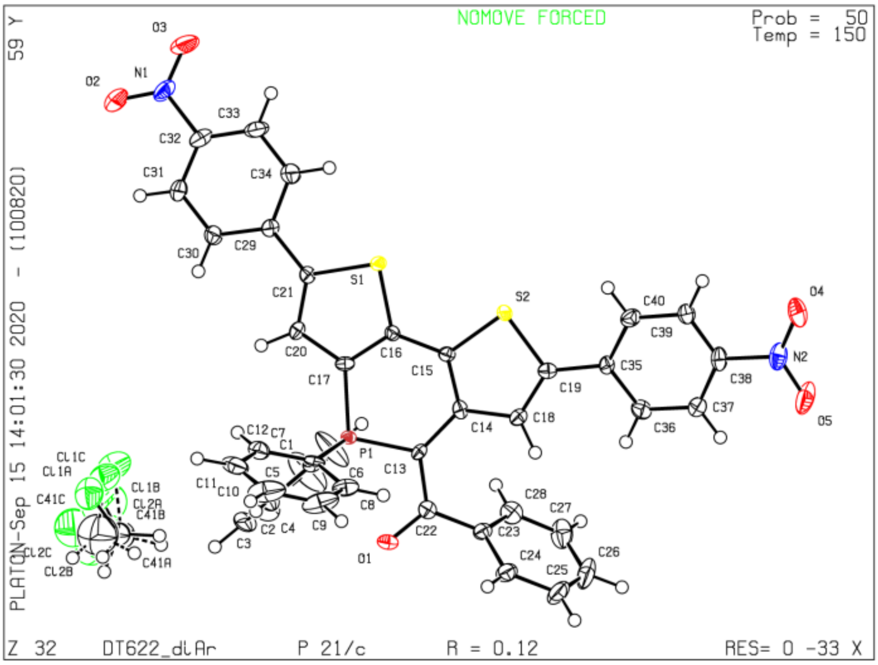

Figure S24: ORTEP representation of **10** with 50% probability ellipsoids

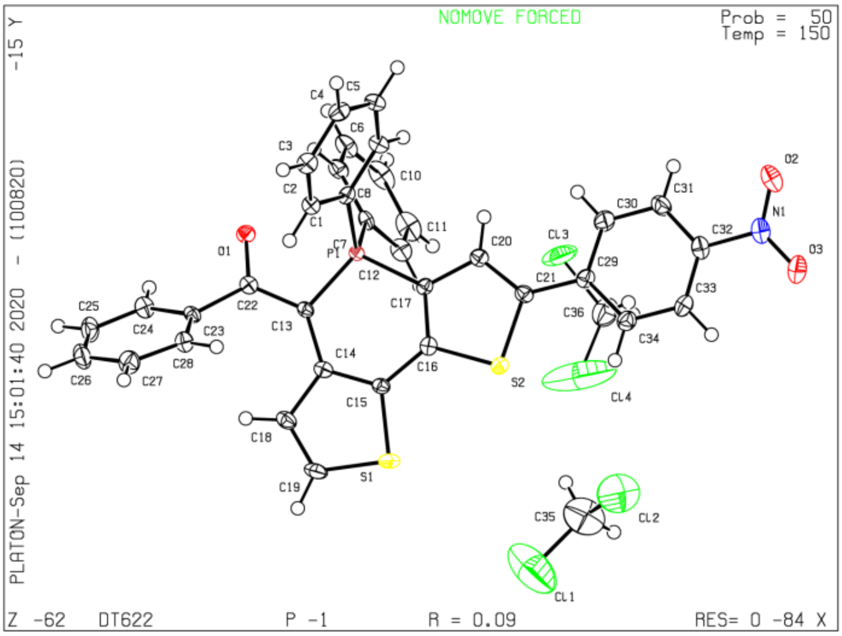

Figure S25: ORTEP representation of **10’** with 50% probability ellipsoids

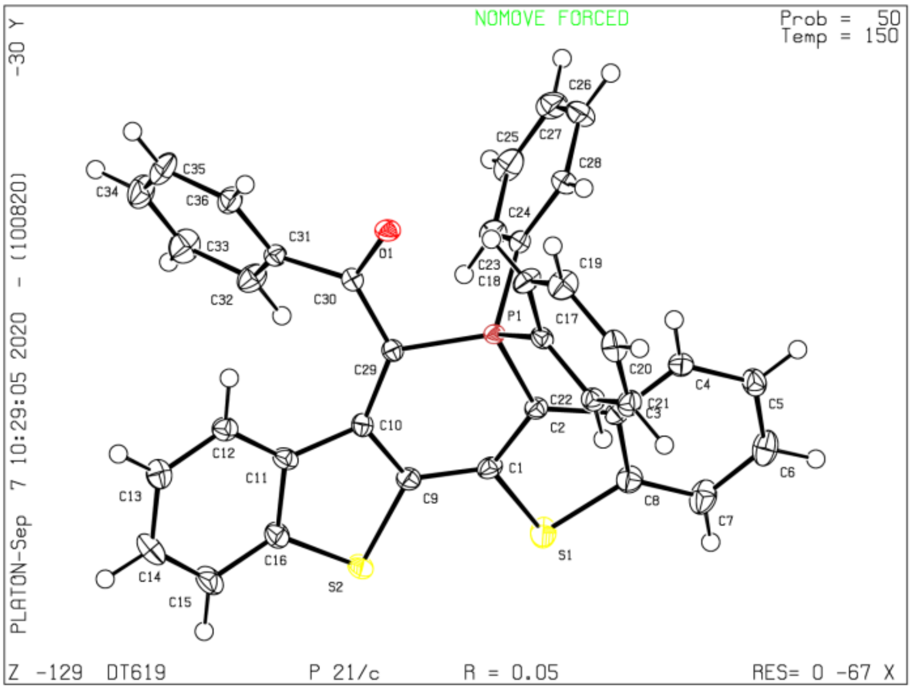

Figure S26: ORTEP representation of **14** with 50% probability ellipsoids

Optical data

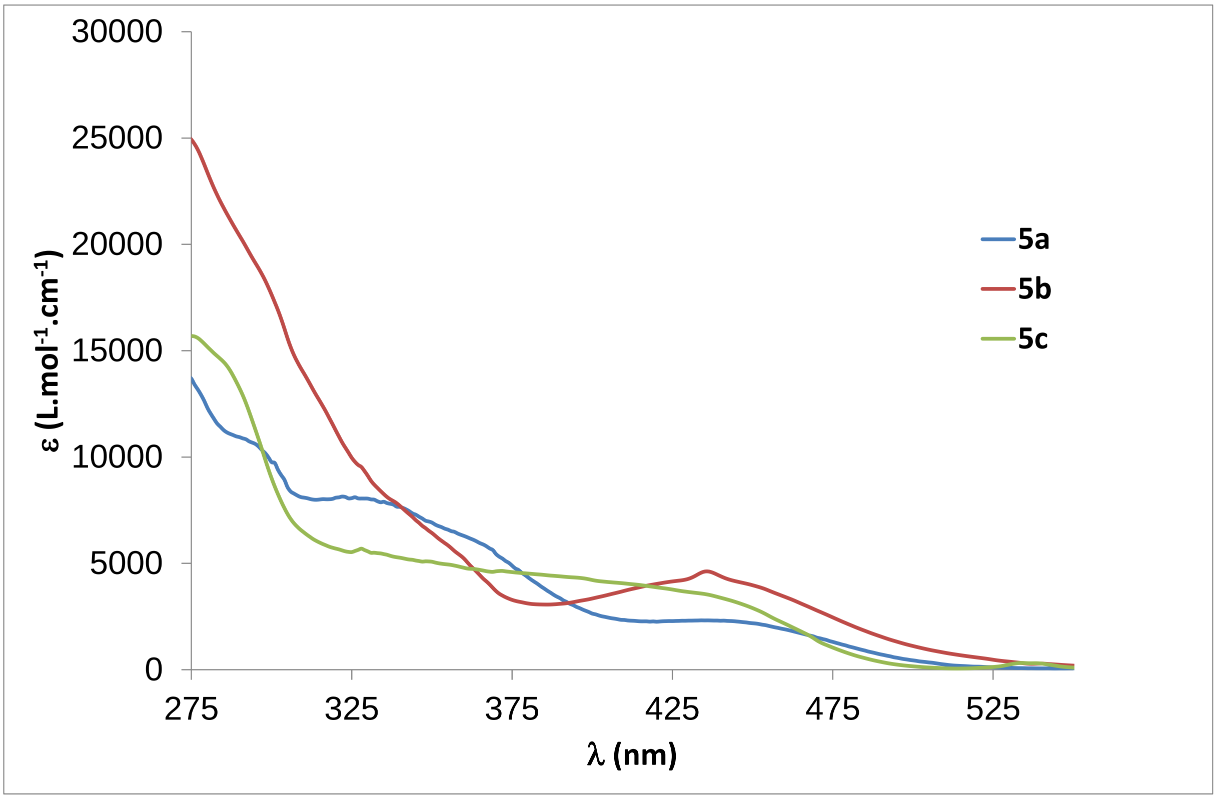

Figure S27**:** UV-vis absorption of **5a-5c** in diluted DCM (c= 10^{-5} M)

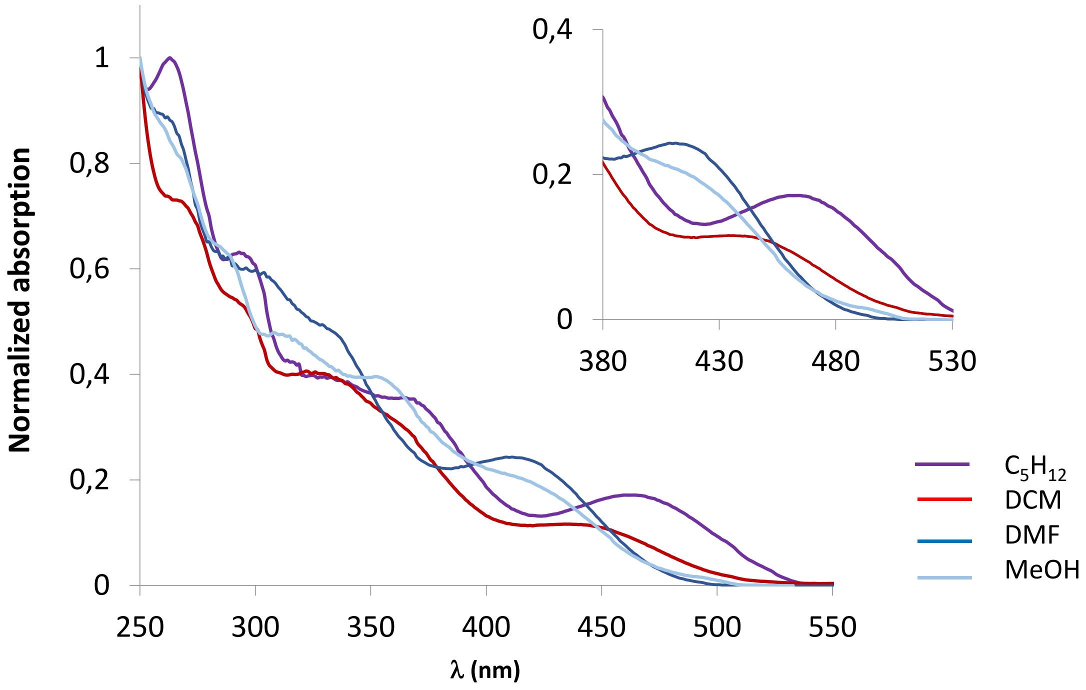

Figure S28: UV-vis absorption of **5a** in various solvents (c= 10^{-5} M)

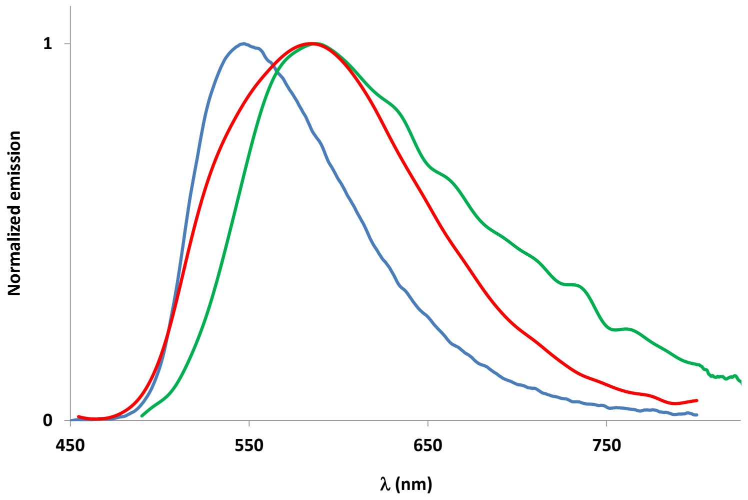

Figure S29: Solid state luminescence of **5a**, **7a** and **9**

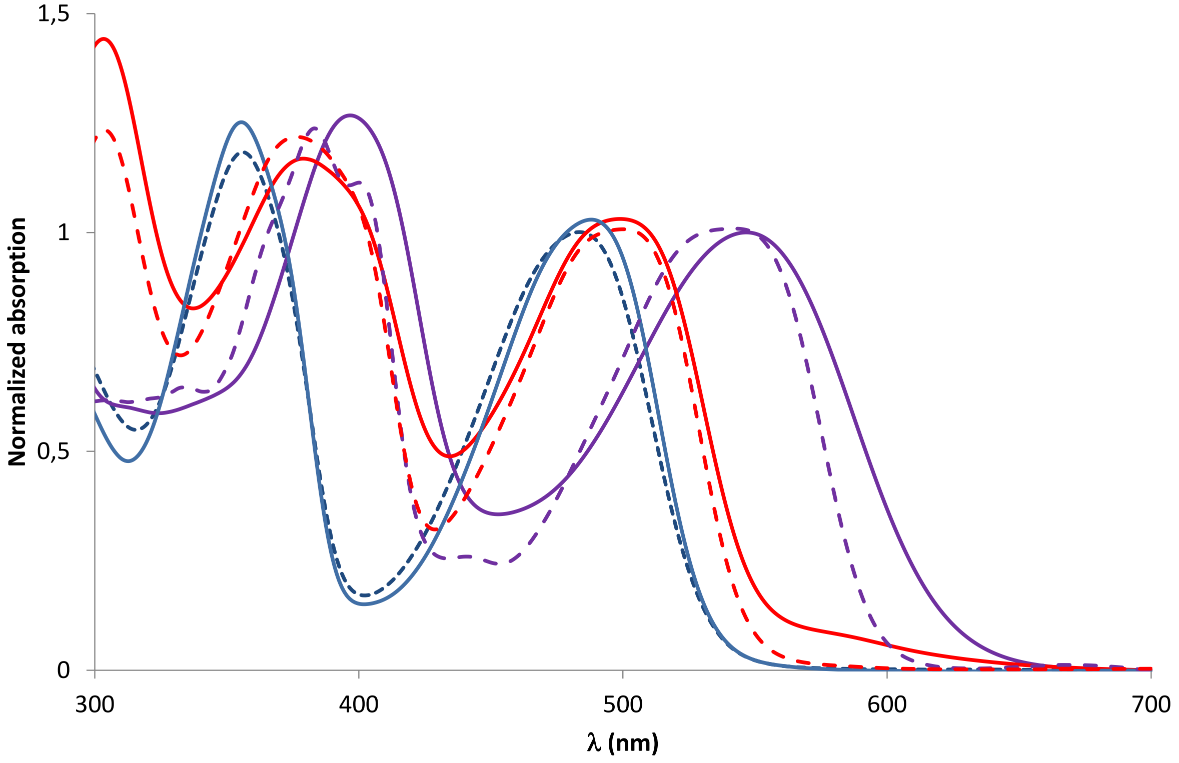

Figure S30: UV-vis absorption of **10** (purple), **11** (blue) and **12** (red) in DCM (solid line) and C5 (dashed line) (c= 10^{-5} M)

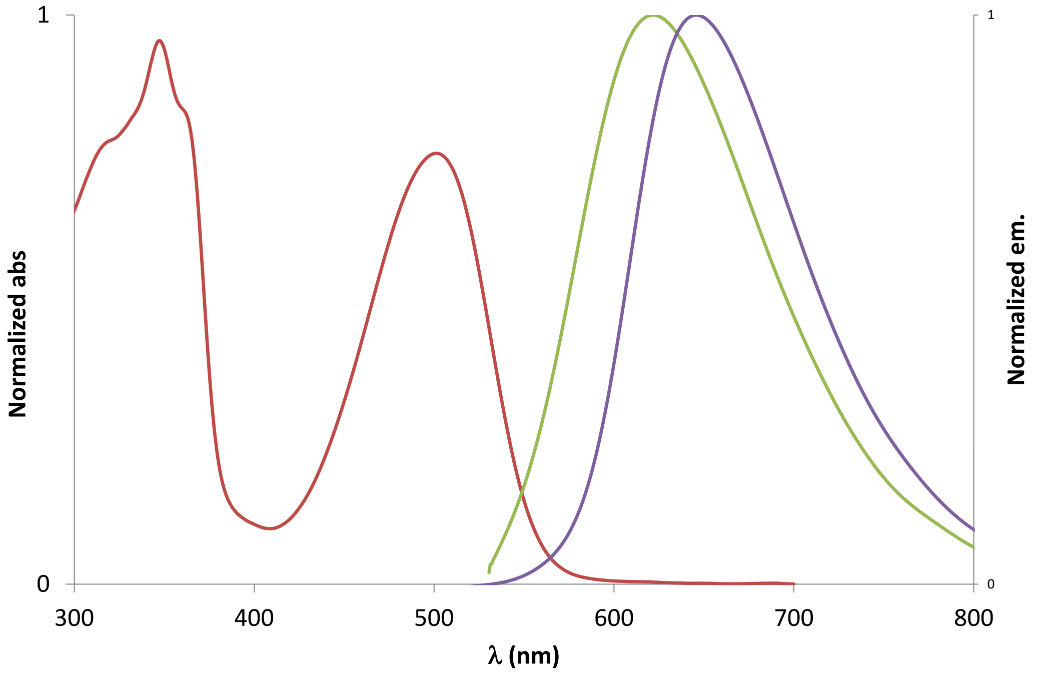

Figure S31: Normalized absorption (red), normalized emission (green) in diluted C5 (10^{-5} M) and normalized emission in powder of **14** (purple).

Nonlinear optical experiments

All solvent used for photophysical measurements were of spectrometric grade. The absolute quantum yields were measured with a C9920–03 Hamamatsu system. Emission spectra by two photon (2PE) absorption were recorded using a femtosecond laser chain (Ti-Sapphire Chameleon ultra II Coherent + pulse picker + SHG module when needed, pulse duration: 100-130 fs; pulse frequency: 5 MHz) and an Ocean optics QEPro CCD detector with integrating times ranging from 1 to 20s. The excitation beam crossed a lens before arriving on the sample and a short-pass 750 nm filter after the sample to remove the excitation signal and prevent damages on the CCD detector. The power of the beam was measured with a PMD100 console and a S142C integrating sphere sensor from Thorlabs. For emission intensity vs excitation power measurements, a λ/2 waveplate and a polarizer were used to vary the laser power. The 2PE was recorded in solution, perpendicularly to the beam using an optical fiber connected to a CCD detector.
To validate the experimental set-up, the quadraticity (I vs P^2) was first verified on a Rhodamine B solution at 8.25. 10^{-5} M in MeOH.
Two-photon absorption cross-section values were evaluated using Rhodamine B as standard ([RhB] = 8.25 10^{-5} M in MeOH using σ_2 reference values from Makarov *et al.*.[3];
In order to maximize the detection, hexane was chosen has the compounds display better luminescence in apolar solvent, and hexane has a higher boiling point than pentane. Finally, **12** was chosen as it displays both good luminescence and solubility in hexane. ([**12**] = 6.1 10^{-5} M in n-C_6H_{14})

[3] Makarov, N. S.; Drobizhev, M.; Rebane, A., Two-photon absorption standards in the 550–1600 nm excitation wavelength range. *Opt. Express* **2008,** *16* (6), 4029-4047

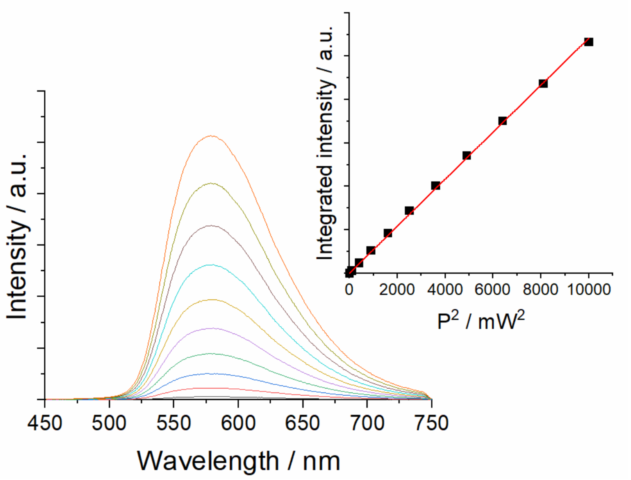

Figure S32. 2PE spectra vs irradiation power of **12** in hexane; inset: quadratic relationship of the observed two-photon emission intensity with the excitation laser power at 810 nm.

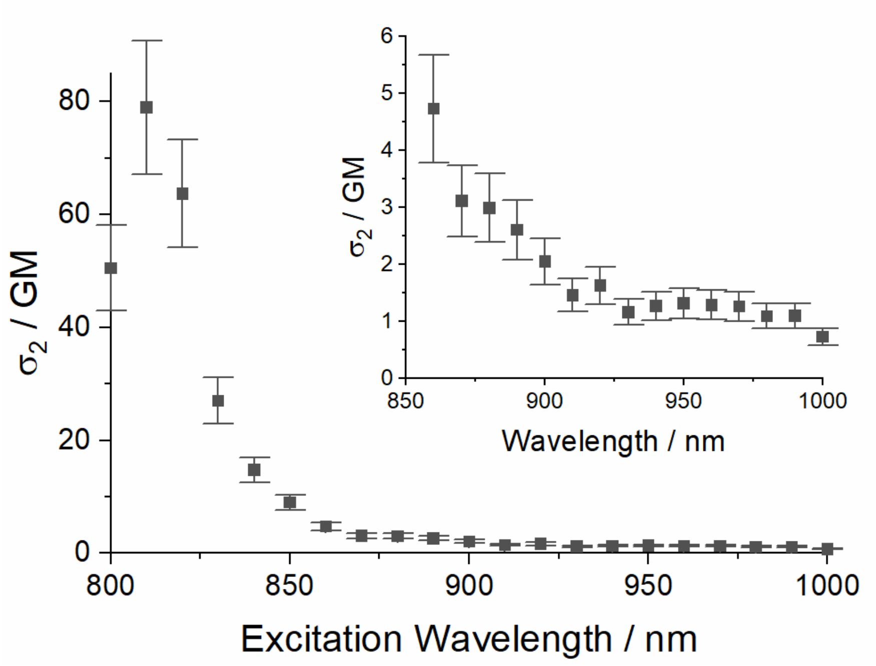

Figure S33. two photon absorption cross section values vs excitation wavelength (1GM = 10^{-50} cm^{4} s photon^{-1}) of **12**; error on calculated values is estimated as ±15%.

Electrochemical data

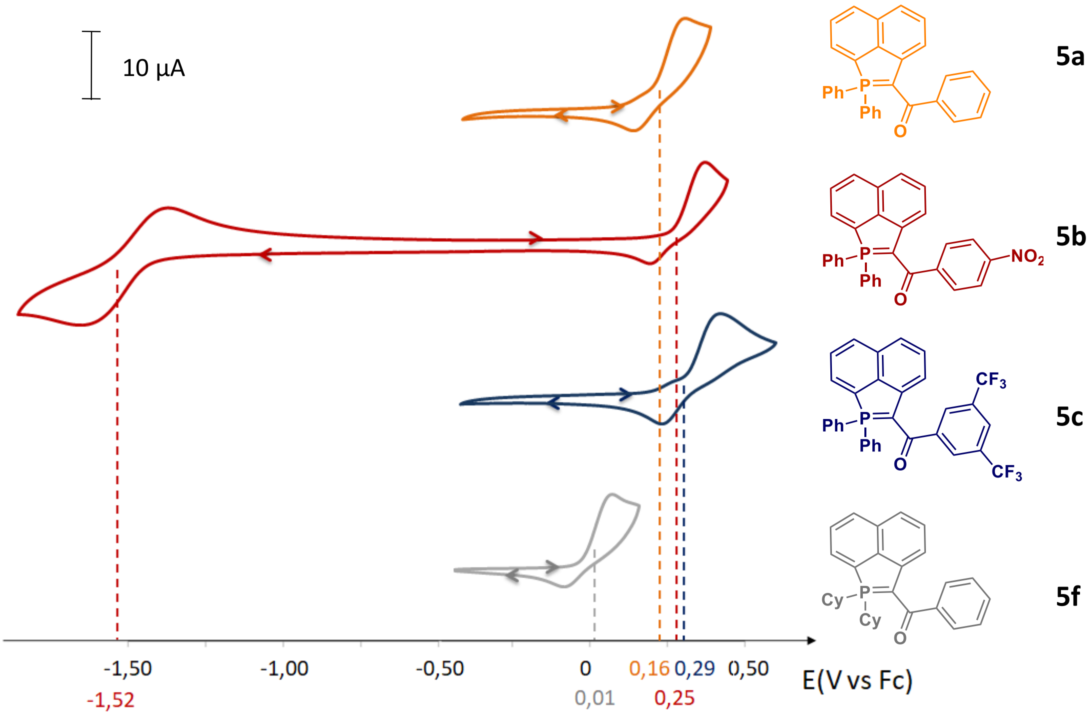

Figure S34: Cyclic voltammograms of **5a-5f** (c = 10^{-3} M) recorded in DCM (Bu_4NPF_6 (0.2 M), 200 mVs^{-1}, potentials vs Fc^{+}/Fc).

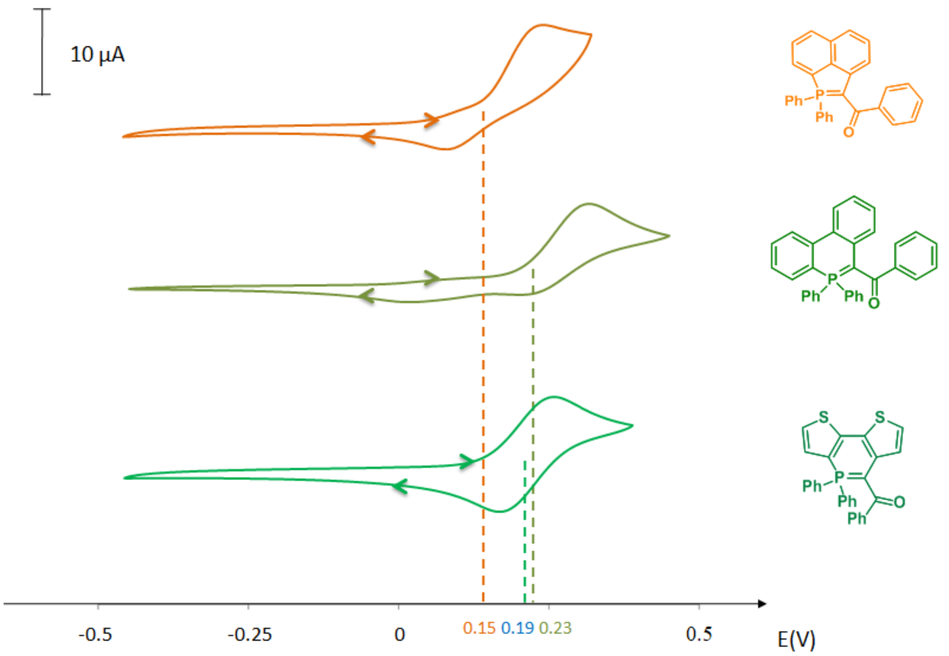

Figure S35: Cyclic voltammograms of **5a** (up),**7a** (middle), **9** (down) (c = 10^{-3} M) recorded in DCM (Bu_4NPF_6 (0.2 M), 200 mVs^{-1}, potentials vs Fc^{+}/Fc).

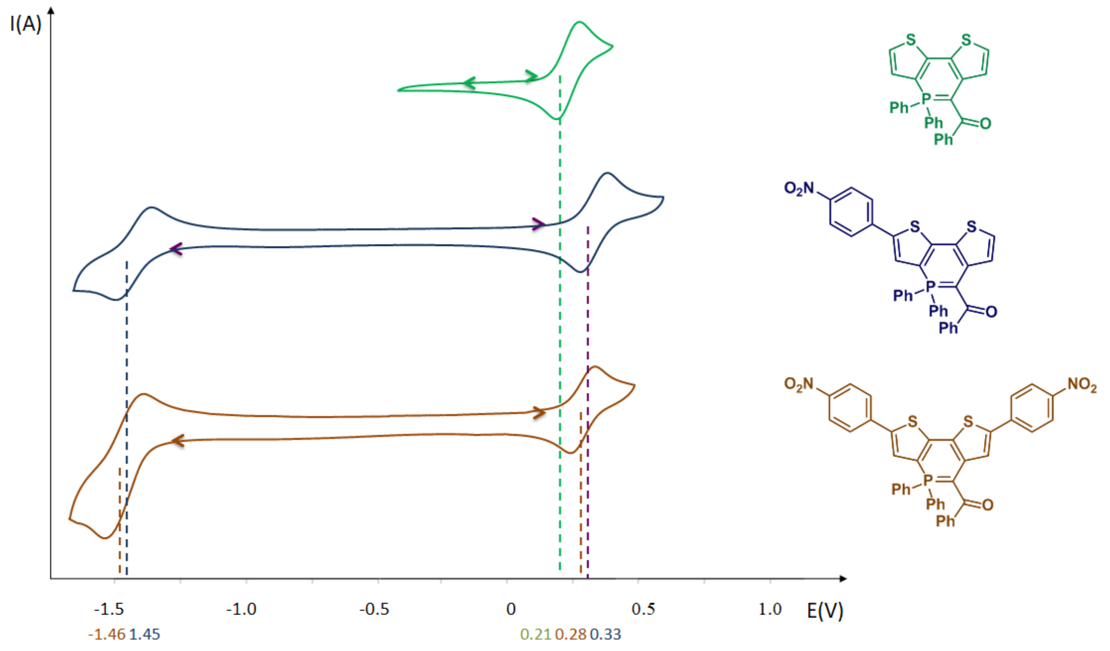

Figure S36: Cyclic voltammograms of **9, 10’** and **10** (c = 10^{-3} M) recorded in DCM (Bu_4NPF_6 (0.2 M), 200 mVs^{-1}, potentials vs Fc^+/Fc).

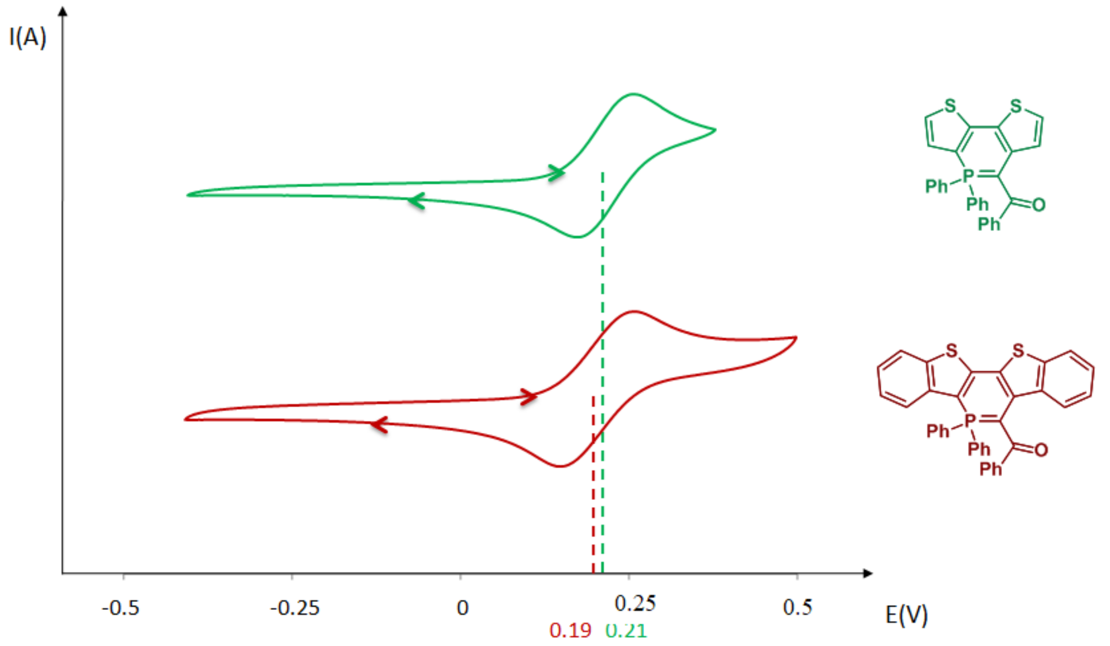

Figure S37 : Cyclic voltammograms of **9** and **14** (c = 10^{-3} M) recorded in DCM (Bu_4NPF_6 (0.2 M), 200 mVs^{-1}, potentials vs Fc^+/Fc).

Analytical chiral HPLC separation

• **16a** is dissolved in dichloromethane, injected on the chiral column, and detected with an UV detector at 254 nm and a circular dichroism detector at 254 nm. The flow-rate is 1 mL/min.

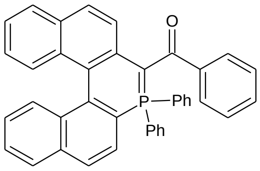

Column	Mobile Phase	t1	k1	t2	k2	α	Rs
**(*S*,*S*)-Whelk-O1**	Heptane / 2-PrOH / dichloromethane (50/20/30)	4.05 (+)	0.37	7.13 (-)	1.42	3.81	10

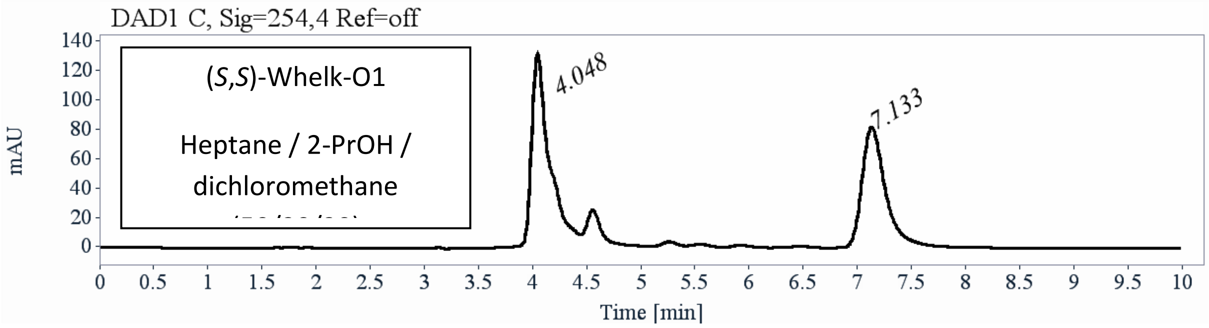

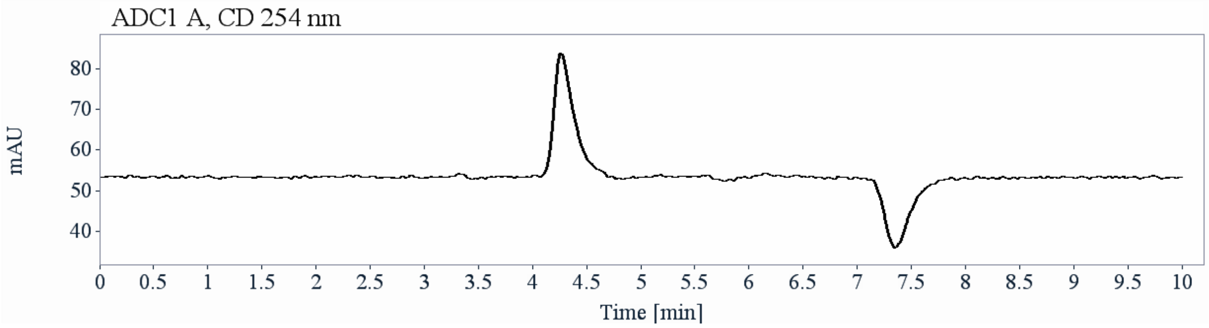

RT [min]	Area	Area%	Capacity Factor	Enantioselectivity	Resolution (USP)
4.05	1533	54.77	0.37		
7.13	1266	45.23	1.42	3.81	10.00
Sum	2799	100.00			

Preparative separation for compound **DT565**:

• Sample preparation: About 40 mg of compound **DT565** are dissolved in 8 mL of a mixture of dichloromethane/hexane (50/50).

• Chromatographic conditions: (*S*,*S*)-Whelk-O1 (250 x 10 mm), Hexane / 2-PrOH / dichloromethane (50/20/30) as mobile phase, flow-rate = 5 mL/min, UV detection at 254 nm.

• Injections (stacked): 20 times 400 μL, every 8 minutes.

• First fraction: 17 mg of the first eluted enantiomer with ee > 99.5%

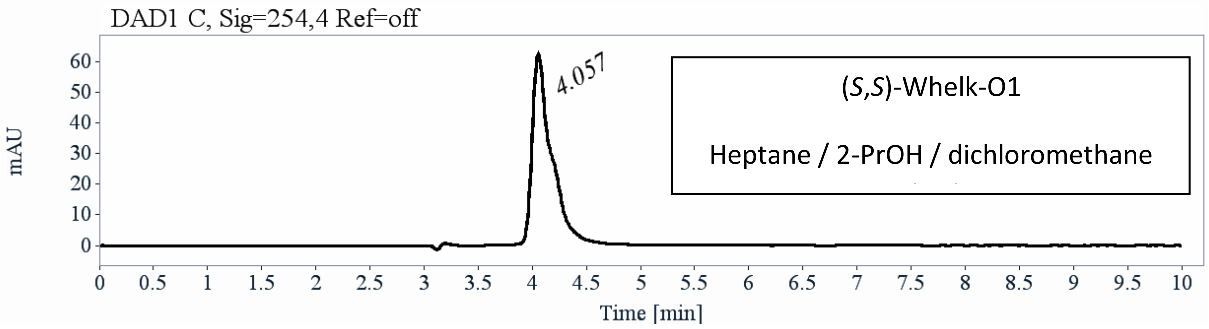

RT [min]	Area	Area%
4.06	785	100.00
Sum	785	100.00

• Second fraction: 14 mg of the second eluted enantiomer with ee > 99.5 %

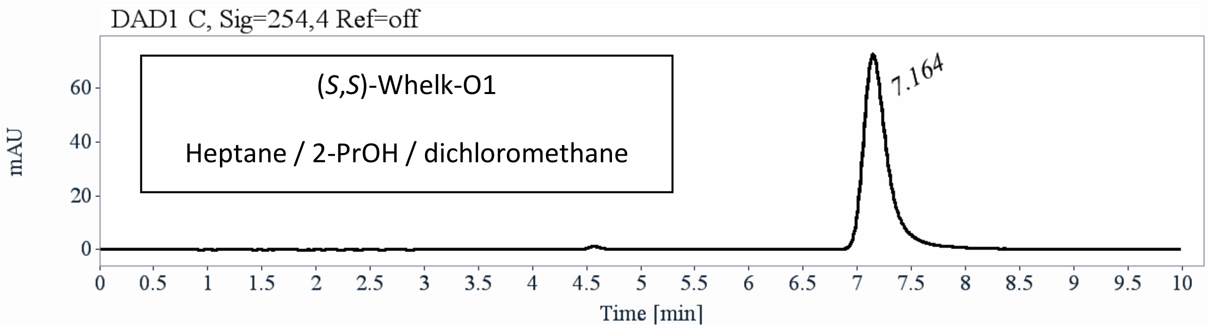

RT [min]	Area	Area%
7.16	1137	100.00
Sum	1137	100.00

• Third fraction: 14 mg of intermediate

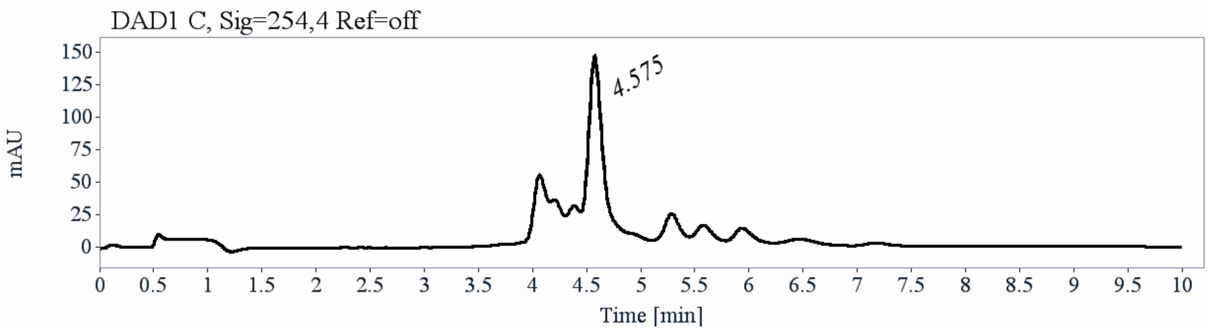

Kinetic of enantiomerization of **16a** in chlorobenzene

About 0.3 mg of the second eluted enantiomer of **16a** is heated in about 15 mL of chlorobenzene at 132°C. 20 µL are taken and then injected on (*S,S*)-Whelk-O1 (50:20:30 heptane / 2-PrOH / dichloromethane, 1 mL/min, UV 290 nm). The percentage decrease of the second eluted enantiomer of DT565 is monitored.

Time (min)	% second eluted enantiomer	ln ((%t-50%)/(%(t=0)-50%))
0	95.59	0
5	93.74	-0.041425379
10	91.73	-0.0884681
15	90.06	-0.129310064
20	88.41	-0.171370552
25	86.81	-0.213918847
30	84.92	-0.266628663
35	83.65	-0.303675338
40	81.83	-0.359279153
45	80.33	-0.407551073
50	78.77	-0.460355217
55	77.72	-0.49753422

Time (minutes)

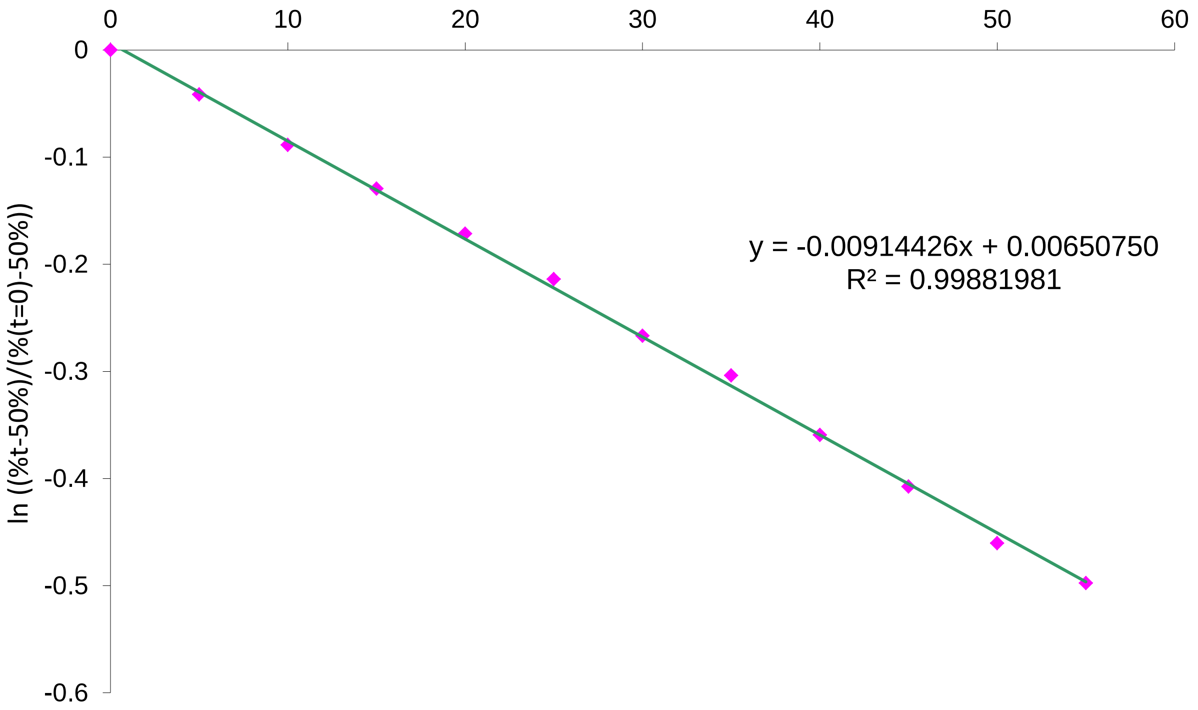

Fig S38: Kinetic of enantiomerization of **16a** in chlorobenzene

$k_{enantiomerisation}$ = 7.62.10^{-5} s^{-1} (132°C, chlorobenzene)

$\Delta G^{\neq}$ = 132.2 kJ.mol^{-1} (132°C, chlorobenzene)

$t_{1/2}$ = 76 minutes (132°C, chlorobenzene)

Theory

Methods

We have performed the DFT and TD-DFT calculations with Gaussian 16.[1] Default Gaussian 16 thresholds and algorithms were used but for an improved optimization threshold (10^{-5} au on average residual forces), a stricter self-consistent field convergence criterion (10^{-10} a.u.) and the use of the *ultrafine* DFT integration grid. Firstly, the S_0 geometries have been optimized with DFT and the vibrational frequencies have been analytically determined, using the M06-2X *meta*-GGA hybrid exchange-correlation functional.[2] These calculations were performed with the 6-31G(d) atomic basis set and account for solvent effects through the linear-response PCM approach considering DCM as solvent.[3] Secondly, starting from the optimal ground-state geometries, we have used TD-DFT with the same functional and basis set to optimize the S_1 geometry and compute the vibrational frequencies. All optimized structures correspond to true minima of the potential energy surface. Thirdly, the vertical transition energies were determined with TD-DFT and the same functional, but a larger basis set, namely 6-311+G(2d,p), in gas-phase as well as in solution using the cLR^2 variant of the PCM,[4] in its *non-equilibrium* limit. The selection of M06-2X for computing the lowest energy excited state is justified by numerous benchmarks on organic derivatives demonstrating that this functional offers one of the best compromise with high-correlations and rather low average deviations with respect to trustworthy experiments.[5] We use Le Bahers' d_{CT} model[6] on the so-called relaxed TD-M06-2X densities to quantify CT. The full UV/Vis and ECD spectra have been modelled with LR-PCM-B3LYP/6-311+G(2d,p), using a convoluting Gaussian of HWHM of 0.2 eV. The selection of B3LYP rather than M06-2X in this context is explained by the fact that the shape of ECD spectra depends on numerous excited states and that B3LYP globally provides a good balance for this task.[7] Ref. 7 concludes: "Overall for ECD and OR, 'traditional' GHs seem to provide the best choice". The TPA cross-sections have been obtained using QR-TD-DFT, with the CAM-B3LYP[8] range-separated hybrid functional and the 6-31G atomic basis set, with the Dalton code,[9] under its 2016 version. Default Dalton parameters were used to obtain the cross sections. Using a range-separate hybrid is typically necessary for modelling non-linear optical properties, and CAM-B3LYP is a typical choice for TPA.[7] Eventually, NICS(0) and NICS(1) values have been computed at the usual reference B3LYP/6-311+G(d,p)[10] level on the PCM-M06-2X/6-31G(d) geometries.

[1] M. J. Frisch, et al. Gaussian 16, revision A.03; Gaussian Inc.: Wallingford, CT, **2016.**

[2] Y. Zhao, D. G. Truhlar, *Theor. Chem. Acc.* **2008**, *120*, 215−241.

[3] J. Tomasi, B. Mennucci, R. Cammi, *Chem. Rev*. **2005**, *105*, 2999−3094.

[4] C. A. Guido, A. Chrayteh, G. Sclamani, B. Mennucci, D. Jacquemin *J. Chem. Theory Comput.* **2021**, *17*, 5155−5164.

[5] See for instance: a) S. S. Leang, F. Zahariev, M. S. Gordon, *J. Chem. Phys.* **2012**, *136*, 10401; b) D. Jacquemin, A. Planchat, C. Adamo, B. Mennucci, , *J. Chem. Theory Comput.* **2012**, *8*, 2350−2372; c) M. Isegawa, R. Peverati, D. G. Truhlar, *J. Chem. Phys.* **2012**, *137*, 244204.

[6] T. Le Bahers, C. Adamo, I. Ciofini, *J. Chem. Theory Comput.* **2011**, *7*, 2498−2506.

[7] Previous TD-DFT benchmarks and a summary of their conclusions can be found in: A. D. Laurent, D. Jacquemin, *Int. J. Quantum Chem.* **2013**, *113*, 2019-2039.

[8] T. Yanai, D. P. Tew, N. C. Handy, *Chem. Phys. Lett.* **2004**, *393*, 51–57.

[9] K. Aidas, et al. *WIREs Comput. Mol. Sci.* **2014**, *4*, 269–284.

[10] Z. Chen, C. S. Wannere, C. Corminboeuf, R. Puchta, P. von Ragué Schleyer *Chem. Rev.* **2005**, *105*, 3842–3888.

Structures of 5a and 7a

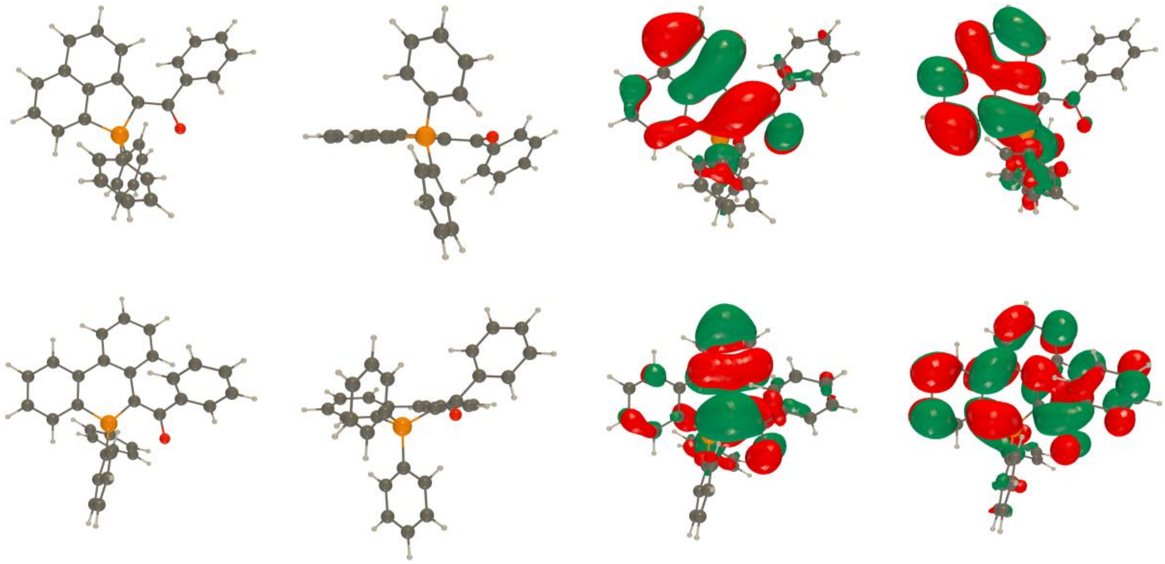

Top view *side view* *HOMO* *LUMO*

Figure S39: Top and side views of **5a** (top) and **7a** (bottom), as obtained by), as obtained by PCM-M06-2X/6-311+G(2d,p) on PCM-M06-2X/6-31G(d) geometries. Representation of the frontier MOs for these two compounds at the same level of theory.

Density differences plots of 5a, 7a, and 9

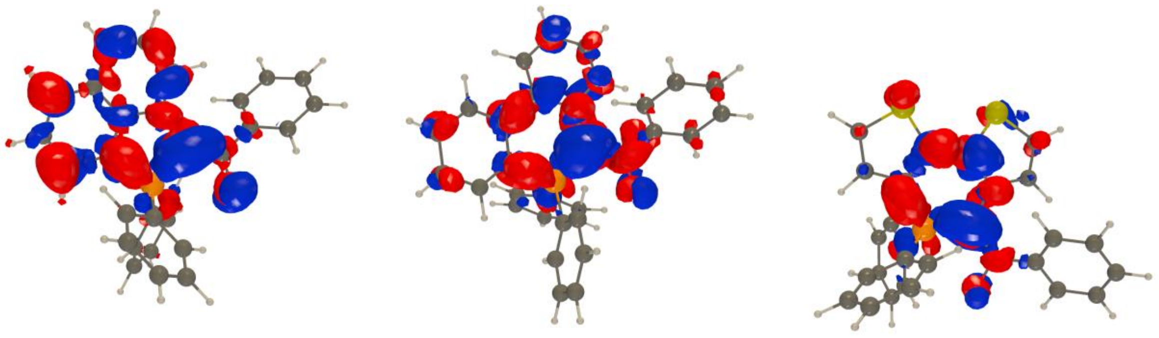

5a **7a** **9**

Figure S40: Density difference plots of **5a**, **7a**, and **9** obtained at the PCM-M06-2X/6-311+G(2d,p) level of theory for the lowest excited-transition (S_0-S_1). The blue and red lobes correspond to regions of decrease and increase of electron density upon excitation (absorption), respectively. Contour threshold: 15 x 10^{-4}.

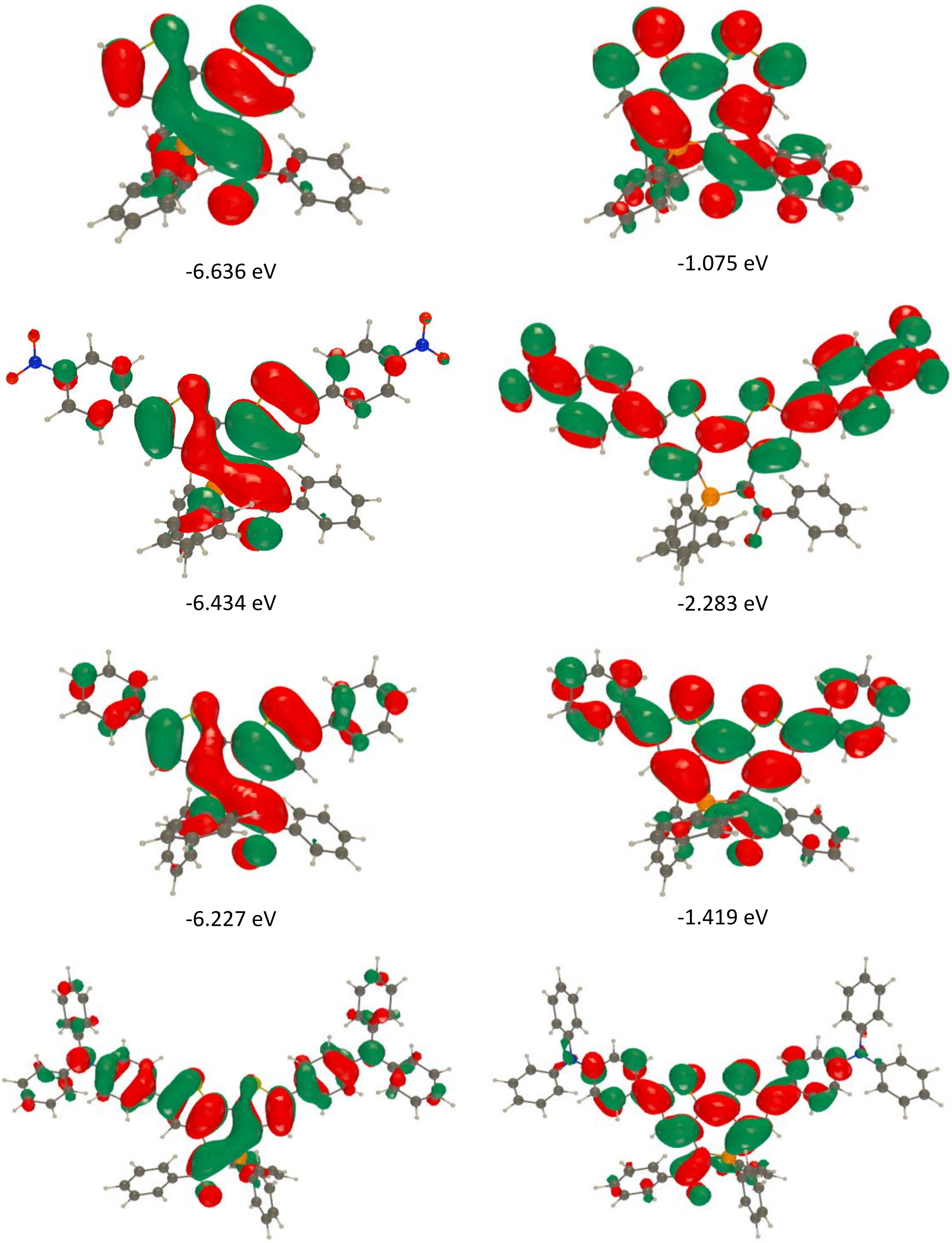

Fig. S41: Representation of the frontier MOs for **9-12**, as obtained by PCM-M06-2X/6-311+G(2d,p) on PCM-M06-2X/6-31G(d) geometries.

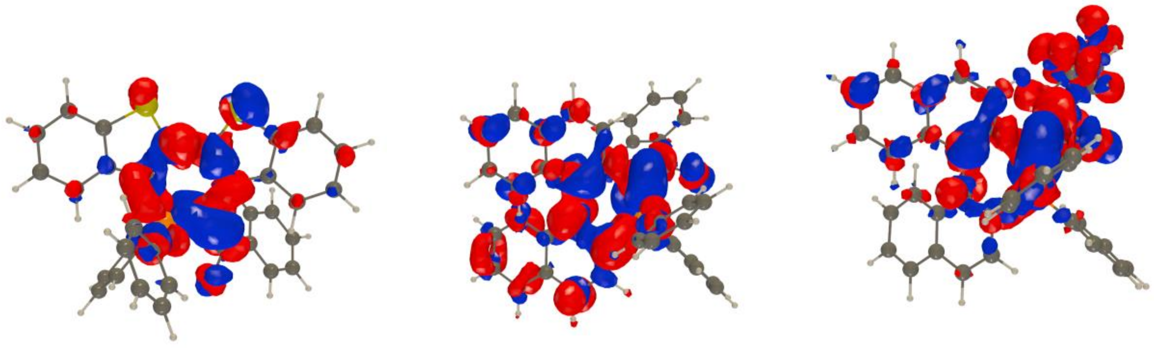

14 **16a** **16b**

Figure S42: Density difference plots of **14**, **16a**, and **16b** obtained at the PCM-M06-2X/6-311+G(2d,p) level of theory for the lowest excited-transition (*S_0-S_1*).. The blue and red lobes correspond to regions of decrease and increase of electron density upon excitation (absorption), respectively. Contour threshold: 1 x 10^{-3}

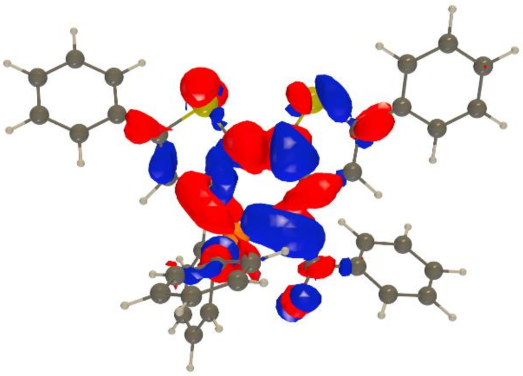

Figure S43: Density difference plots **11** obtained at the PCM-M06-2X/6-311+G(2d,p) level of theory for the lowest excited-transition (*S_0-S_1*).. The blue and red lobes correspond to regions of decrease and increase of electron density upon excitation (absorption), respectively. Contour threshold: 1 x 10^{-3}.

14

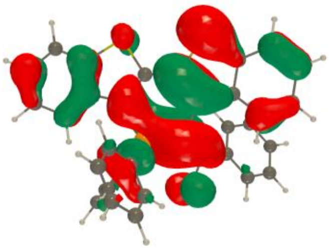

-6.321 eV

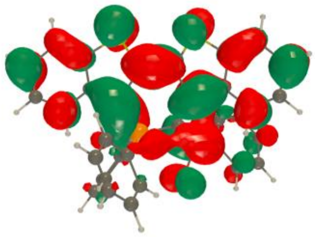

-1.493 eV

16a

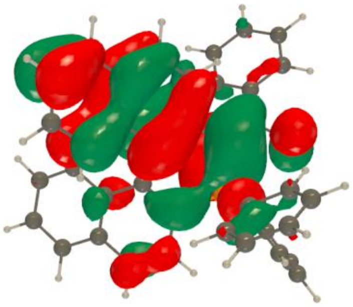

-6.366 eV

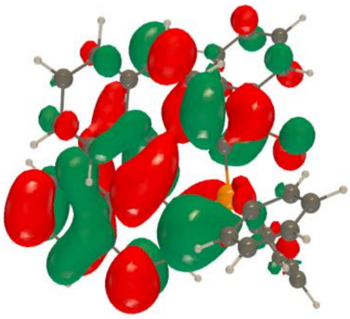

-1.278 eV

HOMO LUMO

Fig. S44: Representation of the frontier MOs for **14-16a**, as obtained by PCM-M06-2X/6-311+G(2d,p) on PCM-M06-2X/6-31G(d) geometries.

TD-DFT values

Table S3: TD-DFT data determined for selected dyes. We report the vertical absorption and emission, together with the associated oscillator strength. The transition energies are obtained at the cLR^2(neq)-PCM(DCM)-TD-M06-2X/6-311+G(2d,p) level, the oscillator strength ate the correspond LR(neq)-values. In the rightmost column, we provide the CT distance (for absorption) as given by Le Bahers' model.

Dye	Vert abs (nm)	*f* abs	Vert fl (nm)	*f* fl	d^{CT} (Å)
**5a**	407	0.15	577	0.13	1.97
**7a**	372	0.24	952[a]	0.02[a]	0.86
**9**	401	0.30	550	0.24	0.90
**10**	491	0.76	635	0.88	2.20
**11**	439	0.57	545	0.60	1.23
**12**	451	1.03	563	1.11	0.63

**14**	452	0.34	569	0.30	0.88
**16a**	415	0.24	540	0.23	1.64
**16b**	446	0.25	> 2000[a]	0.01[a]	2.79

[a]Values indicating a likely dark state quenching and a strong relaxation of the excited-state geometry.

Modeling of the UV/Vis and ECD spectra of 16a

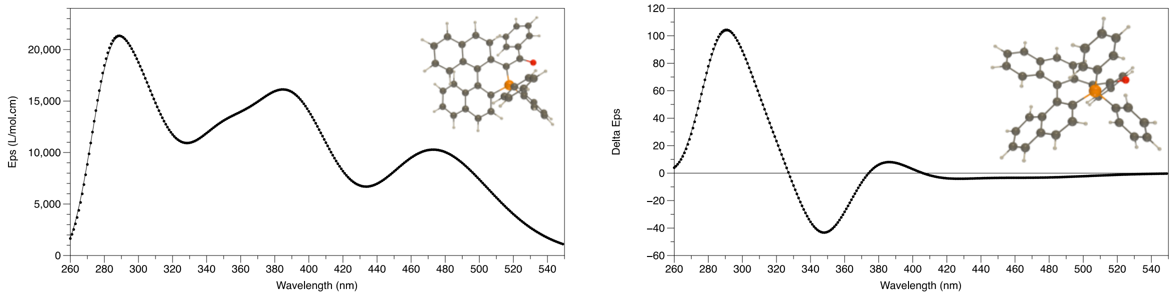

Figure S45: UV/Vis and ECD spectra modelled for **16a**. The stick (vertical) contributions computed at LR-PCM-B3LYP/6-311+G(2d,p) level have been convoluted with a Gaussian of HWHM of 0.2 eV.

Two-photon spectra of 12

Table S4: One and two-photon excitation obtained with (QR-)TD-DFT (CAM-B3LYP/6-31G) for dye **12.** We report the (one-photon) transition energy, the corresponding oscillator strength as well as the TPA cross section.

Transition	Vert abs (nm)	*f* abs	σ^{TPA} (GM)
S_0-S_1	421	0.96	6
S_0-S_2	333	0.84	96
S_0-S_3	313	0.36	217
S_0-S_4	308	0.15	120

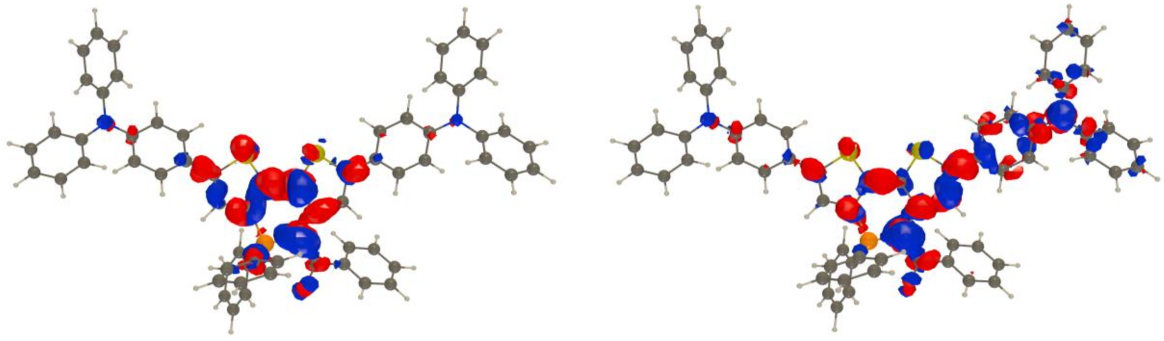

S_0-S_1 S_0-S_2

Figure S46: Density difference plots (CAM-B3LYP/6-31G) of **12**: two lowest excited-state. The blue and red lobes correspond to regions of decrease and increase of electron density upon excitation (absorption), respectively. Contour threshold: 10 x 10^{-4}. Note that for the lowest one, the topology is totally similar to the one reported in the main text with the M06-2X functional and a much larger basis set.

NICS Calculations.

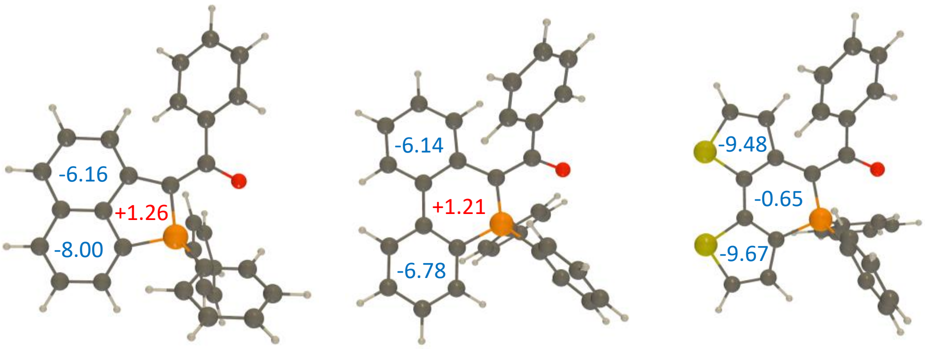

5a **7a** **9**

Fig. S47: NICS(0) aromaticity pattern of **5a**, **7a** and **9** at the B3LYP/6-311+G(d,p) level on the PCM-M06-2X/6-31G(d) geometries.

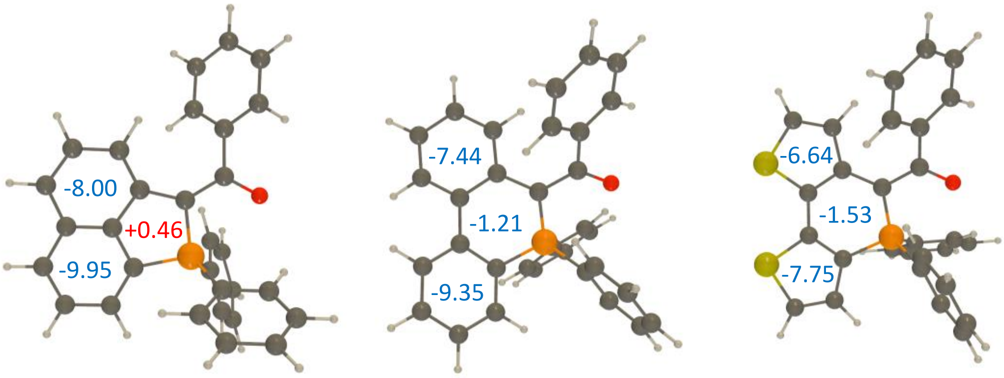

5a **7a** **9**

Fig. S48: NICS(1) aromaticity pattern of **5a**, **7a** and **9** at the B3LYP/6-311+G(d,p) level on the PCM-M06-2X/6-31G(d) geometries.

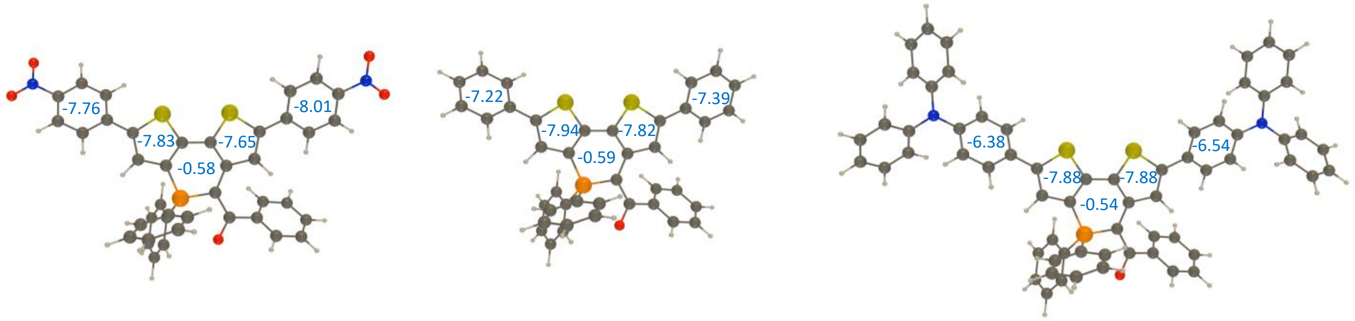

10 **11** **12**

Fig. S48: NICS(0) aromaticity pattern of **10**, **11** and **12** at the B3LYP/6-311+G(d,p) level on the PCM-M06-2X/6-31G(d) geometries.

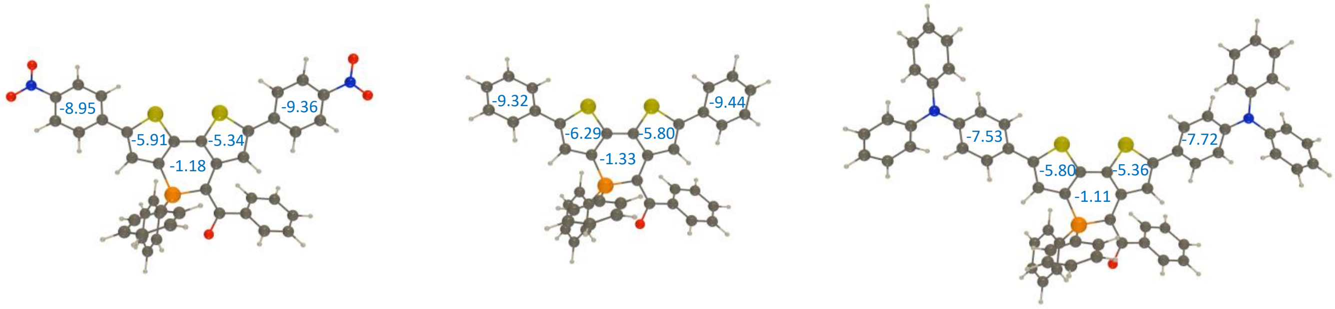

10 **11** **12**

Fig. S49: NICS(1) aromaticity pattern of **10**, **11** and **12** at the B3LYP/6-311+G(d,p) level on the PCM-M06-2X/6-31G(d) geometries.

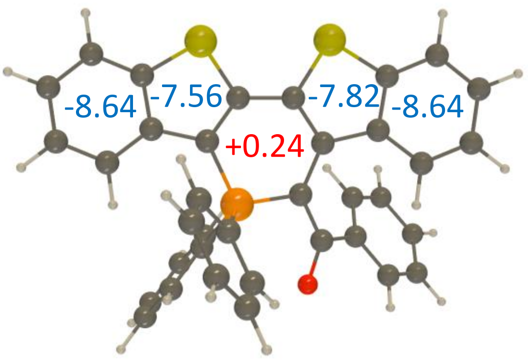

14

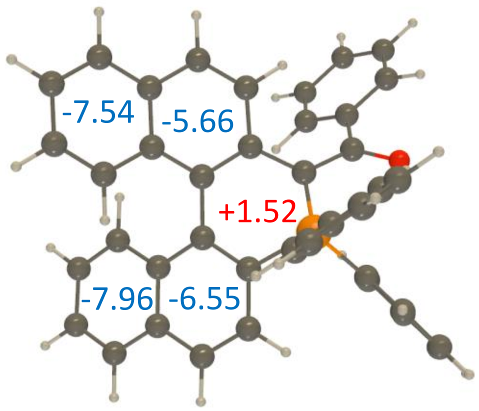

16a

Fig. S50: NICS(0) aromaticity pattern of **14** and **16a** at the B3LYP/6-311+G(d,p) level on the PCM-M06-2X/6-31G(d) geometries.

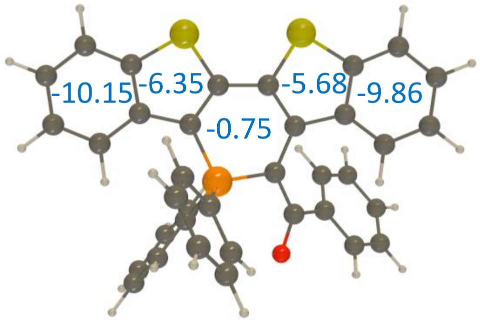

14

Internal
External

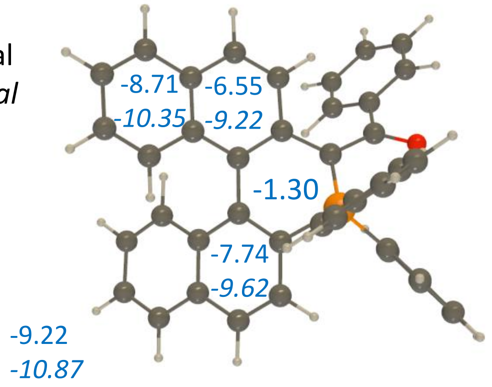

16a

Fig. S51: NICS(0) aromaticity pattern of **14** and **16a** at the B3LYP/6-311+G(d,p) level on the PCM-M06-2X/6-31G(d) geometries.

Cartesian coordinates *(ground state in Å)*

5a

P	0.9540180	-0.1081680	0.0457130
O	-0.8731120	-2.1825120	-0.5969640
C	1.9799670	-0.6555990	-1.3486250
C	2.6604500	0.2790460	-2.1344580
H	2.6038200	1.3376190	-1.8997590
C	3.4173540	-0.1512990	-3.2221870
H	3.9472940	0.5751940	-3.8295210
C	3.4943770	-1.5080930	-3.5244840
H	4.0856680	-1.8414320	-4.3715690
C	2.8122840	-2.4406420	-2.7424530
H	2.8703020	-3.4976330	-2.9813940
C	2.0530100	-2.0206130	-1.6560580
H	1.5041490	-2.7370830	-1.0537130
C	1.4083240	-1.1462220	1.4592000
C	2.7316400	-1.5626160	1.6346350
H	3.4869240	-1.3205050	0.8915520
C	3.0786910	-2.2993890	2.7636840
H	4.1052870	-2.6225640	2.9019040
C	2.1073570	-2.6265240	3.7080930
H	2.3792310	-3.2065140	4.5843570
C	0.7876240	-2.2145200	3.5296310
H	0.0313240	-2.4752300	4.2627630
C	0.4354850	-1.4707580	2.4076680
H	-0.5900280	-1.1437600	2.2593320

C	1.2674960	1.6392890	0.3724110
C	2.4211200	2.3074340	0.7126700
H	3.3596240	1.7785760	0.8553150
C	2.3659080	3.7125170	0.8688850
H	3.2678710	4.2509920	1.1406780
C	1.1889180	4.4029200	0.6706120
H	1.1701880	5.4838390	0.7830850
C	-0.0107480	3.7328420	0.3145020
C	-1.2545380	4.3768360	0.0654620
H	-1.3301630	5.4561480	0.1557310
C	-2.3362460	3.6147260	-0.3026990
H	-3.2841010	4.1022010	-0.5125910
C	-2.2745410	2.2013560	-0.4233250
H	-3.1667170	1.6656960	-0.7260180
C	-1.0981850	1.5234680	-0.1553820
C	0.0450520	2.3295280	0.1853020
C	-0.7721700	0.1001680	-0.1905380
C	-1.4836300	-1.1058760	-0.4209610
C	-2.9855720	-1.1498970	-0.3945650
C	-3.7201300	-0.4632900	0.5752770
H	-3.2010010	0.1685180	1.2914960
C	-5.1060490	-0.5927590	0.6282540
H	-5.6699530	-0.0641710	1.3906780
C	-5.7660700	-1.4030910	-0.2938480
H	-6.8470010	-1.4983040	-0.2573660
C	-5.0361340	-2.0992010	-1.2573360
H	-5.5479360	-2.7370820	-1.9714040

C	-3.6501750	-1.9848150	-1.2962750
H	-3.0653670	-2.5414660	-2.0220940

7a

P	0.9445480	-0.1889150	0.0478520
C	1.9892760	-0.2453810	1.5370680
C	3.0578360	0.6376630	1.7105110
H	3.2970730	1.3663640	0.9415140
C	3.8177080	0.5832460	2.8778490
H	4.6474800	1.2695460	3.0126440
C	3.5100210	-0.3468420	3.8669380
H	4.1030730	-0.3867840	4.7752730
C	2.4386520	-1.2249760	3.6964970
H	2.1980350	-1.9459320	4.4712460
C	1.6737290	-1.1770270	2.5365480
H	0.8297740	-1.8464940	2.3916470
C	1.2649810	-1.7180400	-0.8761900
C	0.3937720	-2.0669330	-1.9130270
H	-0.4891820	-1.4615690	-2.1022790
C	0.6535000	-3.1924290	-2.6853670
H	-0.0275460	-3.4664620	-3.4845130
C	1.7848800	-3.9688070	-2.4314620
H	1.9861220	-4.8470190	-3.0367570
C	2.6541130	-3.6199340	-1.4022390
H	3.5332470	-4.2235270	-1.2012850
C	1.5063480	1.1624200	-1.0031400
C	2.6571290	0.9805320	-1.7823000

H	3.2230730	0.0562510	-1.6964680
C	2.3959330	-2.4947810	-0.6199100
H	3.0726250	-2.2295850	0.1872300
C	3.0704950	1.9619630	-2.6693810
H	3.9648530	1.8195400	-3.2662920
C	2.3012310	3.1189400	-2.8026270
H	2.5878860	3.8825190	-3.5189210
C	1.1570700	3.2967420	-2.0400650
H	0.5599120	4.1870190	-2.1998920
C	0.7379420	2.3404200	-1.0957190
C	-0.4597370	2.5798030	-0.2588880
C	-0.8908100	3.9020590	-0.0531890
H	-0.3075760	4.7191820	-0.4641730
C	-2.0194650	4.2028630	0.6893500
H	-2.3216400	5.2351700	0.8310050
C	-2.7365790	3.1590630	1.2782500
H	-3.6107680	3.3686110	1.8875790
C	-2.3074170	1.8528730	1.1286790
H	-2.8408060	1.0611690	1.6429700
C	-1.1770250	1.5141980	0.3510190
C	-0.7510540	0.1184810	0.2621090
C	-1.5284090	-1.0485900	0.5661040
O	-1.0090780	-2.1032200	0.9773150
C	-3.0063150	-1.0748670	0.2739850
C	-3.5633050	-0.3642820	-0.7936870
H	-2.9255460	0.2681640	-1.4049470
C	-4.9215730	-0.4744740	-1.0756800

H	-5.3459600	0.0715090	-1.9125940
C	-5.7357600	-1.2862310	-0.2861260
H	-6.7966150	-1.3662300	-0.5031140
C	-5.1848870	-1.9998090	0.7772780
H	-5.8156080	-2.6362620	1.3904000
C	-3.8230020	-1.9026170	1.0476540
H	-3.3731610	-2.4671640	1.8583700

9

C	1.6487760	1.1746540	-0.6135220
C	2.9578210	1.3102140	-1.1835550
H	3.6533900	0.4865760	-1.3032840
C	3.2318320	2.5859030	-1.5582740
H	4.1340600	2.9729560	-2.0104600
C	0.9482920	2.3690250	-0.5733250
C	-0.3728440	2.5318750	-0.0522710
C	-1.1749970	1.4672700	0.3497350
C	-2.4148450	1.9603540	0.9041370
H	-3.1831940	1.3225940	1.3202110
C	-2.5218570	3.3129640	0.8757740
H	-3.3456850	3.9165200	1.2312420
C	-0.7653570	0.0810340	0.2660580
C	-1.5529150	-1.0940890	0.5053760
O	-1.0062680	-2.1818890	0.7658750
C	-3.0466650	-1.0861980	0.3433750
C	-3.6679180	-0.3844230	-0.6940060
H	-3.0670840	0.2254160	-1.3637220

C	-5.0464280	-0.4679940	-0.8680370
H	-5.5223750	0.0710760	-1.6811750
C	-5.8134620	-1.2430750	0.0007600
H	-6.8896560	-1.3009730	-0.1302290
C	-5.1975460	-1.9496790	1.0331940
H	-5.7930080	-2.5578960	1.7069780
C	-3.8168780	-1.8829880	1.1935900
H	-3.3189110	-2.4458140	1.9770580
C	1.9103950	-0.8645500	1.4415810
C	2.7041230	0.0752190	2.1058100
H	2.7568810	1.0981950	1.7429260
C	3.4321060	-0.3006390	3.2327750
H	4.0492110	0.4308440	3.7443640
C	3.3679060	-1.6118250	3.6964680
H	3.9363440	-1.9046710	4.5737100
C	2.5734120	-2.5501810	3.0369380
H	2.5226010	-3.5713830	3.4009500
C	1.8412970	-2.1818060	1.9133710
H	1.2087440	-2.9011630	1.4040400
C	1.1428620	-1.6090810	-1.2678140
C	2.3429690	-2.3192820	-1.3578000
H	3.1229550	-2.1776960	-0.6147620
C	2.5373900	-3.2237550	-2.3999580
H	3.4701440	-3.7742910	-2.4670390
C	1.5354960	-3.4237570	-3.3459190
H	1.6864890	-4.1325060	-4.1539830
C	0.3378930	-2.7148370	-3.2559940

H	-0.4451540	-2.8719920	-3.9906620
C	0.1426240	-1.8041900	-2.2233200
H	-0.7872840	-1.2461380	-2.1520820
P	0.9210940	-0.3280910	0.0055860
S	1.9058300	3.6568970	-1.2382110
S	-1.1268070	4.0805940	0.2084760

10

S	-1.8140660	2.2660620	-0.1647810
S	1.5436920	2.3482920	0.0262930
P	-0.8488720	-1.8994790	0.2598990
O	1.0425160	-3.9212990	0.2281500
O	-9.3791540	3.1135190	0.0331600
O	-8.5160620	4.8417240	-0.9222590
O	8.1957250	5.0796660	0.7578960
O	9.0926590	3.3066530	-0.0760310
N	-8.4305860	3.7373920	-0.4121080
N	8.1309150	3.9438590	0.3189560
C	-1.6344760	-2.9943690	-0.9588960
C	-1.7632230	-4.3669870	-0.7219800
H	-1.3979150	-4.7904500	0.2074640
C	-2.3457770	-5.1814590	-1.6877520
H	-2.4451380	-6.2462940	-1.5042660
C	-2.7949800	-4.6330930	-2.8883690
H	-3.2490530	-5.2725640	-3.6387850
C	-2.6613380	-3.2673930	-3.1284870
H	-3.0099990	-2.8394090	-4.0625820

C	-2.0812660	-2.4461480	-2.1655490
H	-1.9782430	-1.3806570	-2.3527560
C	-1.2806180	-2.5560730	1.9011270
C	-0.3110140	-2.5335060	2.9047580
H	0.6939350	-2.1912890	2.6713460
C	-0.6353660	-2.9571610	4.1905300
H	0.1199680	-2.9459440	4.9695450
C	-1.9266340	-3.3978800	4.4736860
H	-2.1782990	-3.7279570	5.4765930
C	-2.8961120	-3.4206950	3.4718460
H	-3.8998840	-3.7694920	3.6916540
C	-2.5755310	-3.0022270	2.1837780
H	-3.3293790	-3.0397880	1.4012730
C	0.8630650	-1.6116770	0.0065280
C	1.3965530	-0.2708270	0.0179380
C	0.6072730	0.8806930	-0.0041510
C	-0.8163700	0.8529580	-0.0276970
C	-1.5977060	-0.2909510	0.0707390
C	2.7858220	0.0891710	0.1076530
H	3.5834770	-0.6332590	0.2175070
C	3.0213620	1.4337660	0.1020370
C	-2.9954810	-0.0130130	0.0331450
H	-3.7604490	-0.7821180	0.0605460
C	-3.2817330	1.3182770	-0.0926660
C	1.5793680	-2.8530840	-0.1111690
C	2.9568130	-2.9073020	-0.7060090
C	3.9120260	-3.7385400	-0.1180380

H	3.6424190	-4.3065700	0.7671820
C	5.1923990	-3.8248610	-0.6579990
H	5.9377280	-4.4579790	-0.1869470
C	5.5136350	-3.1035980	-1.8070570
H	6.5100120	-3.1752410	-2.2320830
C	4.5519500	-2.2976680	-2.4172940
H	4.7949130	-1.7504790	-3.3228550
C	3.2784700	-2.1947780	-1.8649910
H	2.5278830	-1.5604920	-2.3289450
C	-4.5985380	1.9537020	-0.1731600
C	-5.7264920	1.2938350	0.3432710
H	-5.6138870	0.3321520	0.8317930
C	-6.9842330	1.8684670	0.2615220
H	-7.8593730	1.3690530	0.6578680
C	-7.1059000	3.1211640	-0.3295440
C	-6.0129430	3.8099650	-0.8398880
H	-6.1491200	4.7830770	-1.2946380
C	-4.7616610	3.2178180	-0.7614340
H	-3.9059230	3.7394720	-1.1789900
C	4.3279230	2.0959150	0.1580950
C	5.4699000	1.4137770	-0.2937690
H	5.3719550	0.4170150	-0.7126320
C	6.7192830	2.0106570	-0.2368610
H	7.6070820	1.4963930	-0.5828520
C	6.8149860	3.3044970	0.2629990
C	5.7063160	4.0148590	0.7063370
H	5.8243140	5.0204620	1.0898530

C	4.4638350	3.4015030	0.6534580
H	3.5927490	3.9375950	1.0176440

11

S	1.7956160	-1.8114670	-0.1069380
S	-1.5738410	-1.8800510	0.0220280
P	0.8328210	2.3520610	0.2893960
O	-1.0486740	4.3880490	0.1875810
C	1.6587970	3.4488980	-0.9023540
C	1.7913420	4.8192210	-0.6553300
H	1.4020370	5.2409650	0.2651150
C	2.4074980	5.6340900	-1.5998070
H	2.5092810	6.6973560	-1.4083340
C	2.8873290	5.0881370	-2.7896400
H	3.3677690	5.7277310	-3.5234310
C	2.7506860	3.7245660	-3.0399410
H	3.1236990	3.2981300	-3.9653520
C	2.1368280	2.9033130	-2.0980170
H	2.0325350	1.8390940	-2.2917290
C	1.2224330	3.0121730	1.9413000
C	0.2171830	3.0304920	2.9089790
H	-0.7884860	2.7164770	2.6414120
C	0.5076340	3.4584900	4.2016090
H	-0.2754330	3.4790550	4.9526260
C	1.8007700	3.8626060	4.5275010
H	2.0260800	4.1957580	5.5356860
C	2.8062560	3.8444180	3.5615340

H	3.8119620	4.1640340	3.8146860
C	2.5193050	3.4218010	2.2670030
H	3.3016260	3.4266480	1.5118690
C	-0.8721000	2.0751660	-0.0142370
C	-1.4136980	0.7347610	-0.0069770
C	-0.6276670	-0.4168840	-0.0024010
C	0.7973460	-0.3943920	0.0078500
C	1.5819020	0.7442120	0.1197350
C	-2.8070140	0.3825900	0.0584630
H	-3.6040520	1.1095170	0.1422000
C	-3.0515980	-0.9600880	0.0624780
C	2.9822480	0.4614570	0.1049790
H	3.7506880	1.2268230	0.1413810
C	3.2673700	-0.8690730	-0.0137600
C	-1.5763130	3.3181380	-0.1647190
C	-2.9288770	3.3769940	-0.8154400
C	-3.8928120	4.2395690	-0.2899380
H	-3.6503730	4.8246180	0.5920190
C	-5.1467130	4.3366570	-0.8870590
H	-5.8991080	4.9948450	-0.4638510
C	-5.4321730	3.5939530	-2.0318720
H	-6.4075720	3.6738500	-2.5018390
C	-4.4610080	2.7555410	-2.5798190
H	-4.6752870	2.1903150	-3.4815610
C	-3.2148900	2.6426570	-1.9700750
H	-2.4574880	1.9837620	-2.3859380
C	4.5830910	-1.5148110	-0.0678990

C	5.6968950	-0.8928020	0.5144200
H	5.5719210	0.0456330	1.0461100
C	6.9517810	-1.4806580	0.4497180
H	7.7935300	-0.9902890	0.9299370
C	7.1409540	-2.7160950	-0.1844190
C	6.0245230	-3.3413770	-0.7528830
H	6.1454030	-4.2872590	-1.2726800
C	4.7685850	-2.7515200	-0.6995010
H	3.9284660	-3.2469870	-1.1789950
C	-4.3608790	-1.6214200	0.0952470
C	-5.4894810	-0.9607060	-0.4110790
H	-5.3779370	0.0206830	-0.8640920
C	-6.7387900	-1.5631760	-0.3726160
H	-7.5936730	-1.0420600	-0.7936840
C	-6.9058870	-2.8492870	0.1586370
C	-5.7744040	-3.5109250	0.6519340
H	-5.8795270	-4.4980040	1.0924910
C	-4.5238910	-2.9081730	0.6238550
H	-3.6690000	-3.4331250	1.0419400
C	-8.2423170	-3.4916940	0.1937850
C	-8.3820270	-4.8682870	-0.0247680
C	-9.3930490	-2.7340500	0.4469360
C	-9.6362800	-5.4699990	0.0092390
H	-7.5032030	-5.4671360	-0.2466740
C	-10.6475210	-3.3352290	0.4801870
H	-9.3006000	-1.6695430	0.6429790
C	-10.7738930	-4.7056850	0.2615630

H	-9.7256330	-6.5369860	-0.1700930
H	-11.5270840	-2.7328490	0.6854300
H	-11.7523180	-5.1749240	0.2876630
C	8.4838070	-3.3423380	-0.2489790
C	8.6330480	-4.7312700	-0.1452130
C	9.6312360	-2.5564290	-0.4165040
C	9.8934980	-5.3175170	-0.2076660
H	7.7564360	-5.3538990	0.0097960
C	10.8918740	-3.1422080	-0.4780460
H	9.5318160	-1.4799030	-0.5234850
C	11.0277340	-4.5251900	-0.3740860
H	9.9902490	-6.3950780	-0.1177730
H	11.7689650	-2.5174920	-0.6159570
H	12.0109500	-4.9824780	-0.4223520

12

S	-1.7739540	1.0545620	-0.0735960
S	1.5971440	1.1179260	0.0108210
P	-0.8122290	-3.1106570	0.2871120
O	1.0655320	-5.1500860	0.1546340
C	-1.6540760	-4.2032460	-0.8979440
C	-1.7841470	-5.5744910	-0.6548030
H	-1.3834510	-5.9999760	0.2590190
C	-2.4125140	-6.3854520	-1.5946340
H	-2.5122770	-7.4494500	-1.4060980
C	-2.9071090	-5.8346760	-2.7761590
H	-3.3969590	-6.4712110	-3.5063980

C	-2.7732240	-4.4701240	-3.0226160
H	-3.1578350	-4.0398680	-3.9415070
C	-2.1472000	-3.6528590	-2.0852170
H	-2.0451830	-2.5878020	-2.2755420
C	-1.1816350	-3.7783740	1.9410240
C	-0.1631170	-3.8065130	2.8944460
H	0.8401630	-3.4959350	2.6141820
C	-0.4374680	-4.2398440	4.1888480
H	0.3559330	-4.2680280	4.9287180
C	-1.7278480	-4.6395040	4.5308380
H	-1.9406380	-4.9768050	5.5403740
C	-2.7466540	-4.6114650	3.5791510
H	-3.7503050	-4.9274370	3.8448110
C	-2.4757490	-4.1834490	2.2829150
H	-3.2686780	-4.1801450	1.5389020
C	0.8893120	-2.8357960	-0.0371110
C	1.4338610	-1.4958620	-0.0309130
C	0.6483890	-0.3443920	-0.0100920
C	-0.7761950	-0.3653160	0.0197480
C	-1.5621850	-1.5022930	0.1341490
C	2.8283760	-1.1459710	0.0203220
H	3.6257880	-1.8739470	0.0908520
C	3.0755200	0.1966470	0.0301560
C	-2.9629580	-1.2173370	0.1358190
H	-3.7321270	-1.9818710	0.1746990
C	-3.2477440	0.1144010	0.0284560
C	1.5893820	-4.0789730	-0.2006750

C	2.9334000	-4.1370680	-0.8692730
C	3.9034210	-5.0024320	-0.3598440
H	3.6721480	-5.5903670	0.5232070
C	5.1491510	-5.0987810	-0.9740110
H	5.9065130	-5.7590770	-0.5630880
C	5.4200610	-4.3525150	-2.1200280
H	6.3890590	-4.4316800	-2.6031880
C	4.4424480	-3.5113610	-2.6520240
H	4.6452320	-2.9432230	-3.5546120
C	3.2048090	-3.3991570	-2.0251050
H	2.4426640	-2.7378580	-2.4282190
C	-4.5596010	0.7664550	-0.0026680
C	-5.6707890	0.1506730	0.5919230
H	-5.5460770	-0.7928290	1.1150380
C	-6.9221590	0.7451660	0.5622850
H	-7.7643020	0.2577250	1.0426960
C	-7.1087280	1.9843620	-0.0680460
C	-6.0023160	2.6077060	-0.6605540
H	-6.1295240	3.5622750	-1.1605540
C	-4.7520770	2.0084150	-0.6223590
H	-3.9184100	2.5045060	-1.1126430
C	4.3831700	0.8579050	0.0478410
C	5.5047470	0.2098850	-0.4904830
H	5.3874000	-0.7651160	-0.9561300
C	6.7536590	0.8104290	-0.4785580
H	7.6047270	0.2982900	-0.9155000
C	6.9263550	2.0862560	0.0788120

C	5.8087010	2.7408630	0.6151570
H	5.9259240	3.7240360	1.0589020
C	4.5608150	2.1362400	0.5926790
H	3.7158400	2.6562880	1.0366310
N	-8.3801830	2.5906430	-0.0996290
N	8.1952350	2.6981070	0.0943870
C	-8.5029470	4.0011290	-0.0096970
C	-7.7134160	4.7253840	0.8906050
C	-9.4221130	4.6806950	-0.8166270
C	-7.8359230	6.1086050	0.9700330
H	-7.0055710	4.1983070	1.5229910
C	-9.5510030	6.0620800	-0.7167090
H	-10.0328060	4.1193480	-1.5170570
C	-8.7568180	6.7847760	0.1717580
H	-7.2160810	6.6578500	1.6719720
H	-10.2690090	6.5762110	-1.3481790
H	-8.8550060	7.8630730	0.2418400
C	-9.5485570	1.7951120	-0.2188430
C	-9.5788880	0.7077880	-1.0994020
C	-10.6855320	2.0954670	0.5395150
C	-10.7260480	-0.0713620	-1.2069980
H	-8.7001650	0.4791350	-1.6946650
C	-11.8344400	1.3220170	0.4113380
H	-10.6614880	2.9373090	1.2246290
C	-11.8610390	0.2322780	-0.4572090
H	-10.7351270	-0.9125640	-1.8932750
H	-12.7096040	1.5671550	1.0051420

H	-12.7567690	-0.3730680	-0.5495160
C	8.3152060	4.1033570	-0.0610690
C	9.2178910	4.8235770	0.7289970
C	7.5409650	4.7810430	-1.0094590
C	9.3459480	6.1989160	0.5652280
H	9.8166870	4.2980740	1.4665560
C	7.6621140	6.1592580	-1.1530530
H	6.8458570	4.2218440	-1.6282590
C	8.5668460	6.8756340	-0.3714500
H	10.0511930	6.7449710	1.1841490
H	7.0541760	6.6724210	-1.8917560
H	8.6640700	7.9496080	-0.4916490
C	9.3663210	1.9155620	0.2640790
C	10.5095200	2.1869220	-0.4958830
C	9.3940420	0.8718940	1.1959790
C	11.6619950	1.4286360	-0.3187510
H	10.4874870	2.9949310	-1.2206470
C	10.5449310	0.1066110	1.3525140
H	8.5105010	0.6655730	1.7922600
C	11.6859970	0.3816820	0.6008880
H	12.5418940	1.6512020	-0.9144680
H	10.5517480	-0.7009930	2.0780310
H	12.5843520	-0.2126840	0.7312940

14

S	-1.5152150	3.3908720	-1.1803070
S	1.6219120	3.5429210	-0.1201210

P	-0.9073290	-0.5780960	0.3964370
O	1.0086860	-2.7042660	0.6400000
C	-0.6951610	2.0223140	-0.4604250
C	-1.5423710	0.9489180	-0.2680010
C	-2.8962240	1.2110940	-0.7097940
C	-4.0312970	0.3839800	-0.6690570
H	-3.9615250	-0.6229420	-0.2685030
C	-5.2422770	0.8625800	-1.1428550
H	-6.1180120	0.2226820	-1.1119710
C	-5.3524840	2.1616700	-1.6605280
H	-6.3098080	2.5171750	-2.0269910
C	-4.2478760	3.0004430	-1.7058700
H	-4.3272060	4.0079230	-2.1011740
C	-3.0306380	2.5154310	-1.2282330
C	0.6875950	2.0561000	-0.1173750
C	1.4044140	0.9314040	0.2638560
C	2.7612600	1.3069450	0.6584900
C	3.7605920	0.5235290	1.2609670
H	3.5707070	-0.5182240	1.4908680
C	4.9852600	1.0859010	1.5752730
H	5.7517400	0.4742640	2.0397290
C	5.2430300	2.4403210	1.3097590
H	6.2112580	2.8645400	1.5555040
C	4.2608170	3.2489470	0.7615730
H	4.4396100	4.3048830	0.5857650
C	3.0236900	2.6759790	0.4557710
C	-1.6467070	-0.8697890	2.0349950

C	-1.2456860	-1.9861350	2.7823760
H	-0.5108540	-2.6694390	2.3660040
C	-1.7849870	-2.1959780	4.0471070
H	-1.4766650	-3.0599820	4.6270060
C	-2.7149710	-1.2977060	4.5712920
H	-3.1319080	-1.4651840	5.5594370
C	-3.1036420	-0.1824940	3.8337890
H	-3.8208060	0.5209990	4.2440570
C	-2.5683960	0.0367140	2.5660510
H	-2.8657480	0.9133060	1.9976900
C	-1.4788300	-1.9204940	-0.6935510
C	-1.0022320	-1.9270120	-2.0079730
H	-0.2837100	-1.1761890	-2.3282270
C	-1.4402340	-2.8995960	-2.8999390
H	-1.0653070	-2.9051360	-3.9181950
C	-2.3594040	-3.8629220	-2.4853160
H	-2.7020210	-4.6205540	-3.1829070
C	-2.8384420	-3.8534400	-1.1780120
H	-3.5526700	-4.6029450	-0.8529240
C	-2.3995560	-2.8838070	-0.2784720
H	-2.7745070	-2.8829370	0.7405990
C	0.8365310	-0.3940190	0.3006900
C	1.5201000	-1.6614380	0.2009110
C	2.8044470	-1.7814570	-0.5723090
C	3.1103490	-0.9379390	-1.6435730
H	2.4215400	-0.1445950	-1.9198080
C	4.2905900	-1.1173460	-2.3599230

H	4.5197660	-0.4629800	-3.1951390
C	5.1746230	-2.1349760	-2.0065790
H	6.0987100	-2.2681410	-2.5607450
C	4.8680070	-2.9873230	-0.9456470
H	5.5521710	-3.7849710	-0.6732090
C	3.6815990	-2.8179350	-0.2404850
H	3.4196140	-3.4794610	0.5795170

16a

P	1.1386030	-0.8968340	0.3068720
O	3.2342960	1.1388640	0.3864210
C	-1.4794010	-0.7527790	-0.5074690
C	-0.3568510	-1.5723180	-0.4374500
C	-1.5646290	0.4932890	0.2867080
C	-0.4216400	1.2954100	0.4571160
C	1.3431100	-1.5423140	1.9906010
C	2.3849290	-1.0306750	2.7762110
H	3.0441460	-0.2726670	2.3596220
C	2.5563220	-1.4987100	4.0739310
H	3.3622920	-1.1082240	4.6868790
C	1.6913730	-2.4659530	4.5889550
H	1.8275610	-2.8265610	5.6037030
C	0.6517120	-2.9666370	3.8095980
H	-0.0229780	-3.7139520	4.2143510
C	0.4734570	-2.5050450	2.5064980
H	-0.3395050	-2.8899000	1.8966540
C	2.5148280	-1.5329110	-0.6854440

C	2.7356700	-0.9855270	-1.9537440
H	2.1292270	-0.1501590	-2.2947020
C	3.7373120	-1.5048800	-2.7646130
H	3.9134760	-1.0752340	-3.7453550
C	4.5151280	-2.5739320	-2.3174240
H	5.2962140	-2.9781090	-2.9535440
C	4.2919080	-3.1214680	-1.0577330
H	4.8966120	-3.9517170	-0.7077430
C	3.2915040	-2.6020220	-0.2368910
H	3.1230600	-3.0280980	0.7477360
C	0.9195760	0.8217140	0.1425730
C	2.1272820	1.5943160	0.0491340
C	2.0920770	2.9658480	-0.5715560
C	1.1472150	3.3185870	-1.5403840
H	0.3897930	2.5982310	-1.8362690
C	1.1852100	4.5792010	-2.1292320
H	0.4545460	4.8440090	-2.8872060
C	2.1620780	5.4987000	-1.7489700
H	2.1874230	6.4836760	-2.2050990
C	3.1099270	5.1502880	-0.7872830
H	3.8737660	5.8634840	-0.4929840
C	3.0798560	3.8851760	-0.2089260
H	3.8192660	3.5898960	0.5288570
C	-2.7947240	0.8594300	0.9484250
C	-3.8880970	-0.0373450	1.1126990
C	-2.9187140	2.1470450	1.5385260
C	-5.0428640	0.3488010	1.7500510

H	-3.8032230	-1.0537180	0.7453520
C	-4.1278560	2.5300260	2.1702250
C	-5.1814210	1.6556490	2.2679080
H	-5.8531980	-0.3645610	1.8659890
H	-4.1935880	3.5286010	2.5949560
H	-6.1022390	1.9524050	2.7596790
C	-2.5131530	-1.1332910	-1.4439450
C	-2.4665170	-2.4169230	-2.0598270
C	-3.5477310	-0.2424380	-1.8456690
C	-3.4975150	-2.8040740	-2.9569870
C	-4.5118940	-0.6303440	-2.7415880
H	-3.5598290	0.7659370	-1.4499570
C	-4.5055120	-1.9359970	-3.2860890
H	-3.4541270	-3.7970770	-3.3958700
H	-5.2820780	0.0733450	-3.0408730
H	-5.2831130	-2.2362280	-3.9812080
C	-1.3598680	-3.2765390	-1.8243020
C	-0.3010440	-2.8364240	-1.0834240
H	-1.3432590	-4.2567530	-2.2916940
H	0.5892320	-3.4502460	-0.9779940
C	-1.7869110	3.0093520	1.5420100
C	-0.5831870	2.5808180	1.0735550
H	-1.8784500	3.9916760	1.9985030
H	0.2898740	3.2129160	1.1891180

16b

P	-0.8709050	1.5619770	-0.1843530

O	1.8691770	2.5018140	-0.7221490
C	-1.7635880	-0.9002700	0.6394590
C	-1.9782970	0.4716680	0.7283590
C	-0.8990890	-1.4699730	-0.4199890
C	0.2770950	-0.8010300	-0.7991750
C	-1.6778420	2.1724470	-1.6880700
C	-0.9098020	2.9140440	-2.5959250
H	0.1371670	3.1104810	-2.3757020
C	-1.4989870	3.3866830	-3.7628690
H	-0.9111670	3.9633170	-4.4696440
C	-2.8430100	3.1163240	-4.0267530
H	-3.2979990	3.4850090	-4.9406900
C	-3.6017130	2.3723640	-3.1268340
H	-4.6444950	2.1589240	-3.3377610
C	-3.0205580	1.8963980	-1.9528120
H	-3.6074810	1.3113710	-1.2498860
C	-0.5750410	2.9857390	0.8951240
C	0.2340690	2.8110230	2.0229350
H	0.7177420	1.8533420	2.1985210
C	0.4243500	3.8668180	2.9055720
H	1.0575550	3.7336420	3.7766100
C	-0.1961610	5.0946790	2.6706030
H	-0.0463870	5.9175660	3.3622900
C	-1.0052580	5.2662120	1.5516090
H	-1.4874000	6.2205900	1.3669910
C	-1.1972790	4.2121340	0.6591110
H	-1.8249570	4.3504870	-0.2161790

C	0.5497060	0.5778390	-0.4082860
C	1.7914460	1.2816140	-0.4991110
C	3.0837590	0.5532170	-0.2070900
C	3.1395330	-0.5301400	0.6762770
H	2.2280050	-0.8952020	1.1385630
C	4.3549210	-1.1327030	0.9737000
H	4.4235720	-1.9671040	1.6602170
C	5.5019540	-0.6343140	0.3676020
C	5.4821810	0.4442660	-0.5087490
H	6.4040400	0.7996420	-0.9517990
C	4.2594960	1.0416230	-0.7837510
H	4.1998120	1.8979160	-1.4464580
C	-1.2880430	-2.6722940	-1.1176530
C	-2.5951380	-3.2308100	-1.0379910
C	-0.3619850	-3.3089540	-1.9889680
C	-2.9262940	-4.3782080	-1.7175300
H	-3.3525100	-2.7316510	-0.4445070
C	-0.7197940	-4.5046160	-2.6608120
C	-1.9746110	-5.0426800	-2.5231390
H	-3.9354420	-4.7715740	-1.6420490
H	0.0185800	-4.9747310	-3.3053770
H	-2.2451550	-5.9543950	-3.0459310
C	-2.3567260	-1.7268860	1.6654790
C	-3.3097620	-1.1606610	2.5604020
C	-1.9695100	-3.0800830	1.8772340
C	-3.9159890	-1.9787580	3.5509800
C	-2.5461760	-3.8375880	2.8650030

H	-1.1911260	-3.5102080	1.2585990
C	-3.5516340	-3.2915600	3.6972450
H	-4.6580380	-1.5316210	4.2066270
H	-2.2230920	-4.8628870	3.0139870
H	-4.0118090	-3.9063820	4.4641350
C	-3.6019670	0.2283480	2.4989550
C	-2.9039980	1.0350340	1.6466360
H	-4.3389530	0.6439620	3.1798270
H	-3.0534490	2.1110740	1.6612850
C	0.8952120	-2.6873340	-2.2247400
C	1.1752450	-1.4630580	-1.6989920
H	1.5999430	-3.1686570	-2.8979130
H	2.0893920	-0.9563120	-1.9880000
N	6.7898100	-1.2693550	0.6721050
O	6.7930490	-2.2121660	1.4447930
O	7.7883060	-0.8209090	0.1356740
